# Fluorinated *N*-Heterocyclic Carbene Silver(I) Complexes with High Cancer Cell Selectivity

**DOI:** 10.1021/acs.organomet.4c00292

**Published:** 2024-09-19

**Authors:** Oliver S. King, Benjamin J. Hofmann, Aran E. Boakye-Smith, Amy J. Managh, Tameryn Stringer, Rianne M. Lord

**Affiliations:** † School of Chemistry, Pharmacy and Pharmacology, University of East Anglia, Norwich, Norfolk NR1 1GE, United Kingdom; ‡ Department of Chemistry, School of Science, Loughborough University, Loughborough, Leicestershire LE11 3TU, United Kingdom

## Abstract

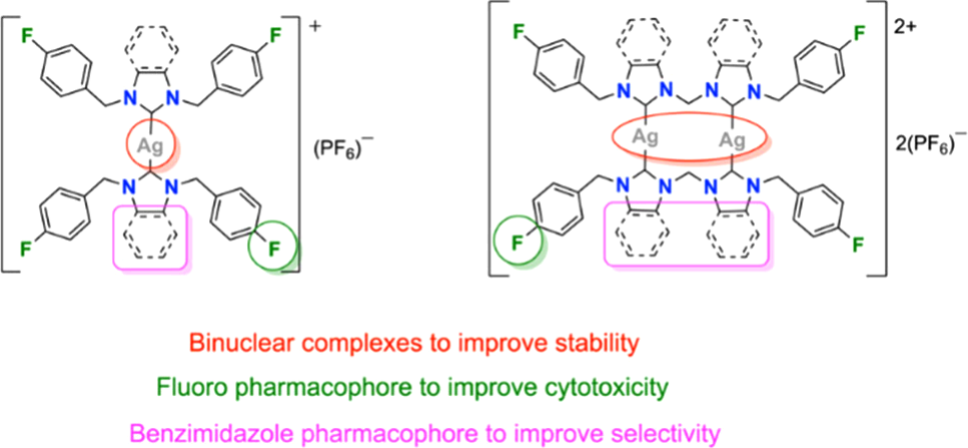

This work presents the synthesis of five new functionalized (benz)imidazolium *N*-heterocyclic (NHC) ligands (**L**) and four new (benz)imidazole silver(I) NHC (Ag(I)-NHC) complexes of mononuclear **[Ag(L)_2_](PF_6_)** or binuclear **[Ag_2_(L)_2_](PF_6_)_2_** type. The complexes have been fully characterized, including single crystal X-ray diffraction of three new structures. The complexes and their corresponding free NHC ligands have been screened against breast cancer and noncancerous cell lines, showing the mononuclear benzimidazole complex has the highest activity, while the binuclear benzimidazole complex has the highest cancer cell selectivity. The silver uptake was measured by ICP-MS and highlights a strong link between cytotoxicity and cellular uptake. DNA interaction studies, molecular docking, and evaluation of reactive oxygen species (ROS) have been conducted for the most promising complexes to identify modes of action. Overall, the binuclear benzimidazole complex is the most selective and promising candidate against the MDA-MD-231 (breast cancer) cell line and has potential to be developed for treatment of late-stage breast cancers.

## Introduction

In recent years, interest in medicinal inorganic chemistry has surged following the discovery of the anticancer properties of platinum-based therapeutics, e.g., cisplatin, carboplatin, and oxaliplatin. Despite the synthesis of many other platinum compounds, their application in medicine is limited by high toxicity, unwanted side effects, immune suppression, and platinum drug resistance.^1^ Consequently, researchers are focusing on new therapeutics using nonplatinum complexes with antiproliferative effects and alternative modes of action. Several other metal complexes have shown promising cytotoxic activity, including those based on silver, which have been used in medical applications, including the development of antibacterial agents,^2^ in wound care products, medical devices, textiles, cosmetics, and even in home appliances.^3^ Although present in the human body at very low concentrations and typically bound to proteins, silver exhibits great biological compatibility and is easily eliminated from the body.^4,5^

Silver(I) complexes containing *N*-heterocyclic carbenes (NHCs) have found purpose in the field of catalysis, most notably as Lewis acid catalysts and for transmetalation applications.^6,7^ However, the success of silver in wound-healing propelled interest in this metal for other biological applications, and Ag(I)-NHCs have since been studied as potential anticancer agents.^8−10^ Ag(I)-NHC complexes have thus been evaluated against a range of cancer cell lines including pancreatic, breast, prostate, colon, cervical, liver, and others, which is extensively summarized in a recent review and references therein.^9,11^ Some complexes show enhanced selectivity towards cancer cells when compared to noncancerous cells and have been shown to induce apoptosis in the triple negative MDA-MB-231 breast cancer cell line.^12^

**Figure 1:**
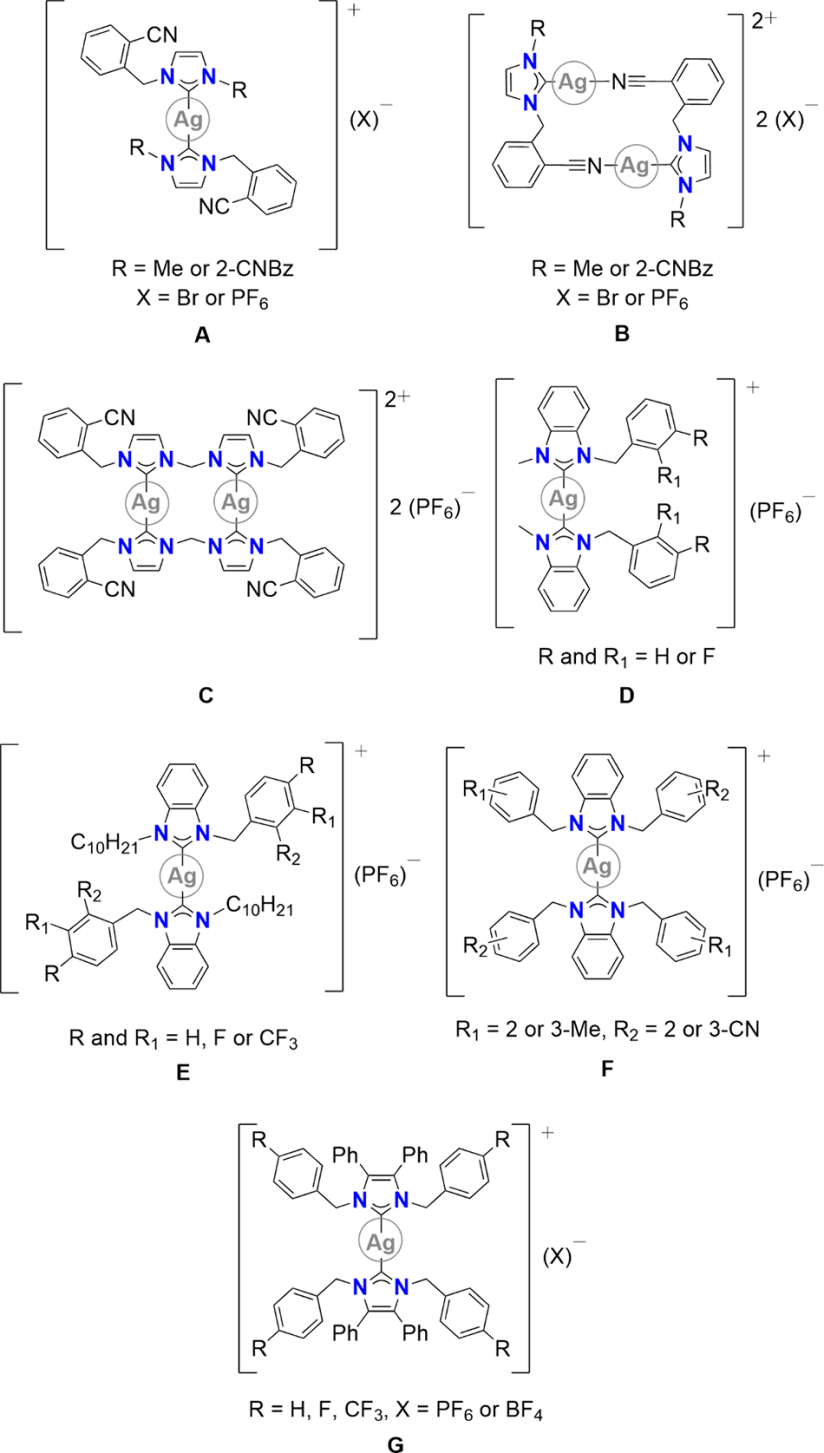
Ag(I)-NHCs which have shown bioactivity, including mononuclear (A), binuclear (B),^13^ and bis-NHC binuclear complexes (C) containing nitrile-functionalized imidazole ligands;^14^ mononuclear benzimidazole complexes containing fluorine, alkyl or nitrile-functionalized ligands (D–F); or mononuclear imidazole complexes containing fluorine-functionalized ligands (G).^14,17,18,27^

Bioactive Ag(I)-NHCs have been synthesized with a range of ligands, including, but not limited to, wingtips functionalized with nitriles,^13,14^ aliphatic chains,^15^ and bioactive molecules (e.g., clotrimazole, a fungal agent),^16^ or with a benzimidazole backbone^14,17,18^ and higher nuclearity.^19^ For example, Zetty Zulikha et al. reported the anticancer potential of mono- and binuclear nitrile-functionalized imidazole-based Ag(I)-NHC complexes and assessed their cytotoxicity toward the HCT116 colorectal cancer cell line.^13^ The binuclear Ag(I) complex (Figure 1B) is more cytotoxic than the analogous mononuclear complex (Figure 1A), as well as the free NHC ligands.^13^ Haque et al. then reported binuclear complexes of the nitrile-functionalized bis-imidazole ligands (Figure 1C) and reported the cytotoxicity against the MCF-7 breast cancer cell line.^14^ The complex was found to be 362× more active than the free NHC ligand and 4.8× more active than the well-known breast cancer drug tamoxifen.

Benzimidazole is a medically important heterocyclic scaffold in drug discovery, and benzimidazolium salts are biologically vital, having antihypertensive, anti-inflammatory, antimicrobial, and anticancer properties.^20^ Also, the addition of fluorine to drugs is well-known to enhance membrane penetration and adsorption, due to higher lipophilicity, which increases the bioavailability and increases the potency of drugs.^21−26^

Fluorine also allows for improved interaction with protein receptors and enzymes.^28^ Zin et al. reported asymmetric fluorine-substituted benzimidazole complexes (Figure 1D) and tested them against a range of cell lines.^17^ Even though the IC_50_ values are moderate for all compounds, the fluorinated complexes were less potent than their corresponding dialkylated complexes. To further probe the functional groups, Wong et al. synthesized Ag(I)-NHC complexes functionalized with both fluorine and aliphatic chain wingtips (Figure 1E) and showed that both moieties improve cancer cell cytotoxicity.^18^ Haque and co-workers have also reported Ag(I)-NHC complexes containing a benzimidazole backbone (Figure 1F), where the complexes are more active than the corresponding ligands when tested against both HCT116 colorectal cancer and*Escherichia coli* (*E. coli*).^29^ In addition, Beato et al. reported Ag(I)-NHC imidazole complexes functionalized with fluorine and highlighted their antimicrobial properties (Figure 1G).^27^ These imidazolium complexes show great potential as biological candidates which paves the way forward for the use in other pharmacological applications.^27^ In the present work, an investigation into the synthesis of fluorinated mononuclear and binuclear imidazole and benzimidazole Ag(I) complexes has been conducted, including their antiproliferative effects against breast cancer cell lines MDA-MB-231 and MCF-7, uptake by inductively coupled plasma mass spectrometry (ICP-MS), production of reaction oxygen species (ROS), and interactions with calf thymus-DNA (ct-DNA) by fluorescence spectroscopy and docking studies.

## Results and Discussion

### Synthesis and Characterization

The *para*-fluorobenzyl imidazole ligands **HL1(Br)**(30) and **H**_**2**_**L3(Br)**_**2**_(31) and benzimidazole ligand **HL2(Br)**(32) were prepared by modified literature methods. The new ligand **H**_**2**_**L4(Br)**_**2**_ was obtained from the reaction of 1-[(4-fluorophenyl)methyl]-1*H*-benzimidazole with neat dibromomethane. Successful formation of the bromide salts was easily detected by ^1^H NMR spectroscopy, due to the significant downfield shift of the (benz)imidazolium C2 proton from 7.74–8.41 to 9.31–10.48 ppm. Due to solubility differences, the anion was exchanged from Br to PF_6_ was conducted by stirring with excess ammonium hexafluorophosphate in acetone to obtain four new ligands **HL1(PF**_**6**_**)**, **HL2(PF**_**6**_**)**, **H**_**2**_**L3(PF**_**6**_**)**_**2**_, and **H**_**2**_**L4(PF**_**6**_**)**_**2**_. Successful ion exchange can be confirmed by ^19^F{^1^H} NMR spectroscopy with the appearance of a doublet at ca. −70 ppm which is characteristic of the coupling between the fluorine and the phosphorus in the PF_6_ anion. The nonfluorinated imidazole ligands **H**_**2**_**L5(Br)**_**2**_ and **H**_**2**_**L5(PF**_**6**_**)**_**2**_ were prepared by modifications of known literature methods.^33^**HL1(PF**_**6**_**)**, **H**_**2**_**L3(PF**_**6**_**)**_**2**_, and **H**_**2**_**L5(PF**_**6**_**)**_**2**_ were not required in the subsequent reactions; however, they were synthesized and characterized to provide a direct comparison between the ligands and complexes. All new ligands were fully characterized by ^1^H, ^13^C{^1^H}, and ^19^F{^1^H} NMR spectroscopy, ATR-FTIR spectroscopy, and elemental analysis.

**Scheme 1:**
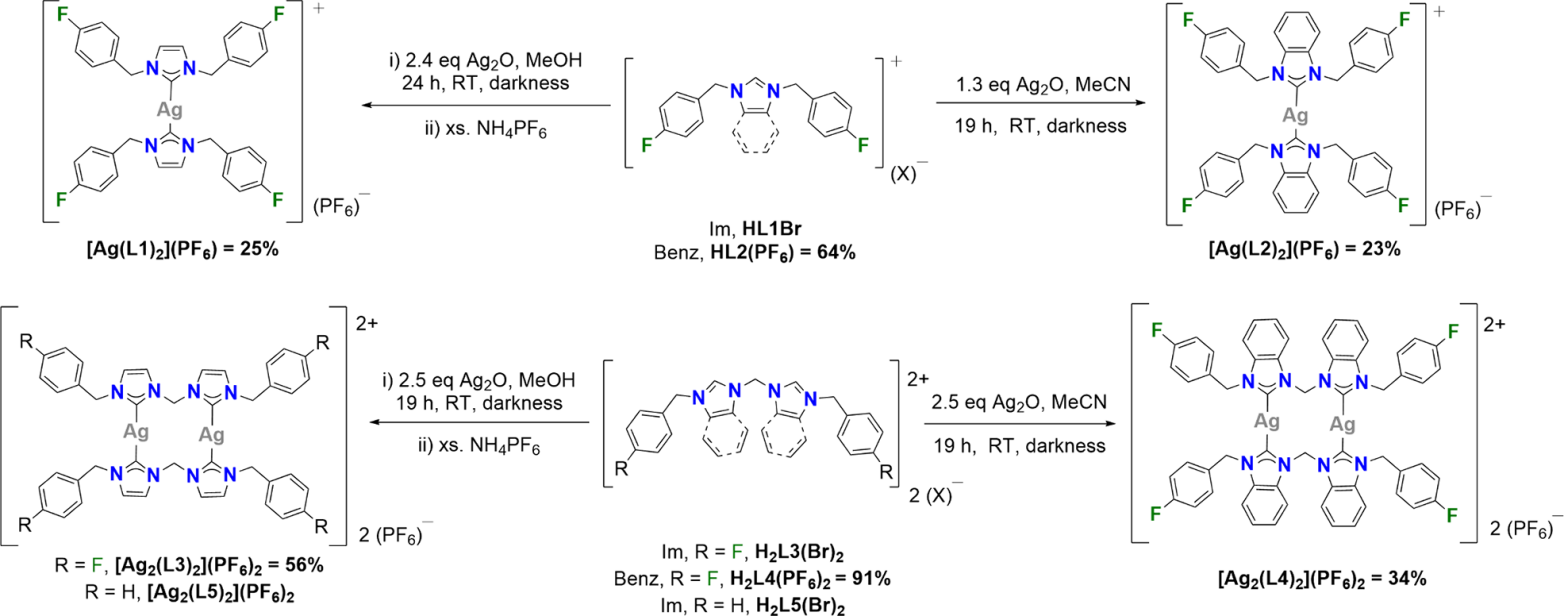
Synthetic Procedure for Mononuclear Silver Complexes **[Ag(L1)_2_](PF_6_)** and **[Ag(L2)_2_](PF_6_)** and Binuclear Complexes **[Ag_2_(L3)_2_](PF_6_)_2_**, **[Ag_2_(L4)_2_](PF_6_)_2_**, and **[Ag_2_(L5)_2_](PF_6_)_2_**

The syntheses of the corresponding Ag(I)-NHC complexes are shown in Scheme 1. Complexes **[Ag(L1)**_**2**_**](PF**_**6**_**)**, **[Ag**_**2**_**(L3)**_**2**_**](PF**_**6**_**)**_**2**_, and **[Ag**_**2**_**(L5)**_**2**_**](PF**_**6**_**)**_**2**_ were accessed from the reaction of **HL1(Br)**, **H**_**2**_**L3(Br)**_**2**_, or **H**_**2**_**L5(Br)**_**2**_ with excess silver(I) oxide in methanol in darkness for 19–24 h at room temperature, followed by filtration though Celite and the anion exchange to PF_6_.^34^ Complex **[Ag**_**2**_**(L5)**_**2**_**](PF**_**6**_**)**_**2**_ has previously been reported and successful synthesis confirmed by ^1^H NMR spectroscopy and elemental analysis only (see Supporting Information).^35^ Complexes **[Ag(L2)**_**2**_**](PF**_**6**_**)** and **[Ag**_**2**_**(L4)**_**2**_**](PF**_**6**_**)**_**2**_ were accessed via the reaction of **HL2(PF**_**6**_**)** and **H**_**2**_**L4(PF**_**6**_**)**_**2**_ with excess silver(I) oxide in acetonitrile in darkness for 19 h at room temperature. All new complexes were obtained in low to moderate yields (23–56%) and were fully characterized by ^1^H, ^13^C{^1^H}, and ^19^F{^1^H} NMR spectroscopy, ATR-FTIR spectroscopy, elemental analysis, and single crystal X-ray diffraction (scXRD) where possible.

Complex formation was confirmed by ^1^H NMR and ATR-FTIR spectroscopy. The deprotonation of the ligands is indicated by the disappearance of the (benz)imidazolium C2 proton resonance (δ 9.31–10.48 ppm), the C2–H stretch vibration at ∼3160 cm^–1^, and the C2–H in-plane bend mode between 1550 and 1579 cm^–1^. For complexes **[Ag(L2)**_**2**_**](PF**_**6**_**)**, **[Ag**_**2**_**(L3)**_**2**_**](PF**_**6**_**)**_**2**_, and **[Ag**_**2**_**(L4)**_**2**_**](PF**_**6**_**)_2_** there is further confirmation of complexation by the appearance of two sets of doublets downfield (ca. δ 190 ppm) in the ^13^C{^1^H} NMR spectra which arise from the coupling of ^107^Ag and ^109^Ag to the C2 carbon. The chemical shifts are similar to other Ag(I)-NHC complexes reported in the literature.^36^ The ion exchange for **[Ag(L1)**_**2**_**](PF**_**6**_**)** and **[Ag**_**2**_**(L3)**_**2**_**](PF**_**6**_**)**_**2**_ is seen in the ^19^F{^1^H} NMR spectra by the appearance of a doublet at ca. −70 ppm that is characteristic of the PF_6_ anion and further confirmed by the presence of a very strong and broad band in the IR spectra at ca. 830 cm^–1^. All NMR and IR spectra are shown in Figures S1–S33 and Figures S34–S38 (Supporting Information), respectively.

**Figure 2:**
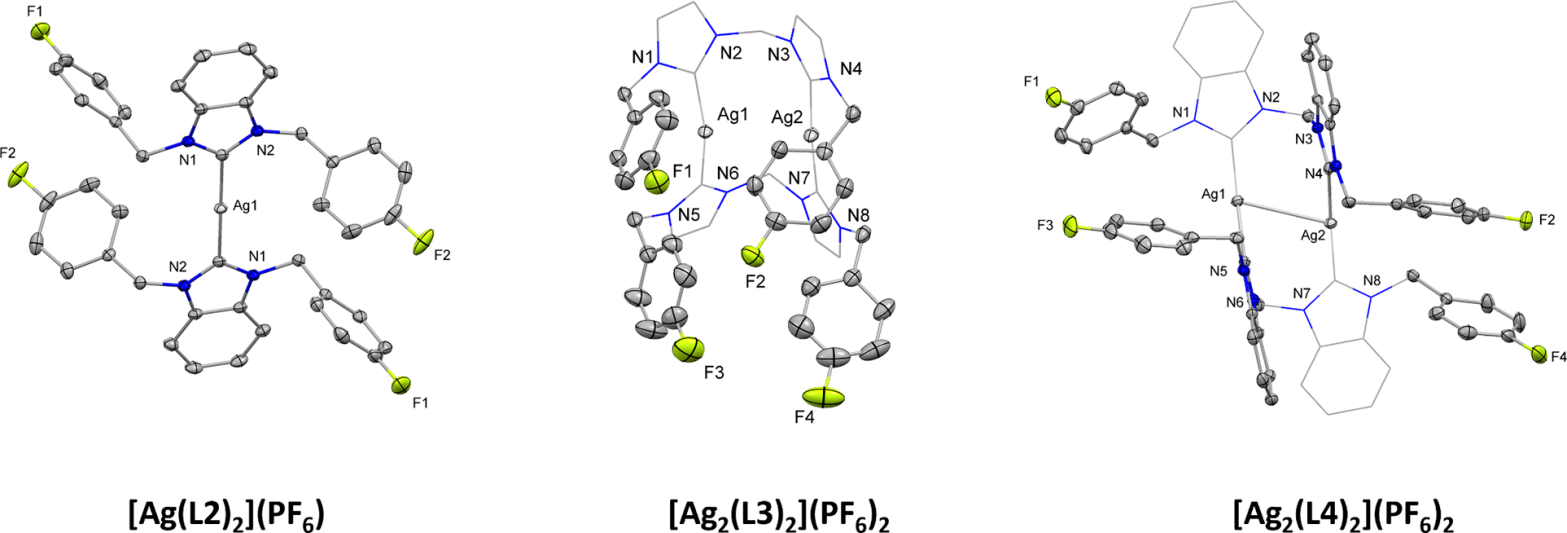
Molecular structures of **[Ag(L2)_2_](PF_6_)**, **[Ag_2_(L3)_2_](PF_6_)_2_**, and **[Ag_2_(L4)_2_](PF_6_)_2_**. All hydrogens, anions and solvent molecules are eliminated for clarity, and ellipsoids are placed at the 50% probability level.

Colorless crystals suitable for scXRD analysis were obtained for **[Ag(L2)**_**2**_**](PF**_**6**_**)**, **[Ag**_**2**_**(L3)**_**2**_**](PF**_**6**_**)**_**2**_, and **[Ag**_**2**_**(L4)**_**2**_**](PF**_**6**_**)**_**2**_ and were grown by vapor diffusion of diethyl ether into a concentrated acetonitrile solution of the complex at 2 °C. The structures were solved in a centrosymmetric *P*-1 triclinic space group for **[Ag(L2)**_**2**_**](PF**_**6**_**)** and **[Ag**_**2**_**(L3)**_**2**_**](PF**_**6**_**)**_**2**_, and in a *P*2_1_/*n* monoclinic space group for **[Ag**_**2**_**(L4)**_**2**_**](PF**_**6**_**)**_**2**_. Colorless crystals of **[Ag**_**2**_**(L4)**_**2**_**](PF**_**6**_**)**_**2**_ were also grown by evaporation of acetonitrile and gave a different complex conformation, **[Ag**_**2**_**(L4)**_**2**_**](PF**_**6**_**)**_**2**_**(2)** (Figure S40 and Table S2, Supporting Information). This structure was solved in the *P*2_1_/*c* monoclinic space group with no solvent molecules in the unit cell. The molecular structures for all complexes are shown in Figure 2, and bond angles and bond lengths stated in Table 1, which are similar to other literature examples.^37−39^ On comparing **[Ag**_**2**_**(L4)**_**2**_**](PF**_**6**_**)**_**2**_ and **[Ag**_**2**_**(L4)**_**2**_**](PF**_**6**_**)**_**2**_**(2)**, the lack of solvent molecules in the latter gives a different orientation of the pendant arms, which significantly reduces the C_carbene_–Ag–C_carbene_ bond angles (Tables 1 and S2, Supporting Information). All scXRD data and crystal packing information are stated in Table S1 and Figure S39 of the Supporting Information.

**Table 1:** Selected Bond Lengths (Å) and Bond Angles (deg) for Complexes **[Ag(L2)_2_](PF_6_)**, **[Ag_2_(L3)_2_](PF_6_)_2_**, and **[Ag_2_(L4)_2_](PF_6_)_2_**^a^

complexes	**[Ag(L2)_2_](PF_6_)**	**[Ag_2_(L3)_2_](PF_6_)_2_**	**[Ag_2_(L4)_2_](PF_6_)_2_**
bond lengths (Å)			
Ag(1)–C_carbene_	2.0984(14)/ 2.0985(14)^1^	2.096(3)/ 2.094(3)	2.098(3)/ 2.109(3)
Ag(2)–C_carbene_	n/a	2.090(3)/ 2.084(3)	2.101(3)/ 2.104(3)
N–C_carbene_ [Ag(1)]	1.3525(18)/ 1.3604(18)	1.349(4)/ 1.354(4)	1.353(4)/ 1.356(3)
		1.347(4)/ 1.365(4)	1.342(5)/ 1.361(4)
N–C_carbene_ [Ag(2)]	n/a	1.353(4)/ 1.346(4)	1.366(4)/ 1.348(4)
		1.359(4)/ 1.349(4)	1.360(3)/ 1.350(3)
Ag(1)–Ag(2)	n/a	3.2816(4)	2.9960(3)
bond angles (deg)			
C_carbene_–Ag–C_carbene_	180.0	164.80(12)/ 174.55(13)	176.45(12)/ 176.00(11)
N–C_carbene_-N [Ag(1)]	105.34(12)	104.2(3)/ 104.1(2)	105.8(3)/ 105.9(2)
N–C_carbene_-N [Ag(2)]	n/a	104.6(3)/ 104.1(3)	105.2(2)/ 106.2(2)
torsion angles (deg)			
C_carbene_-Ag(1)–Ag(2)–C_carbene_	n/a	177.85/ −177.39	–123.60/ −116.67

a s.u.s shown in parentheses.

### Chemosensitivity Studies

**Figure 3:**
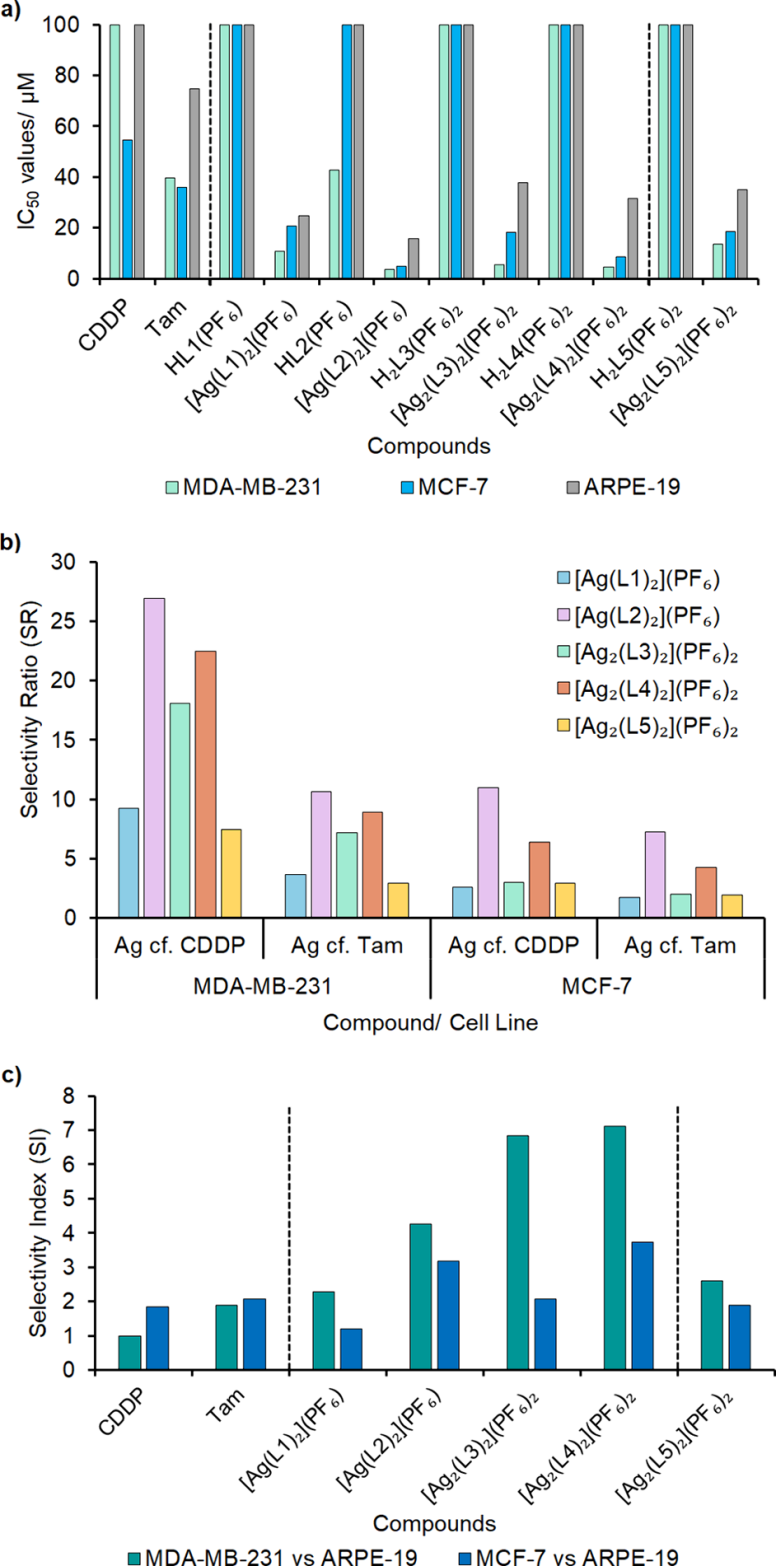
(a) Bar chart showing the IC_50_ values ± SD when cisplatin (CDDP), tamoxifen (Tam), ligands, and silver complexes were screened against breast adenocarcinomas MDA-MB-231 and MCF-7 and a noncancerous retinal epithelial cell line, ARPE-19 (*n* = 9). (b) Selectivity ratio (SR) of the Ag(I)-NHC complexes compared to both CDDP and Tam; SR > 1 shows a higher selectivity for Ag(I)-NHC. (c) Selectivity index values when comparing the cancer cell line data to the noncancerous cell line data. SI > 1 shows a selectivity for cancer cell lines over the ARPE-19 cell line.

Cell viability studies were conducted for cisplatin (CDDP), tamoxifen (Tam), ligands **HL1(PF**_**6**_**)** to **H**_**2**_**L5(PF**_**6**_**)**_**2**_, and Ag(I)-NHC complexes **[Ag(L1)**_**2**_**](PF**_**6**_**)** to **[Ag**_**2**_**(L5)**_**2**_**](PF**_**6**_**)**_**2**_ against human cell lines: breast adenocarcinomas (MDA-MB-231 and MCF-7) and noncancerous retinal epithelia (ARPE-19). All cells were treated with compounds for 24 h, and cell viability was determined via the MTT assay. The results are depicted in Figure 3a and Table S3 (Supporting Information). The ligands are generally nontoxic (IC_50_ > 100 μM) against all cell lines, except **HL2(PF**_**6**_**)**, which has a moderate IC_50_ value of 42.6 ± 0.05 μM against MDA-MB-231. The corresponding Ag(I)-NHC complexes are all cytotoxic against both the MDA-MB-231 and MCF-7 cell lines. When comparing the two fluorinated imidazole complexes **[Ag(L1)**_**2**_**](PF**_**6**_**)** and **[Ag**_**2**_**(L3)**_**2**_**](PF**_**6**_**)**_**2**_, they have similar activity against MCF-7; however, the binuclear complex **[Ag**_**2**_**(L3)**_**2**_**](PF**_**6**_**)**_**2**_ shows ca. 2-fold increase in activity against MDA-MB-231. On comparing the activities of the benzimidazole complexes **[Ag(L2)**_**2**_**](PF**_**6**_**)** and **[Ag**_**2**_**(L4)**_**2**_**](PF**_**6**_**)**_**2**_, the activities against MDA-MB-231 are not statistically different; however, the mononuclear complex **[Ag(L2)**_**2**_**](PF**_**6**_**)** is ca. 1.7-fold more active than the binuclear complex **[Ag**_**2**_**(L4)**_**2**_**](PF**_**6**_**)**_**2**_ against MCF-7 and is the most active complex in this library. Generally, all Ag(I)-NHC complexes are 9–27× and 3–11× more selective than CDDP and Tam, respectively (Figure 3b and Table S3, Supporting Information). The nonfluorinated benzyl–imidazole complex **[Ag**_**2**_**(L5)**_**2**_**](PF**_**6**_**)**_**2**_ was synthesized and screened to determine if the fluorine has any influence on cytotoxicity. When comparing the nonfluorinated complex **[Ag**_**2**_**(L5)**_**2**_**](PF**_**6**_**)**_**2**_ with the corresponding fluorinated complex **[Ag**_**2**_**(L3)**_**2**_**](PF**_**6**_**)**_**2**_, the activity against MCF-7 is statistically similar. However, on comparison of the activities against the MDA-MB-231 cell line, the addition of fluorine improves the activity by >2.4-fold, highlighting that the addition of fluorine can improve cytotoxicity and potentially selectivity toward triple negative breast cancers.

While we cannot compare the activity of our compounds directly due to researchers using different cell lines and incubation times, Wong et al. synthesized asymmetric fluorinated Ag(I)-NHC complexes (e.g., Figure 1E)^18^ and screened their activity against MCF-7 and MDA-MB-231 after 24 h incubation. Our compounds are less cytotoxic than those reported by Wong et al., whilst their *para*-fluoro Ag(I)-NHCs exhibit nanomolar potency (IC_50_ values between 0.79 ± 0.08 and 0.95 ± 0.07 μM). There are no statistical differences in the IC_50_ values against MCF-7 (like our results) or between the nonfluorinated versus fluorinated complexes against MDA-MB-231. This contrasts with our results, where against the MDA-MB-231 cell line, we observed a significant improvement in activity upon fluorination of our complexes. Wong et al. also did not observe an improvement in IC_50_ value when comparing the free NHC ligands with the Ag(I)-NHC complexes, which highlights that the cytotoxicity could be due to the release of a toxic ligand and not the silver complexes.

To address the ability of Ag(I)-NHC complexes to target cancer cell lines over noncancerous cell lines, the IC_50_ values against MDA-MB-231 and MCF-7 were compared to the IC_50_ values against ARPE-19 and are stated as selectivity index (SI) values (Figure 3c and Table S4, Supporting Information). While CDDP (IC_50_ > 100 μM), Tam (IC_50_ = 75 ± 1 μM), and the ligands (IC_50_ > 100 μM) have low to no activity against this noncancerous cell line, the Ag(I)-NHC complexes are all moderately cytotoxic. The cytotoxicity values follow the same trends as the other cell lines; however, in some cases, they are statistically less cytotoxic. This is especially true for binuclear complexes **[Ag**_**2**_**(L3)**_**2**_**](PF**_**6**_**)**_**2**_ and **[Ag**_**2**_**(L4)**_**2**_**](PF**_**6**_**)**_**2**_, where the SI values are 6.8 and 7.1, respectively (MDA-MB-231 cf. ARPE-19, Table S5, Supporting Information) and show the potential of these complexes to selectively target cancer cell lines.

The cell morphologies of MDA-MB-231 when treated with **[Ag(L2)**_**2**_**](PF**_**6**_**)** and **[Ag**_**2**_**(L4)**_**2**_**](PF**_**6**_**)**_**2**_ at 10 and 50 μM for 0, 1, and 4 h (DMSO 0.05% only was used as a control) were analyzed by optical microscopy (Figures S41–S45, Supporting Information). The initial images show cells with the same morphology as the control samples; however, after 1 h of incubation with the compounds, the cell morphology noticeably changes. They start to shrink and become rounder and detached from the plate, and by 4 h, most of the cells show signs of apoptosis.

### Cell Uptake Studies

The most active complex **[Ag**_**2**_**(L2)**_**2**_**](PF**_**6**_**)** and the most selective complex **[Ag**_**2**_**(L4)**_**2**_**](PF**_**6**_**)**_**2**_ were further investigated to establish the degree of uptake of the complex into MDA-MB-231 and MCF-7 cells. The cancer cells were grown for 24 h before being dosed with 1 μM of the complexes for 1 h. The cells were harvested, washed, and centrifuged several times to remove residual silver complex, and inductively coupled plasma mass spectrometry (ICP-MS) was used to determine metal uptake. The cells were then analyzed for ^107^Ag, and the results are displayed in Table 2.

**Table 2:** Amount of Silver Detected by ICP-MS in MDA-MB-231 and MCF-7 Cells after Incubation for 1 h with 1 μM of **[Ag(L2)_2_](PF_6_)** and **[Ag_2_(L4)_2_](PF_6_)_2_**

sample	Ag atoms/cell ± % RSD (*n* = 3)
**MDA-MB-231**	
control	not detected
**[Ag(L2)_2_](PF_6_)**	8.84 × 10^7^ ± 35
**[Ag_2_(L4)_2_](PF_6_)_2_**	8.20 × 10^7^ ± 3
MCF-7	
control	not detected
**[Ag(L2)_2_](PF_6_)**	4.91 × 10^7^ ± 3
**[Ag_2_(L4)_2_](PF_6_)_2_**	2.86 × 10^7^ ± 21

As expected, no silver was detected in the control samples for both cell lines. For complex **[Ag(L2)**_**2**_**](PF**_**6**_**)**, 8.84 × 10^7^ Ag atoms/cell were detected in the MDA-MB-231 cell line, while 4.91 × 10^7^ Ag atoms/cell were detected in the MCF-7 cell line. Although different, these values were shown not to be statistically different from each other (within % RSD), showing similar uptake in both cell lines. This was expected as the in vitro cytotoxicity data suggests comparable activity of this complex across both cell lines. For complex **[Ag**_**2**_**(L4)**_**2**_**](PF**_**6**_**)**_**2**_, the IC_50_ values suggest higher activity against MDA-MB-231. By ICP-MS, 8.20 × 10^7^ Ag atoms/cell were detected in the MDA-MB-231 cell line, while 2.86 × 10^7^ Ag atoms/cell were detected in the MCF-7 cell line. The statistically significant higher uptake of **[Ag**_**2**_**(L4)**_**2**_**](PF**_**6**_**)**_**2**_ in MDA-MD-231 than in MCF-7 points to a correlation between cytotoxicity and cellular uptake. Considering the binuclear complex **[Ag**_**2**_**(L4)**_**2**_**](PF**_**6**_**)**_**2**_ has two silver ions per complex, we should expect a higher amount of silver in the cells; however, there appears to be no difference between the amount of silver taken up in the MDA-MB-231 cell line (ca. 8.2–8.8 × 10^7^ Ag atoms/cell, and within error), suggesting that the uptake of the mononuclear complex is higher than that of its binuclear counterpart in this cell line.

### Stability Studies

Initial stability studies using **[Ag**_**2**_**(L4)**_**2**_**](PF**_**6**_**)**_**2**_ were conducted in D_2_O:DMSO 30:70 over 24 h and monitored by ^1^H NMR spectroscopy. The spectra at time points 0, 1, and 24 h are shown in Figure S46 (Supporting Information), with no noticeable changes in the resonances observed, indicating no decomposition under these conditions. However, due to the low solubility of the complex, the percentage of water could not be further increased. To address this, UV–vis spectroscopy was used to assess the stability over 24 h. Solutions of the complexes were prepared at 25 μM in H_2_O:DMSO 95:5, and the spectra are shown in Figures S47–S51 (Supporting Information). The mononuclear complex **[Ag(L1)**_**2**_**](PF**_**6**_**)** decomposes within 1 h compared to the binuclear complex **[Ag**_**2**_**(L3)**_**2**_**](PF**_**6**_**)**_**2**_, which remains relatively stable after 6 h. Complexes **[Ag(L2)**_**2**_**](PF**_**6**_**)** and **[Ag**_**2**_**(L4)**_**2**_**](PF**_**6**_**)**_**2**_ follow the same trend, with the binuclear derivative **[Ag**_**2**_**(L4)**_**2**_**](PF**_**6**_**)**_**2**_ being significantly more stable than the corresponding mononuclear complex. This trend can be partially explained by the argentophilic interaction (Ag–Ag distance <3.44 Å) between the two Ag(I) in the binuclear complexes, where similar trends have been observed in other binuclear Ag(I)-NHC complexes.^40−42^ In line with these findings, the nonfluorinated binuclear complex **[Ag**_**2**_**(L5)**_**2**_**](PF**_**6**_**)**_**2**_ is also relatively stable after 6 h, demonstrating that the incorporation of fluorine moieties does not influence the stability.

### Reactive Oxygen Species (ROS)

**Figure 4:**
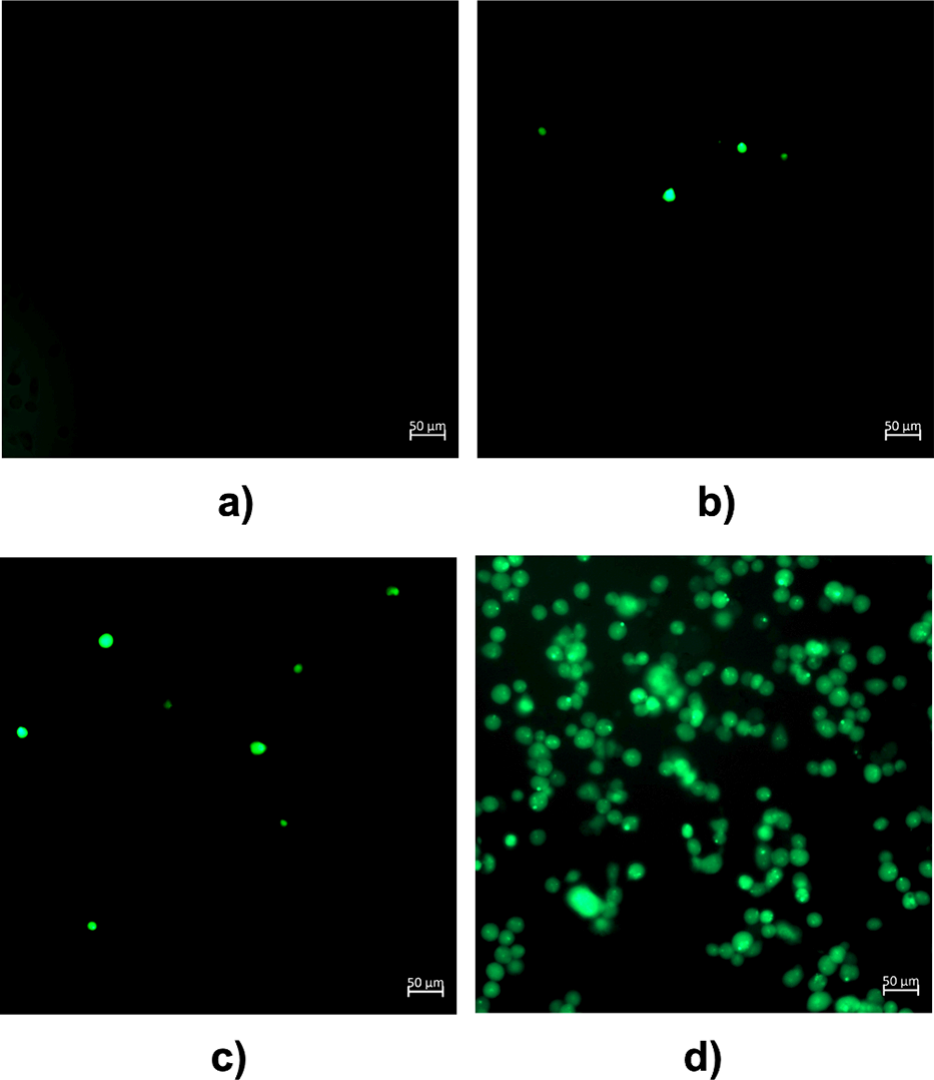
Reaction oxygen species (ROS) observed after MDA-MB-231 cells were incubated for 3.5 h with (a) control, (b) 1 × IC_50_ value, (c) 2 × IC_50_ value, and (d) 5 × IC_50_ value of **[Ag_2_(L4)_2_](PF_6_)**_**2**_ followed by H_2_DCFDA (20 μM) for 30 min. All images were taken using an Observer-7 microscope (scale bar at 50 μM) and recorded using em/ex = 494/512 nm.

The cell permeable fluorescein derivative 2′,7′-dichlorodihydrofluorescein diacetate (H_2_DCFDA) was used as an indicator for the generation of reactive oxygen species (ROS) within cells. H_2_DCFDA is deacetylated by esterase within the cell and consecutively oxidized by intracellular ROS into dichlorofluorescein (DCF) which is fluorescent.^43^ The results from fluorescence microscopy are shown in Figure 4 (Figure S52, Supporting Information). MDA-MB-231 cells were incubated with either media (control) or three different loadings the most stable complex **[Ag**_**2**_**(L4)**_**2**_**](PF**_**6**_**)**_**2**_ (1, 2, and 5 × IC_50_ concentration) for 4 h. Compared with the control, the cells treated with **[Ag**_**2**_**(L4)**_**2**_**](PF**_**6**_**)**_**2**_ show an increase in fluorescence in a dose dependent manner, with 5 × IC_50_ values exhibiting the highest levels of ROS (Figure 4d). This agrees with literature, which suggests that ROS generation by silver leads to apoptosis in cancer cells.^44^

### DNA Interactions

The interaction of **[Ag**_**2**_**(L4)**_**2**_**](PF**_**6**_**)**_**2**_ with calf thymus DNA (ct-DNA) was studied by using an ethidium bromide (EtBr) displacement assay. When EtBr is free in the Tris buffer solution, molecular oxygen quenches the fluorescence, and if the molecule intercalates between the hydrophobic pockets of DNA, the fluorescence intensity can increase by up to 20-fold.^45^ When a potential intercalator is titrated into the solution, it can displace EtBr from the DNA, thereby quenching it and lowering the fluorescence intensity. The relationship between the quencher concentration [Q] can then be related to the ratio between the initial fluorescence intensity *I*_0_ (no quencher) and the fluorescence intensity at a set concentration of quencher *I* (eq 1). These parameters are used in the Stern–Volmer equation to obtain the quenching constant, where *k*_sv_ can be extracted. In this experiment’s case, the quenching constant measures the complex’s ability to intercalate to DNA.

**Figure 5:**
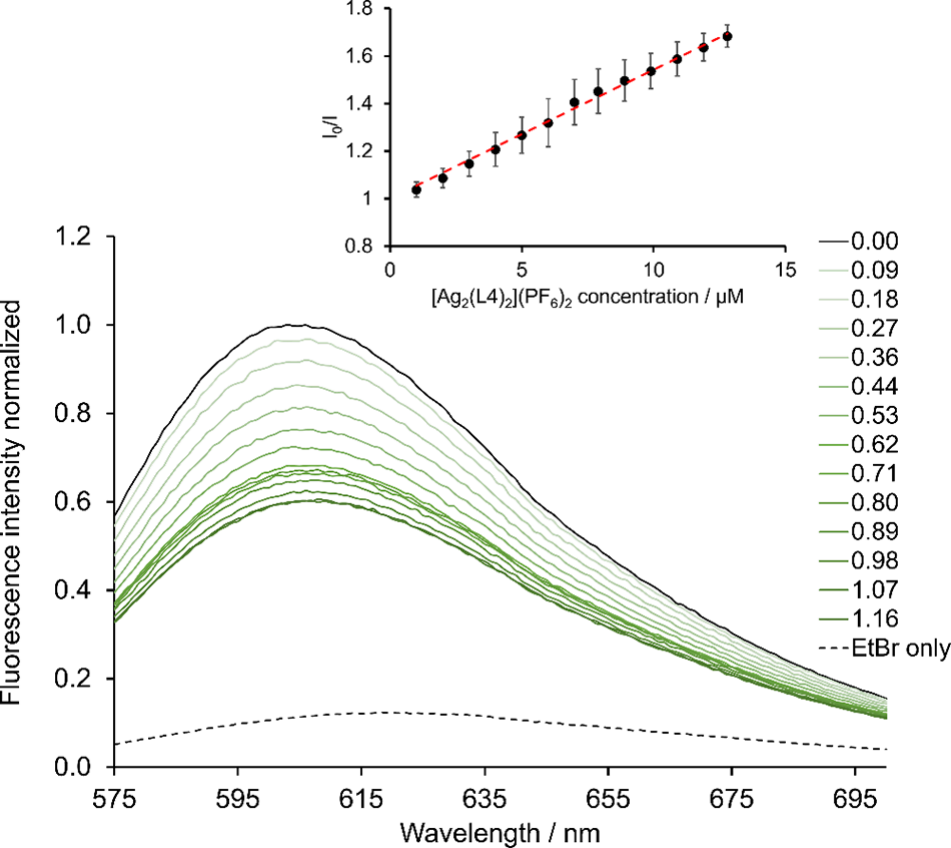
Fluorescence emission spectra of sequential additions of **[Ag_2_(L4)_2_](PF_6_)_2_** to a solution of ct-DNA/EtBr (2.5/1); black line (no complex), light–dark green (0.09–1.16 mol equiv of complex to EtBr), and dotted line (EtBr without ct-DNA). The inset shows the Stern–Volmer plot with standard errors from three independent experiments.

A titration experiment was performed by sequential addition of **[Ag**_**2**_**(L4)**_**2**_**](PF**_**6**_**)**_**2**_ to a ct-DNA/EtBr solution. The sample was excited at 545 nm (a wavelength where the complex does not absorb), and the emission spectra were recorded from 575 to 700 nm.^46^ The titration is repeated three times, and an example titration is shown in Figure 5 (triplicates are shown in Figure S53, Supporting Information). The solid black line shows the initial fluorescence spectrum of ct-DNA and EtBr, and the dotted black line shows EtBr only. The lines from light to dark green show sequential addition of **[Ag**_**2**_**(L4)**_**2**_**](PF**_**6**_**)**_**2**_ (from 0.09–1.16 molar ratio of complex to EtBr). The fluorescence intensity decreases upon increasing concentration of complex, which is indicative of displacement of EtBr and intercalation of **[Ag**_**2**_**(L4)**_**2**_**](PF**_**6**_**)**_**2**_. The samples in the absence and presence of complex were calculated and plotted against concentration to obtain a Stern–Volmer plot, where the gradient equals the binding constant, *k*_sv_. This was calculated as 5.4 ± 0.8 × 10^4^ M^–1^, which is stronger than the chemotherapeutic intercalator doxorubicin (2.33 ± 0.33 × 10^4^ M^–1^).^47^ Sanchez et al. report a binuclear methyl-functionalized imidazole-based silver NHC complex which had a reported binding constant of 0.29 × 10^4^ M^–1^ which is significantly lower than that of **[Ag**_**2**_**(L4)**_**2**_**](PF**_**6**_**)**_**2**_ indicating that the aromatic wingtips and extended backbone increase the ability for Ag(I)-NHC complexes to intercalate, rendering it an effective B-DNA intercalator.^48^
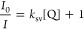
1

### Molecular Docking Studies

**Figure 6:**
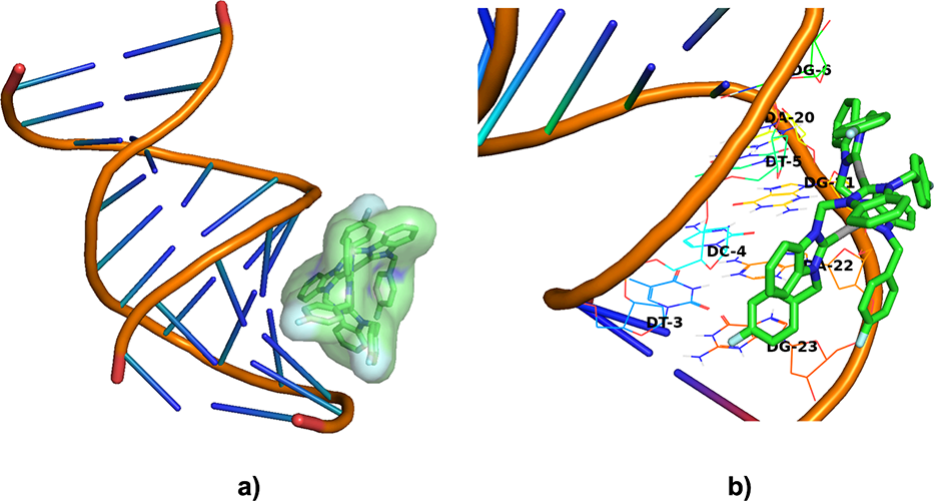
Binding mode between **[Ag_2_(L4)_2_](PF_6_)_2_** and DNA: (a) surface representation and (b) with specific residues.

DNA is a common biological target of many metal-based complexes, including the metallodrug cisplatin. Since **[Ag**_**2**_**(L4)**_**2**_**](PF**_**6**_**)**_**2**_ was shown to bind DNA in biophysical studies, in silico docking was used to elucidate potential binding modes with DNA. A blind docking approach was used to investigate this interaction using AutoDock 4.2 making use of the crystal structure of a DNA dodecamer obtained from the protein data bank (PDB: 1LU5). Docking studies are often performed to elucidate a proposed binding mode between ligand and biomolecule, where the DNA dodecamer used here is a common model for B-DNA.^49^ The docking studies were performed using the structure of **[Ag**_**2**_**(L4)**_**2**_**](PF**_**6**_**)**_**2**_ obtained from Density Functional Theory (DFT) optimization calculations. The complex was docked at least 3 times to obtain information about the binding interaction and to check reproducibility. The lowest energy conformation (estimated free energy of binding = −7.93 kcal·mol^–1^) is shown in Figure 6. The complex displayed nonpolar interactions between the **[Ag**_**2**_**(L4)**_**2**_**](PF**_**6**_**)**_**2**_ and DNA residues DT-3, DC-4, DG-6, DT-5, DA-20, DG-21, DA-22, and DG-23 in the major groove, with close contacts (<3 Å) observed between DG-6 and the benzimidazole ring, as well as DG-20 and the benzimidazole moiety; DT-3, DG-23, and DA-22 and the fluorobenzene moiety; and DG-21 and the wingtips of the complex. The docking study suggests the complex interacts by means of van der Waals (vdW) interactions with clashes (vdW distance ratios <0.89) shown in Figure S54 (Supporting Information).

## Conclusion

The syntheses of five new (benz)imidazolium NHC ligands and four new Ag(I)-NHC complexes are reported. All complexes were obtained by reacting the respective ligands with an excess of silver(I) oxide followed by an ion exchange if necessary. The complexes were obtained in low to moderate yields and fully characterized, including scXRD for three new complexes.

The complexes stability in aqueous media was assessed, showing that the binuclear complexes were more stable than their mononuclear counterparts. The cytotoxicity of all ligands and complexes were assessed against three cell lines (MDA-MB-231, MCF-7, and ARPE-19), and all silver complexes are significantly more active than the corresponding ligand. Upon comparison of the fluorinated complex with a nonfluorinated analogue, the activity against MDA-MD-231 improved by >2.4× upon the addition of fluorine.

The complexes containing a benzimidazole core perform overall better than the mononuclear complexes, with **[Ag(L2)**_**2**_**](PF**_**6**_**)** being the most active against MDA-MB-231 with an IC_50_ values of 3.7 ± 0.3 μM, and the binuclear complex **[Ag**_**2**_**(L4)**_**2**_**](PF**_**6**_**)**_**2**_ being the most selective (SI = 7.1). Cell uptake studies of **[Ag(L2)**_**2**_**](PF**_**6**_**)** and **[Ag**_**2**_**(L4)**_**2**_**](PF**_**6**_**)**_**2**_ suggest a correlation between the cellular uptake and activity.

The possible mechanism of action of **[Ag**_**2**_**(L4)**_**2**_**](PF**_**6**_**)**_**2**_ was further investigated using a reactive oxygen species (ROS) assay, intercalation studies using ethidium bromide, and molecular docking studies, which all show that this complex generates ROS in a dose-dependent manner, can intercalate with DNA and that the complex can interact with the major groove of DNA via vdW interactions.

## Experimental Section

### General Information

Chemicals were purchased from Sigma-Aldrich (Merck KGaA), Fisher Scientific, and Fluorochem and used without further purification. ^1^H, ^13^C, and ^19^F NMRs are recorded on either a Bruker Avance III 400 (Ultrashield 400 Plus) or a Bruker Avance III 500 (Ascent 500) and referenced to TMS using the respective residual solvent signal as a secondary standard. The spectra are processed in MestReNova 14.0.1, and multiplicities are abbreviated as s = singlet, d = doublet, t = triplet, q = quartet, br = broad, m = multiplet, or respective combinations. UV–vis spectra were collected on a JASCO 730 UV–visible Spectrophotometer equipped with a PAC-743R temperature control unit. IR spectra were collected on a PerkinElmer Spectrum Two FT-IR Spectrometer (UATR Two Probe). Fluorescence spectra were collected on an Edinburgh FS5 Spectrofluorometer. Fluorescence imaging was taken on a Zeiss Observer 7 microscope, and the images were processed using Zeiss Zen 3.8 imaging software. Optical cell images were taken on a ZEISS Primo Vert microscope at 10× magnification with a GXCAM digital camera mounted and processed in the GT Vision GXCapture-T software.

### Synthesis and Characterization

Compounds **HL1(Br)**,^30^**HL2(Br)**,^32^**H**_**2**_**L3(Br)**_**2**_,^31^**H**_**2**_**L5(Br)**_**2**_**, H**_**2**_**L5(PF**_**6**_**)**_**2**_,^33^ and **[Ag**_**2**_**(L5)**_**2**_**](PF**_**6**_**)**_**2**_,^35^ have been synthesized via literature methods, and successful synthesis was confirmed by ^1^H NMR spectroscopy and elemental analysis (see Supporting Information). Ligands **HL1(PF**_**6**_**)** (±0.62), **H**_**2**_**L3(PF**_**6**_**)**_**2**_ (±0.47), and **H**_**2**_**L4(PF**_**6**_**)**_**2**_ (±0.78) and complexes **[Ag(L2)**_**2**_**](PF**_**6**_**)** (±0.56), **[Ag**_**2**_**(L3)**_**2**_**](PF**_**6**_**)**_**2**_ (±0.53), and **[Ag**_**2**_**(L5)**_**2**_**](PF**_**6**_**)**_**2**_ (±0.54, Supporting Information) have elemental analysis values which are outside the required ±0.4%. Although these results are outside the range viewed as establishing analytical purity, they are provided to illustrate the best values obtained to date. The lower than expected hydrogen values can be explained by the lack of composition aid in the analysis of the samples. To support the results, we have also provided ^1^H, ^13^C{^1^H}, ^19^F{^1^H} NMR, FT-IR, and single crystal X-ray diffraction where possible, to prove products were achieved successfully.

### Ligand 1 = **HL1(PF_6_)**

1-[(4-Fluorophenyl)methyl]-1*H*-imidazole (1.00 g, 5.68 mmol) and 4-fluorobenzyl bromide (1.19 g, 6.27 mmol) were stirred in acetonitrile (10 mL) and heated to reflux for 24 h. The solvent was removed in vacuo to yield a brown oil. Acetone (25 mL) was added followed by ammonium hexafluorophosphate (1.16 g, 7.10 mmol), and the mixture was stirred for 1 day. The solvent was removed, and the resulting colorless powder was suspended in water (40 mL) and stirred for 1 h. The powder was collected and washed with deionized water (2 × 40 mL) and diethyl ether (3 × 40 mL) and dried in vacuo yielding a colorless powder. Yield: 0.86 g, 2.00 mmol, 35%; ^1^H NMR (500 MHz, (CD_3_)_2_SO, 298 K) δ: 9.31 (t, 1H, ^4^*J*(^1^H–^1^H) = 1.6 Hz), 7.80 (d, 2H, ^4^*J*(^1^H–^1^H) = 1.6 Hz), 7.53–7.48 (m, 4H), 7.30–7.25 (m, 4H), 5.40 (s, 4H); ^13^C{^1^H} NMR (126 MHz, (CD_3_)_2_SO, 298 K) δ: 162.3 (d, ^1^*J*(^13^C–^19^F) = 245.3 Hz, Q CF), 136.2 (CH), 130.9 (d, ^3^*J*(^13^C–^19^F) = 8.8 Hz, CH), 130.9 (Q), 122.9 (CH), 115.9 (d, ^4^*J*(^13^C–^19^F) = 21.4 Hz, CH), 51.3 (CH_2_); ^19^F{^1^H} NMR (471 MHz, (CD_3_)_2_SO, 298 K) δ: −70.1 (d, ^1^*J*(^19^F–^31^P) = 710.6 Hz, PF_6_), −113.0 (CF); IR (ATR, cm^–1^): υ̃ = 3177 (w, C4,5-H), 3160 (w, C2–H), 1606 (m, aromatic C–C), 1569 (m), 1561 (sh, C2–H ip bend), 1510 (s, aromatic C–C), 824 (vs, br, PF_6_); Elemental Analysis Calculated for C_17_H_15_F_8_N_2_P: C, 47.45; H, 3.51; N 6.51%; Analysis Found: C, 47.28; H, 3.27; N 5.89%.

### Ligand 2 = **HL2(PF_6_)**

1,3-Di(4-fluorobenzyl)benzimidazolium bromide (0.62 g, 1.49 mmol) (see Supporting Information for synthesis) was stirred in acetone (50 mL) with ammonium hexafluorophosphate (0.31 g, 1.87 mmol) for 1 h. The suspension was filtered, and the solvent was removed, yielding a colorless powder. Yield: 0.41 g, 0.95 mmol, 64%; ^1^H NMR (500 MHz, (CD_3_)_2_SO, 298 K) δ: 9.92 (s, 1H), 7.97 (dd, 2H, ^3^*J*(^1^H–^1^H) = 6.4 Hz, ^4^*J*(^1^H–^1^H) = 3.2 Hz), 7.65 (dd, 2H, ^3^*J*(^1^H–^1^H) = 6.4 Hz, ^4^*J*(^1^H–^1^H) = 3.2 Hz), 7.63–7.58 (m, 4H), 7.32–7.25 (m, 4H), 5.76 (s, 4H); ^13^C{^1^H} NMR (125 MHz, (CD_3_)_2_SO, 298 K) δ: 162.3 (d, ^1^*J*(^13^C–^19^F) = 245.5 Hz, Q CF), 142.7 (CH), 131.1 (Q), 130.9 (d, ^3^*J*(^13^C–^19^F) = 9.1 Hz, CH), 130.0 (d, ^4^*J*(^13^C–^19^F) = 3.0 Hz, Q), 127.3 (CH), 115.9 (d, ^2^*J*(^13^C–^19^F) = 21.1 Hz, CH), 114.0 (CH), 49.3 (CH_2_); ^19^F{^1^H} NMR (470 MHz, (CD_3_)_2_SO, 298 K) δ: −70.1 (d, ^1^*J*(^19^F–^31^P) = 715.3 Hz, PF_6_), −114.2 (CF); IR (ATR, cm^–1^): υ̃ = 3153 (w, C2–H), 1606 (m, aromatic C–C), 1563 (m, C2–H ip bend), 1510 (s, aromatic C–C), 817 (vs, br, PF_6_); Elemental Analysis Calculated for C_21_H_17_F_8_N_2_P: C, 52.51; H, 3.57; N 5.83%; Analysis Found: C, 52.93; H, 3.60; N 5.85%.

### Ligand 3 = **H_2_L3(PF_6_)_2_**

1-[(4-Fluorophenyl)methyl]-1*H*-imidazole (1.00 g, 5.70 mmol) was dissolved in dibromomethane (2 mL) to form an orange solution and heated to 80 °C for 1 day. A precipitate formed, and the mixture was allowed to cool to room temperature. The precipitate was collected by vacuum filtration and washed with diethyl ether (3 × 40 mL) resulting in a colorless powder. The powder was suspended in acetone (50 mL), after which ammonium hexafluorophosphate (1.15 g, 7.07 mmol) was added. The mixture was stirred for 1 day, and the solvent was removed under reduced pressure. The resulting solid was stirred in deionized water (50 mL) for 1 h for 30 min, collected by vacuum filtration, and washed with deionized water (2 × 40 mL) and diethyl ether (3·40 mL) and dried in vacuo yielding a colorless solid. Yield: 1.30 g, 1.98 mmol, 69%; ^1^H NMR (400 MHz, (CD_3_)_2_SO, 298 K) δ: 9.41 (t, 2H ^4^*J*(^1^H–^1^H) = 1.8 Hz), 7.97 (dd, 2H, ^3^*J*(^1^H–^1^H) = 2.1 Hz, ^4^*J*(^1^H–^1^H) = 1.8 Hz), 7.87 (dd, 2H, ^3^*J*(^1^H–^1^H) = 2.1 Hz, ^4^*J*(^1^H–^1^H) = 1.8 Hz), 7.55–7.49 (m, 4H), 7.33–7.29 (m, 4H), 6.57 (s, 2H), 5.46 (s, 4H); ^13^C{^1^H} NMR (100 MHz, (CD_3_)_2_SO, 298 K) δ: 162.4 (d, ^1^*J*(^13^C–^19^F) = 246 Hz, Q CF), 137.7 (CH), 131.2 (d, ^3^*J*(^13^C–^19^F) = 8.5 Hz, CH), 130.4 (d, ^4^*J*(^13^C–^19^F) = 3.2 Hz, Q), 123.2 (CH), 122.7 (CH), 116.0 (d, ^2^*J*(^13^C–^19^F) = 20.2 Hz, CH), 58.6 (CH_2_), 51.60 (CH_2_); ^19^F{^1^H} NMR (470 MHz, (CD_3_)_2_SO, 298 K) δ: −70.9 (d, ^1^*J*(^19^F–^31^P) = 710.6 Hz, (PF_6_), −112.8 (CF); IR (ATR, cm^–1^): υ̃ = 3188 (w, C4,5–H), 3168 (w, C2–H), 1613 (sh), 1605 (m, aromatic C–C), 1550 (m), 1561 (sh, C2–H ip bend), 1515 (s, aromatic C–C), 825 (vs, br, PF_6_); Elemental Analysis Calculated for C_21_H_20_F_14_N_4_P_2_: C, 38.43; H, 3.07; N 8.54%; Analysis Found: C, 38.59; H, 2.69; N 8.07%.

### Ligand 4 = **H_2_L4(Br)_2_**

1-[(4-Fluorophenyl)methyl]-1*H*-benzimidazole (1.50 g, 6.63 mmol) was dissolved in dibromomethane (10 mL) and heated to reflux for 24 h. The mixture was allowed to cool, and the solvent was removed under reduced pressure. The resulting yellow solid was washed with acetone (40 mL), collected by filtration, and washed with acetone (4 × 10 mL) and diethyl ether (4 × 10 mL) and dried in vacuo resulting in a colorless powder. Yield: 1.58 g, 2.52 mmol, 76%; ^1^H NMR (400 MHz, (CD_3_)_2_SO, 298 K) δ: 10.48 (s, 2H), 8.39 (d, 2H, ^3^*J*(^1^H–^1^H) = 8.4 Hz), 8.04 (d, 2H, ^3^*J*(^1^H–^1^H) = 8.4 Hz), 7.79 (dt, 2H, ^3^*J*(^1^H–^1^H) = 8.4, Hz, ^4^*J*(^1^H–^1^H) = 0.8 Hz), 7.72 (dt, 2H, ^3^*J*(^1^H–^1^H) = 7.8, Hz, ^4^*J*(^1^H–^1^H) = 0.8 Hz), 7.68–7.61 (m, 4H), 7.43 (s, 2H), 7.33–7.26 (m, 4H), 5.85 (s, 4H); ^13^C{^1^H} NMR (125 MHz, (CD_3_)_2_SO, 298 K) δ: 162.3 (d, ^1^*J*(^13^C–^19^F) = 245.3 Hz, Q CF), 144.2 (CH), 131.1 (d, ^3^*J*(^13^C–^19^F) = 8.8 Hz, CH), 130.7 (CH), 130.7 (CH), 129.7 (d, ^4^*J*(^13^C–^19^F) = 3.8 Hz, Q), 127.6 (CH), 127.3 (CH), 115.9 (d, ^2^*J*(^13^C–^19^F) = 21.4 Hz, CH), 114.3 (CH), 114.0 (CH), 55.4 (CH), 49.5 (CH_2_); ^19^F{^1^H} NMR (470 MHz, (CD_3_)_2_SO, 298 K) δ: −112.8 (CF); IR (ATR, cm^–1^): υ̃ = 3460 (s, as(H_2_O)), 3402 (m, s(H_2_O)), 3125 (w), 3109 (sh, C–H benz), 2992 (m, br, C2–H), 1606 (w, aromatic C–C), 1556 (s, C2–H ip bend), 1510 (s, aromatic C–C); Analysis Calculated for C_29_H_24_Br_2_F_2_N_4_·H_2_O: C, 54.06; H, 4.07; N 8.70%; Analysis Found: C, 54.33; H, 3.51; N 8.12%.

### **H_2_L4(PF_6_)_2_**

1,1′-Bis(4-fluorobenzyl)-3,3′-methylenedibenzimidazolium dibromide (1.50 g, 2.39 mmol) was suspended in acetone (50 mL) after which ammonium hexafluorophosphate (0.98 g, 6.01 mmol) was then added. The mixture was stirred for 1 day. The resulting suspension was filtered, and the solvent was removed under reduced pressure. The resulting solid was stirred in deionized water (50 mL) for 1 h. The solid was collected by vacuum filtration, washed with deionized water (2·40 mL) and diethyl ether (3·40 mL), and dried in vacuo, yielding a colorless solid. Yield: 1.65 g, 2.18 mmol, 91%; ^1^H NMR (400 MHz, (CD_3_)_2_SO, 298 K) δ: 10.31 (s, 2H), 8.35 (d, 2H, ^3^*J*(^1^H–^1^H) = 8.4 Hz), 8.05 (d, 2H, ^3^*J*(^1^H–^1^H) = 8.4 Hz), 7.80 (td, 2H, ^3^*J*(^1^H–^1^H) = 7.6 Hz, ^4^*J*(^1^H–^1^H) = 1.0 Hz), 7.73 (td, 2H, ^3^*J*(^1^H–^1^H) = 7.6 Hz, ^4^*J*(^1^H–^1^H) = 1.0 Hz), 7.63–7.56 (m, 4H), 7.35 (s, 2H), 7.33–7.27 (m, 4H), 5.81 (s, 4H); ^13^C{^1^H} NMR (100 MHz, (CD_3_)_2_SO, 298 K) δ: 162.3 (d, ^1^*J*(^13^C–^19^F) = 245.5 Hz, Q CF), 144.1 (CH), 130.9 (d, ^3^*J*(^13^C–^19^F) = 8.0 Hz, CH), 130.8 (CH), 129.6 (d, ^4^*J*(^13^C–^19^F) = 3.0 Hz, Q), 127.6 (CH), 127.4 (CH), 116.0 (d, ^2^*J*(^13^C–^19^F) = 22.1 Hz, CH), 114.3 (CH), 113.8 (CH), 55.3 (CH_2_), 49.6 (CH_2_); ^19^F{^**1**^H} NMR (470 MHz, (CD_3_)_2_SO, 298 K) δ: −70.1 (d, ^1^*J*(^19^F–^31^P) = 710.6 Hz, PF_6_) −112.7 (CF); IR (ATR, cm^–1^): υ̃ = 3165 (w), 3147 (w, C2–H), 3109 (m, _C_-H benz), 1613 (w), 1602 (w, aromatic C–C), 1579 (m), 1571 (, C2–H ip bend), 1516 (s, aromatic C–C), 829 (vs, br, PF_6_); Analysis Calculated for C_29_H_24_F_14_N_4_P_2_: C, 46.05; H, 3.20; N 7.41%; Analysis Found: C, 45.79; H, 2.41; N 7.34%.

### **[Ag(L1)_2_](PF_6_)**

1,3-Di(4-fluorobenzyl)imidazolium bromide (0.20 g, 0.55 mmol) and silver(I) oxide (0.16 g, 1.30 mmol) were stirred in methanol (10 mL) under the exclusion of light for 24 h. The brownish suspension was passed through Celite, and the plug was washed with methanol (20 mL), giving a light-yellow solution. The solution was added to a stirred solution of ammonium hexafluorophosphate (0.095 g, 0.58 mmol) in deionized water (70 mL), to form an oily yellowish solid. The supernatant was decanted, diethyl ether (20 mL) was added to the oil, and the mixture was triturated to give a loose powder. The powder was collected and dried in vacuo yielding a colorless powder. Yield: 0.057 g. 0.069 mmol, 25%; ^1^H NMR (400 MHz, (CD_3_)_2_SO, 298 K) δ: 7.58 (s, 4H), 7.25–7.19 (m, 8H), 7.14–7.07 (m, 8H), 5.29 (s, 8H); ^13^C{^1^H} NMR (100 MHz, (CD_**3**_)_2_SO, 298 K) δ: 161.7 (d, ^1^*J*(^13^C–^19^F) = 244.5 Hz, Q CF), 133.4 (d, ^4^*J*(^13^C–^19^F) = 3.0 Hz, Q), 129.4 (d, ^2^*J*(^13^C–^19^F) = 8.0 Hz, CH), 122.8 (CH), 115.6 (d, ^2^*J*(^13^C–^19^F) = 21.1 Hz, CH), 53.4 (CH_2_); ^19^F{^1^H} NMR (470 MHz, (CD_3_)_2_SO, 298 K) δ: −70.1 (d, ^1^*J*(^19^F–^31^P) = 710.6 Hz, PF_6_), 114.2 (CF); IR (ATR, cm^–1^): υ̃ = 3177 (w, C4,5-H), 1604 (m, aromatic C–C), 1509 (s, aromatic C–C), 830 (vs, br, PF_6_); Elemental Analysis Calculated for C_34_H_28_AgF_10_N_4_P: C, 49.71; H, 3.44; N 6.82%; Analysis Found: C, 49.24; H, 3.68; N 6.33%.

### **[Ag(L2)_2_](PF_6_)**

1,3-Di(4-fluorobenzyl)imidazolium hexafluorophosphate (0.20 g, 0.42 mmol) was stirred with silver(I) oxide (0.12 g, 0.53 mmol) in acetonitrile (10 mL) under the exclusion of light for 19 h. The brown suspension was passed through Celite and washed with acetonitrile (50 mL). The solvent was removed under reduced pressure to give a waxy, colorless solid. The solid was triturated with methanol (10 mL) forming a loose powder, which was collected by vacuum filtration and washed with methanol (10 mL) and *n*-heptane (20 mL) and then dried in vacuo to give a colorless powder. Yield: 0.05 g, 0.047 mmol, 23%; ^1^H NMR (400 MHz, (CD_3_)_2_SO, 298 K) δ: 7.75 (dd, 4H, ^3^*J*(^1^H–^1^H) = 6.4 Hz, ^4^*J*(^1^H–^1^H) = 3.0 Hz), 7.42 (dd, 4H, ^3^*J*(^1^H–^1^H) = 6.4 Hz, ^4^*J*(^1^H–^1^H) = 3.0 Hz), 7.35–7.29 (m, 8H), 7.11–7.04 (m, 8H), 5.75 (s, 8H); ^13^C{^1^H} NMR (125 MHz, (CD_3_)_**2**_SO, 298 K) δ: 189.7 (d, ^1^*J*(^13^C–^107^Ag) = 182.6 Hz and ^1^*J*(^13^C–^109^Ag) = 210.3 Hz, CAg), 161.7 (d, ^1^J(^13^C–^19^F) = 245.3 Hz, Q CF), 133.4 (Q), 132.5 (d, ^4^*J*(^13^C–^19^F) = 2.5 Hz, Q), 129.2 (d, ^3^*J*(^13^C–^19^F) = 7.5 Hz, CH), 124.5 (CH), 115.6 (d, ^2^*J*(^13^C–^19^F) = 21.4 Hz, CH), 112.5 (CH), 51.0 (CH_2_); ^19^F{^1^H} NMR (470 MHz, (CD_3_)_2_SO, 298 K) δ: −70.1 (d, ^1^*J*(^19^F–^31^P) = 715.3 Hz, PF_6_), −114.2 (CF); IR (ATR, cm^–1^): υ̃ = 1604 (m, aromatic C–C), 1509 (s, aromatic C–C), 824 (vs, br, PF_6_); Elemental Analysis Calculated for C_42_H_32_AgF_10_N_4_P: C, 54.74; H, 3.50; N 6.08%; Analysis Found: C, 54.81; H, 2.98; N 6.03%.

### **[Ag_2_(L3)_2_](PF_6_)_2_**

1,1′-Bis(4-fluorobenzyl)-3,3′-methylenediimidazolium dibromide (0.20 g, 0.38 mmol) and silver(I) oxide (0.22 g, 0.95 mmol) were stirred in methanol (10 mL) under the exclusion of light for 19 h. The resulting brown suspension was passed through Celite giving a light-yellow solution, and methanol (20 mL) was added. Ammonium hexafluorophosphate (0.13 g, 0.80 mmol) in deionized water (60 mL) was added to form a suspension, which was stirred for 1 h. The colorless precipitate was collected by vacuum filtration, washed with deionized water (3 × 40 mL) and diethyl ether (3 × 40 mL), and dried in vacuo giving a colorless powder. Yield: 0.13 g, 0.11 mmol, 56%; ^1^H NMR (500 MHz, (CD_**3**_**)**_**2**_SO, 298 K) δ: 7.91 (d, 4H, ^3^*J*(^1^H–^1^H) = 1.8 Hz), 7.60 (d, 4H, ^3^*J*(^1^H–^1^H) = 1.8 Hz), 7.15–7.11 (m, 8H), 7.09–7.04 (m, 8H), 7.02–6.22 (br. m, 4H), 5.23 (s, 8H); ^**13**^C{^1^H} NMR (125 MHz, (CD_3_)_2_SO, 298 K) δ: 181.0 (d, ^1^*J*(^13^C-^107^Ag) = 183.7 Hz and ^1^*J*(^13^C–^109^Ag) = 210.1 Hz, CAg), 161.7 (d, ^1^*J*(^13^C–^19^F) = 244.7 Hz, CF), 132.8 (d, ^4^*J*(^13^C–^19^F) = 2.5 Hz, Q), 129.2 (d, ^3^*J*(^13^C–^19^F) = 8.3 Hz, CH), 123.5 (CH), 122.5 (CH), 115.6 (d, ^2^*J*(^13^C–^19^F) = 21.7 Hz, CH), 63.6 (CH_2_), 53.6 (CH_2_); ^19^F{^1^H} NMR (470 MHz, (CD_3_**)**_2_SO, 298 K) δ: −70.1 (d, ^1^*J*(^19^F–^31^P) = 710.6 Hz, PF_6_), −114.1 (CF); IR (ATR, cm^–1^): υ̃ = 3176 (w, C4,5-H), 1606 (m, aromatic C–C), 1573 (w, imid C–C), 1511 (s, aromatic C–C), 833 (vs, br, PF_6_); Elemental Analysis Calculated for C_42_H_36_Ag_2_F_16_N_8_P_2_: C, 40.86; H, 2.94; N 9.08%; Analysis Found: C, 40.96; H, 2.41; N 8.69%.

### **[Ag_2_(L4)_2_](PF_6_)_2_**

1,1′-Bis(4-fluorobenzyl)-3,3′-methylenedibenzimidazolium dihexafluorophosphate (1.50 g, 1.98 mmol) and silver(I) oxide (1.15 g, 4.96 mmol) were stirred in acetonitrile (6 mL) under the exclusion of light for 19 h. The mixture was passed through a Celite plug, and the solvent was removed from the filtrate giving a brown powder. The powder was dissolved in acetone (20 mL) and passed through a Celite. The solvent was removed from the filtrate giving a yellow oil, which was redissolved in acetone (5 mL) and methanol (20 mL). Water (60 mL) was added, and a colorless precipitate formed, which was collected by vacuum filtration, washed with methanol (5 mL) and petroleum ether (5 mL), and dried in vacuo, yielding a colorless solid. Yield: 0.49 g, 0.34 mmol, 34%; ^**1**^H NMR (400 MHz, (CD_3_)_2_SO, 298 K) δ: 8.12 (d, 4H, ^3^*J*(^1^H–^1^H) = 8.2 Hz), 7.80, (d, 4H, ^3^*J*(^1^H–^1^H) = 8.2 Hz), 7.60 (t, 4H, ^3^*J*(^1^H–^1^H) = 7.9 Hz), 7.51 (t, 4H, ^3^*J*(^1^H–^1^H) = 7.9 Hz), 7.43–7.34 (m, 12H), 7.07–7.01 (m, 8H), 5.72 (s, 8H); ^13^C{^**1**^H} NMR (100 MHz, (CD_3_)_2_SO, 298 K) δ: 190.7 (d, ^1^*J*(^13^C-^107^Ag) = 176.1 Hz and ^1^*J*(^13^C–^109^Ag) = 213.9 Hz, CAg), 161.8 (d, ^1^*J*(^13^C–^19^F) = 246 Hz, CF), 133.2 (Q), 133.1 (Q), 131.8 (d, ^4^*J*(^13^C–^19^F) = 3.1 Hz, Q), 129.6 (d, ^3^*J*(^13^C–^19^F) = 8.4 Hz, CH), 124.5 (CH), 125.2 (CH), 115.6 (d, ^2^*J*(^13^C–^19^F) = 21.8 Hz, CH), 113.2 (CH), 111.9 (CH), 59.4 (CH_2_), 51.6 (CH_2_); ^19^F{^1^H} NMR (470 MHz, (CD_3_)_2_SO, 298 K) δ: −70.1 (d, ^1^*J*(^19^F–^31^P) = 715.3 Hz, PF_6_), −113.8 (CF); IR (ATR, cm^–1^): υ̃ = 3117 (w, C–H benz), 1605 (m, aromatic C–C), 1510 (s, aromatic C–C), 832 (vs, br, PF_6_); Elemental Analysis Calculated for C_58_H_44_Ag_2_F_16_N_8_P_2_: C, 48.56; H, 3.09; N 7.81%; Analysis Found: C, 48.37; H, 2.93; N 7.49%.

### Single Crystal X-ray Diffraction

A suitable single crystal was selected and immersed in fomblin. The crystal was then mounted to a goniometer head on an XtaLAB Synergy Dualflex, HyPix diffractometer fitted with a Hybrid Pixel Array Detector and a goniometer head using mirror monochromated Mo–Kα radiation (λ = 0.710 73 Å) or Cu–Kα radiation (λ = 1.541 84 Å) sources. The crystal was cooled to 100 K by an Oxford cryostream low temperature device. The full data set was recorded, and the images were processed using CrysAlis Pro.^50^ Structure solution by direct methods was achieved through the use of SHELXT and SHELXL programs,^51,52^ and the structural model was refined by full matrix least-squares on F^2^ using the program Olex2.^53^ Hydrogen atoms were placed using idealized geometric positions (with free rotation for methyl groups), allowed to move in a “riding model” along with the atoms to which they were attached, and refined isotropically. Editing of the CIFs and construction of tables of bond lengths and angles were also achieved using Olex2 1.5.^53^ All molecular images were generated using Mercury 4.0^54^ and the crystal data uploaded to the CSD, with submission numbers 2361333–2361336.

### NMR Stability Studies

**[Ag**_**2**_**(L4)**_**2**_**](PF**_**6**_**)**_**2**_ (5.0 mg) was dissolved in (CD_3_)_2_SO (150 μL) and D_2_O (350 μL), and the sample was mixed well. ^1^H NMR spectra were collected on a 500 MHz NMR spectrometer at 0, 1, and 24 h.

### UV–Vis Stability Studies

Stock solutions of each complex (500 μM) were prepared in DMSO. The stock (100 μL) was added to a quartz cuvette followed by deionized water (1900 μL), giving a final concentration of the complex of 25 μM. Spectra were obtained at 25 °C between 250 and 400 nm at 0, 10, 20, and 30 min and 1, 2, 4, 6, and 24 h after addition.

### Cell Viability Assay

All cytotoxicity assays were conducted using human cell lines: breast adenocarcinomas (MDA-MB-231 and MCF-7) and non-cancerous epithelial retinal (ARPE-19) cell lines. All cell lines were routinely maintained as monolayer cultures in an appropriate complete medium: MCF-7 and ARPE-19 in high glucose DMEM complete medium (including 10% FBS, 1 mM sodium pyruvate, 2 mM L-glutamine and 1% pen-strep) and MDA-MB-231 in RPMI-1640 complete medium (including 10% FBS, 1 mM sodium pyruvate, 2 mM L-glutamine and 1% pen-strep) and grown in either T-25 or T-75 flasks at 37 °C and 5% CO_2_. Prior to chemosensitivity studies, cell monolayers were passaged using Trypsin-EDTA (0.05%) and diluted to a concentration of 4 × 10^4^ cells/mL. All assays were conducted using 96-well plates, in which 100 μL of the cell suspension was added for 24 h at 37 °C and 5% CO_2_ and then 100 μL of compound/media dilutions for a further 24 h (compound stocks were made using sterile DMSO at 100 mM prior to dilution and a maximum of 0.1% DMSO used in each assay). After 24 h, MTT (3-(4,5-dimethylthiazol-2-yl)-2,5-diphenyltetrazolium bromide (20 μL, 5 mg/mL) was added to each well and incubated for 3 h at 37 °C and 5% CO_2_. All solutions were then aspirated and DMSO (150 μL) added to each well, and the absorbance measured at 540 nm using a ClarioStar spectrophotometer microplate reader. Results were plotted on a logarithmic scale, and the half maximal inhibitory concentration (IC_50_) was determined from triplicate of triplicate repeats (*n* = 9) and reported as an IC_50_ ± Standard Deviation (SD).

### Cell Uptake Studies

MDA-MB-231 and MCF-7 cells were maintained as described in the cell culture protocol above. All assays were conducted in 6-well plates, and cells were diluted to a concentration of 1 × 10^6^ cells/well in 1 mL of complete media. After 24 h, complex **[Ag(L2)**_**2**_**](PF**_**6**_**)** or **[Ag**_**2**_**(L4)**_**2**_**](PF**_**6**_**)**_**2**_ (1 μM) was added to the plates for 1 h. The media was removed, and the cells were washed with PBS (2 × 1 mL) and trypsin-EDTA (0.05%) added for 2–3 min. Complete medium was added to dilute the trypsin, and the cell suspensions collected in Falcon tubes. The samples were centrifuged at 1500 rpm for 5 min and then resuspended and washed with PBS and gently vortexed in between. This wash step was repeated three times to remove the residual silver complex. Cell pellets were digested by addition of ultrapure grade 67% nitric acid (100 μL, Romil) with heating at 70 °C for 1 h, followed by addition of ultratrace grade 30% hydrogen peroxide (100 μL, Merck) and heating at 70 °C for a further 4 h. The digested samples were diluted to 2% nitric acid prior to analysis. A range of calibration standards were prepared by dilution of multielemental standard solution IV (Merck). Analysis of the ^107^Ag isotope was performed on a sector-field ICP-MS (Element 2XR, Thermo Scientific). The instrument was fitted with a cyclonic spray chamber (Glass Expansion), conical glass concentric nebulizer (Glass Expansion), and 0.25 I.D. probe (Elemental Scientific). All results are presented as Ag atoms/cell from triplicate repeats.

### Cell Images

MDA-MB-231 cells (maintained as described above) were seeded at a concentration of 4 × 10^5^ cells/well in 1 mL of complete media in 6-well plates, and then incubated at 37 °C and 5% CO_2_ for 24 h. The medium was removed, and the cells were washed with PBS (1 mL). Complete media (1 mL) spiked with the drug concentrations of 10 μM, 50 μM or DMSO (control at a maximum of 0.05%) were added. The images were taken on a GXCAM digital camera mounted on a ZEISS Primo Vert microscope at a magnification of 10× after 0, 1, and 4 h.

### Reactive Oxygen Species (ROS)

MDA-MB-231 cells (maintained as described above) were seeded at a concentration of 8 × 10^3^ cells/well in indicator-free complete RPMI (100 μL) and 96-well optical bottom plates, and incubated at 37 °C and 5% CO_2_ for 2 days, after which complete RPMI (100 μL) spiked with Ag(I)-NHC complex (concentration of 1, 2, and 5 × the IC_50_ or DMSO 0.1% for the control) was added. The cells were incubated for 3.5 h. To the wells, H_2_DCFDA (20 μL) in PBS:DMSO (91:3) was added to give a final dye concentration of 20 μM. The cells were incubated with the dye for 30 min, after which the media was removed, and the wells were washed with PBS (3 × 200 μL) and complete RMPI (100 μL) was added. The cells were imaged on an Observer-7 microscope, ex: 494 nm and em: 512 nm.

### DNA Interactions

In a Tris·HCl (10 mM, pH 7.4) buffer:DMSO (95:5) solution (2000 μL) at 22 °C, ct-DNA (27.7. μM) and EtBr (11.3 μM) were combined and mixed well and left to equilibrate in darkness for 30 min after which a fluorescence spectrum was taken on a FS5 Spectrofluorometer (ex: 545 nm) between 575 and 700 nm. **[Ag(L4)**_**2**_**](PF**_**6**_**)**_**2**_ (2 μL at 1 mM in DMSO) was titrated into the sample, and the sample was mixed well and left to equilibrate for 5 min after which a further spectrum was taken; this step was repeated with 2 μL additions, up to a total of 26 μL of complex.

### Molecular Docking and DFT Calculations

The crystal structure of a DNA dodecamer was retrieved from the Protein Data Bank (http://www.rcsb.org PDB ID: 1LU5). The molecular structure of the most selective complex **[Ag**_**2**_**(L4)**_**2**_**](PF**_**6**_**)**_**2**_ was obtained using DFT calculations, performed in ORCA 5.0.4.^55^ The compounds were optimized without any constraint using the PBE0 functional^56^ and the def2-TZVP basis set combined with a 28-electron effective core potential (def2-ECP)^57^ for Ag and the def2-SVP basis set for all other atoms.^58^ The Chain of Spheres Exchange variant of the Resolution of Identity approximation (RIJCOSX) was employed to speed up the calculations using the def2/J auxiliary basis set.^59^ The optimized structures are identified as true ground states by the absence of negative Eigenvalues in the frequency calculation. The CIF (scXRD) and XYZ (DFT) files were converted to the PDB format using UCSF Chimera,^60^ where counterions were removed for simplicity (http://www.cgl.ucsf.edu/chimera/). The molecular docking study was performed using AutoDock 4.2.6 software^61^ using the Lamarckian Genetic Algorithm. The DNA structure was kept rigid, while the metal complex was allowed to have rotatable bonds. The grid map was set to 76 × 62 × 104 Å^3^ along the *x*, *y*, and *z* axes with 0.375 Å grid spacing. The analysis was performed at least 3 times to confirm reproducibility with the lowest free binding energy conformation reported here. PyMOL (The PyMOL Molecular Graphics System, Version 2.5.2 Schrödinger, LLC.)^62^ was used to produce images.

## Supporting Information Available

Modified protocols for the synthesis of known compounds, NMR and FTIR spectra, crystallographic tables, decomposition studies by ^1^H NMR and UV/vis, detailed cytotoxicity, DNA interaction titration, ROS results, and docking results (PDF)
*xyz*-Coordinates derived from DFT (XYZ)

## Supplementary Material

om4c00292_si_001.pdf

om4c00292_si_002.xyz
