# Supplementary material for: Fluorinated N-Heterocyclic Carbene Silver(I) Complexes with High Cancer Cell Selectivity
Source: Organometallics. 2024 Sep 19;43(20):2662–73. doi: 10.1021/acs.organomet.4c00292 (PMC11523213; doi:10.1021/acs.organomet.4c00292)
Supplement: Supplementary file 1 — om4c00292_si_001.pdf [file om4c00292_si_001.pdf]

Supporting Information

Fluorinated *N*-heterocyclic carbene silver(I) complexes with high cancer cell selectivity

Oliver S. King,[a] Benjamin J. Hofmann,[a] Aran E. Boakye-Smith,[a] Amy J. Managh,[b] Tameryn Stringer[a] and Rianne M. Lord[a*]

[a] School of Chemistry, Pharmacy and Pharmacology, University of East Anglia, Norwich, Norfolk, NR1 1GE, United Kingdom

[b] Department of Chemistry, School of Science, Loughborough University, Loughborough, Leicestershire, LE11 3TU, United Kingdom.

Email: r.lord@uea.ac.uk

Table of Contents S

Synthetic Procedures for known Compounds *S3*

Ligand 1, HL1(Br) S3

Ligand 2, HL2(Br) S3

Ligand 3, $H_2L3(Br)_2$ S3

Ligand 5, $H_2L5(Br)_2$ and $H_2L5(PF_6)_2$ S3

Complex 5, $[Ag_2(L5)_2](PF_6)_2$ S4

NMR Spectroscopy *S5*

1,3-Bis-(4-fluorobenzyl)imidazolium bromide - HL1(Br) S5

1,3-Bis-(4-fluorobenzyl)imidazolium hexafluorophosphate - $HL1(PF_6)$ S5

$[Ag(L1)_2](PF_6)$ S7

1,3-Bis-(4-fluorobenzyl)benzimidazolium bromide - HL2(Br) S8

1,3-Bis-(4-fluorobenzyl)benzimidazolium hexafluorophosphate - $HL2(PF_6)$ S9

$[Ag(L2)_2](PF_6)$ S10

1,1′-Bis-(4-fluorobenzyl)-3,3′-methylenediimidazolium dibromide – $H_2L3(Br)_2$ S12

1,1′-Bis-(4-fluorobenzyl)-3,3′-methylenediimidazolium dihexafluorophosphate – $H_2L3(PF_6)_2$ ... S12

$[Ag_2(L3)_2](PF_6)_2$ S14

1,1′-Bis-(4-fluorobenzyl)-3,3′-methylenedibenzimidazolium dibromide – $H_2L4(Br)_2$ S15

1,1′-Bis-(4-fluorobenzyl)-3,3′-methylenedibenzimidazolium dihexafluorophosphate – $H_2L4(PF_6)_2$ S17

$[Ag_2(L4)_2](PF_6)_2$ S18

1,1′-Bis-(benzyl)-3,3′-methylenediimidazolium dibromide – $H_2L5(Br)_2$ S20

1,1′-Bis-(benzyl)-3,3′-methylenediimidazolium dihexafluorophosphate – $H_2L5(PF_6)_2$ S20

$[Ag_2(L5)_2](PF_6)_2$ S21

ATR-FTIR Spectroscopy *S21*

Single Crystal X-ray Diffraction *S24*

Cytotoxicity Results *S26*

Optical Microscope Images *S28*

NMR Spectroscopy – Stability Studies *S31*

UV/Vis Spectroscopy – Stability Studies *S32*

Reactive Oxygen Species *S35*

EtBr titrations *S36*

Docking Studies *S37*

Synthetic Procedures for known Compounds

Compounds **HL1(Br)**,[1] **HL2(Br)**,[2] **H_2L3(Br)$_2$**,[3] **H_2L5(Br)$_2$, H_2L5(PF_6)$_2$**,[4] and **[Ag_2(L5)$_2$](PF_6)$_2$**,[5] have been synthesized via literature methods and successful synthesis was confirmed by ^{1}H NMR spectroscopy and elemental analysis.

Ligand 1, HL1(Br)

1-[(4-Fluorophenyl)methyl]-1H-imidazole (1.01 g, 5.73 mmol) was dissolved in toluene (10 mL) and added to the stirring solution 4-fluorobenzyl bromide (714 µL, 5.73 mmol). The mixture was heated to reflux for 23 h. The reaction mixture was allowed to cool down to room temperature, the supernatant was discarded, and the resulting brown oil was washed with acetone (20·2 mL) discarding supernatant both times. The brown oil was dried *in vacuo* yielding a waxy brownish-yellow solid. **Yield:** 0.85 g, 2.31 mmol, 40%; **^{1}H NMR (500 MHz, $(CD_3)_2SO$, 298 K) δ:** 9.45 (t, 1H, ^4J(^{1}H-^{1}H) = 1.5 Hz), 7.85 (d, 2H, ^4J(^{1}H-^{1}H) = 1.5 Hz), 7.56 – 7.51 (m, 4H), 7.24 – 7.30 (m, 4H), 5.44 (s, 4H).

Ligand 2, HL2(Br)

1-[(4-Fluorophenyl)methyl]-1*H*-benzimidazole (2.03 g, 8.98 mmol) and 4-fluorobenzyl bromide (1.68 g, 8.91 mmol) were dissolved in acetonitrile (10 mL) and heated to reflux for 24 h. The mixture was allowed to cool down to room temperature and the colorless precipitate was collected by vacuum filtration and washed with diethyl ether (3·20 mL) and dried *in vacuo* yielding a colorless crystalline solid. **Yield:** 2.62 g, 6.31 mmol, 71%; **^{1}H NMR (400 MHz, $(CD_3)_2SO$, 298 K) δ:** 10.13 (s, 1H), 8.00 (dd, 2H, ^3J(^{1}H-^{1}H) = 6.0 Hz, ^4J(^{1}H-^{1}H) = 3.0 Hz), 7.68 – 7.61 (m, 6H), 7.31 – 7.24 (m, 4H), 5.80 (s, 4H).

Ligand 3, H_2L3(Br)$_2$

1-[(4-Fluorophenyl)methyl]-1*H*-imidazole (0.60 g, 3.39 mmol) was dissolved in dibromomethane (10 mL) and heated to reflux for 18 h. The light brown suspension was allowed to cool to room temperature and collected by vacuum filtration. The filter cake is washed with acetone (3·40 mL) and diethyl ether (3·40 mL) and dried *in vacuo* yielding a colorless powder. **Yield:** 0.64 g, 1.21 mmol, 71%; **^{1}H NMR (400 MHz, $(CD_3)_2SO$, 298 K) δ:** 9.64 (t, 2H, ^4J(^{1}H-^{1}H) = 1.6 Hz), 8.09 (dd, 2H, ^3J(^{1}H-^{1}H) = 2.1 Hz, ^4J(^{1}H-^{1}H) = 1.6 Hz), 7.91 (dd, 2H, ^3J(^{1}H-^{1}H) = 2.1 Hz, ^4J(^{1}H-^{1}H) = 1.6 Hz), 7.60 – 7.54 (m, 4H), 7.29 (m, 4H), 6.69 (s, 2H), 5.50 (s, 4H).

Ligand 5, H_2L5(Br)$_2$ and H_2L5(PF_6)$_2$

H_2L5(Br)$_2$: 1-[(Phenyl)methyl]-1H-benzimidazole (0.533 g, 3.37 mmol) was heated in dibromomethane (10 mL) to reflux for 24 h. After cooling down, the formed yellowish precipitate is filtered and washed with acetone (3·40 mL) and diethyl ether (3·40 mL) to yield a white powder. **Yield:** 0.575 g, 1.17 mmol, 70%; **^{1}H NMR (400 MHz, $(CD_3)_2SO$, 298 K) δ:** 9.61 (app. t, 2H, ^4J(^{1}H-^{1}H) = 1.7 Hz), 8.07 (app. t, 2H, ^3J(^{1}H-^{1}H) = 1.9 Hz), 7.91 (dd, 2H, ^3J(^{1}H-^{1}H) = 2.1 Hz, ^4J(^{1}H-^{1}H) = 1.6 Hz), 7.48 – 7.40 (m, 10H), 6.67 (s, 2H), 5.50 (s, 4H). **H_2L5(PF_6)$_2$:** 1,1′-Dibenzyl-3,3′-methylenediimidazolium dibromide (0.575 g, 1.17 mmol) and ammonium hexafluorophosphate (689 mg, 4.22 mmol) were stirred in acetone (50 mL) for 1 h. After filtration, the solvent was removed to yield a white powder. The powder was stirred in water (50 mL) for one hour and collected by filtration. After washing with water (2·40 mL) and diethyl ether (3·40 mL) and drying, a white solid was obtained. **Yield:** 0.443 g, 0.71 mmol, 61%; **^{1}H NMR (400 MHz, $(CD_3)_2SO$, 298 K) δ:** 9.45 (app. t, 2H, ^4J(^{1}H-^{1}H) = 1.7 Hz), 7.98 (dd, 2H, ^3J(^{1}H-

^{1}H) = 2.1 Hz, $^4J(^1H-^1H)$ = 1.6 Hz), 7.88 (dd, 2H, $^3J(^1H-^1H)$ = 2.1 Hz, $^4J(^1H-^1H)$ = 1.6 Hz), 7.48 – 7.40 (m, 10H), 6.58 (s, 2H), 5.48 (s, 4H); **Elemental Analysis: Calculated for $C_{21}H_{22}F_{12}N_4P_2$:** C, 40.66; H, 3.57; N 9.03%; **Analysis Found:** C, 40.70; H, 3.21; N 8.78%.

Complex 5, $[Ag_2(L5)_2](PF_6)_2$

1,1′-Dibenzyl-3,3′-methylenediimidazolium dibromide (0.600 g, 1.19 mmol) and silver(I) oxide (0.709 g, 3.06 mmol) were stirred in methanol (10 mL) under the exclusion of light for 22 h. The resulting brown suspension was passed through Celite® twice, giving a pale-yellow solution. Methanol was added to an overall volume of 15 mL and an aqueous solution of ammonium hexafluorophosphate (0.417 g, 2.56 mmol, 25 mL) was added upon stirring to form a colourless precipitate. After 2 h the precipitate was collected by vacuum filtration, washed with deionized water (3·40 mL) and diethyl ether (3·40 mL) and dried *in vacuo* giving a colorless powder. **Yield:** 0.330 g, 0.28 mmol, 46%. **^{1}H NMR (400 MHz, $(CD_3)_2SO$, 298 K) δ:** 7.88 (app. t, 4H, $^3J(^1H-^1H)$ = 1.6 Hz), 7.61 (app. t, 4H, $^3J(^1H-^1H)$ = 1.6 Hz), 7.28 – 7.18 (m, 12H), 7.09 – 7.06 (m, 8H), 6.88 (br. s, 2H), 6.49 (br. s, 2H), 5.21 (s, 8H); **Elemental Analysis: Calculated for $C_{42}H_{40}Ag_2F_{12}N_8P_2$**: C, 43.39; H, 3.47; N 9.64%; **Analysis Found:** C, 43.68; H, 2.93; N 9.50%.

(1) Vlahakis, J. Z.; Lazar, C.; Crandall, I. E.; Szarek, W. A. Anti-Plasmodium activity of imidazolium and triazolium salts. *Bioorg. Med. Chem.* **2010**, *18* (16), 6184-6196. DOI: 10.1016/j.bmc.2010.05.020.
(2) Guo, F.; Yang, Y. Method for synthesizing N,N'-disubstituted benzimidazolium derivative and metal organic salt thereof by mechanochemical method. China CN111635363A, 2020.
(3) Lee, H. M.; Lu, C. Y.; Chen, C. Y.; Chen, W. L.; Lin, H. C.; Chiu, P. L.; Cheng, P. Y. Palladium complexes with ethylene-bridged bis(N-heterocyclic carbene) for C–C coupling reactions. *Tetrahedron* **2004**, *60* (27), 5807-5825. DOI: 10.1016/j.jorganchem.2020.121643.
(4) Noujeim, N.; Leclercq, L. c.; Schmitzer, A. R. N,N'-Disubstituted Methylenediimidazolium Salts: A Versatile Guest for Various Macrocycles. *J. Org. Chem.* **2008**, *73* (10), 3784-3790. DOI: 10.1021/jo702683c.
(5) Lum, R.; Zhang, H.; Zhang, W.; Bai, S.-Q.; Zhao, J.; Hor, T. S. A. Trans [O✓ReV-OH] core stabilised by chelating N-heterocyclic dicarbene ligands. *Dalton Trans.* **2013**, *42* (4), 871-873, 10.1039/C2DT31681A. DOI: 10.1039/C2DT31681A.

NMR Spectroscopy

1,3-Bis-(4-fluorobenzyl)imidazolium bromide - HL1(Br)

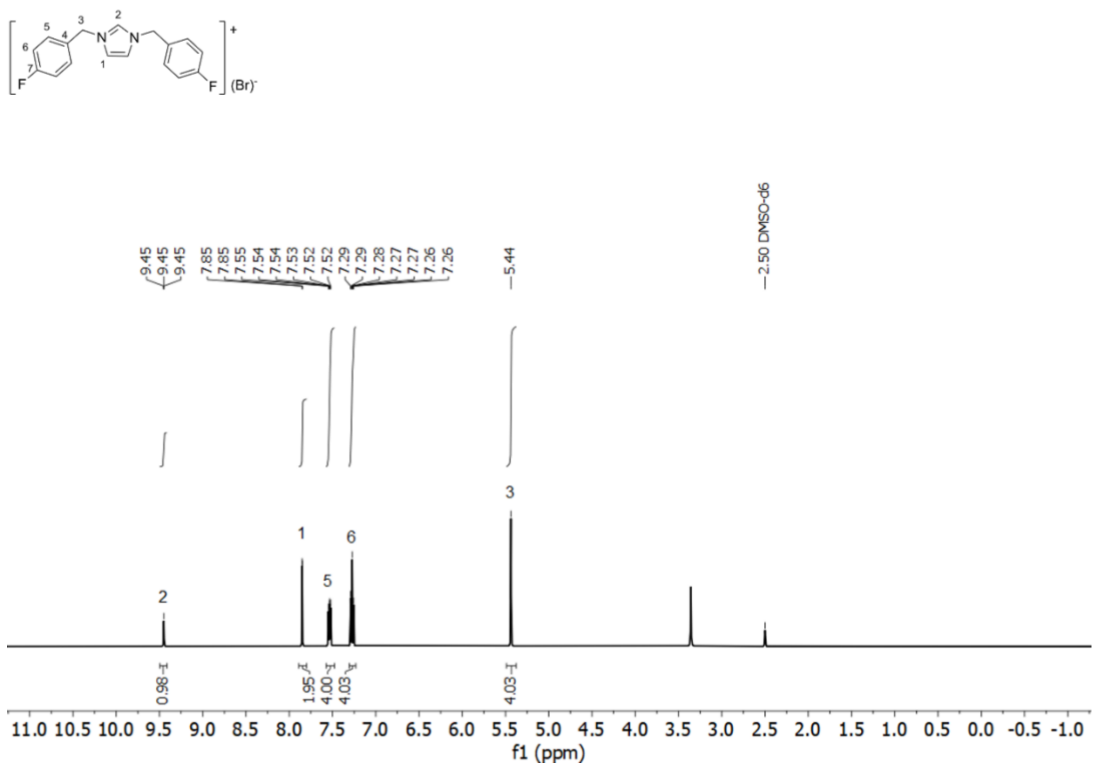

Figure S 1: ^{1}H NMR spectrum of **HL1(Br)** (500 MHz, $(CD_3)_2SO$, 298 K)

1,3-Bis-(4-fluorobenzyl)imidazolium hexafluorophosphate - HL1(PF_6)

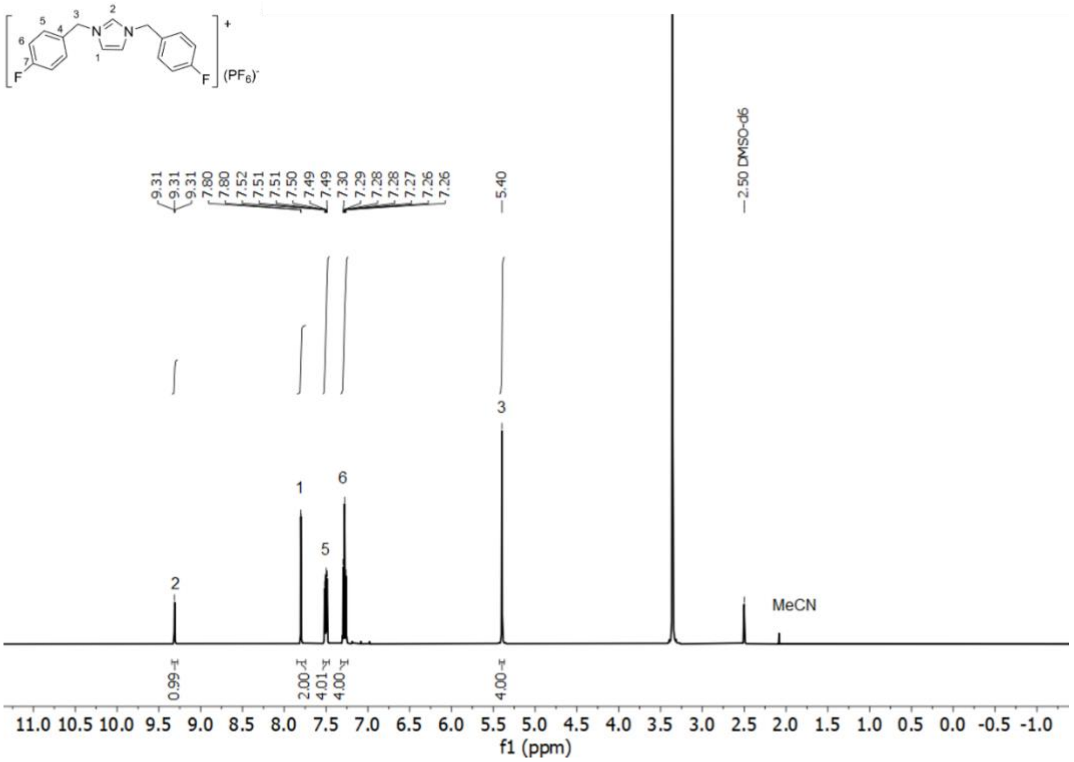

Figure S 2: ^{1}H NMR spectrum of **HL1(PF_6)** (500 MHz, $(CD_3)_2SO$, 298 K)

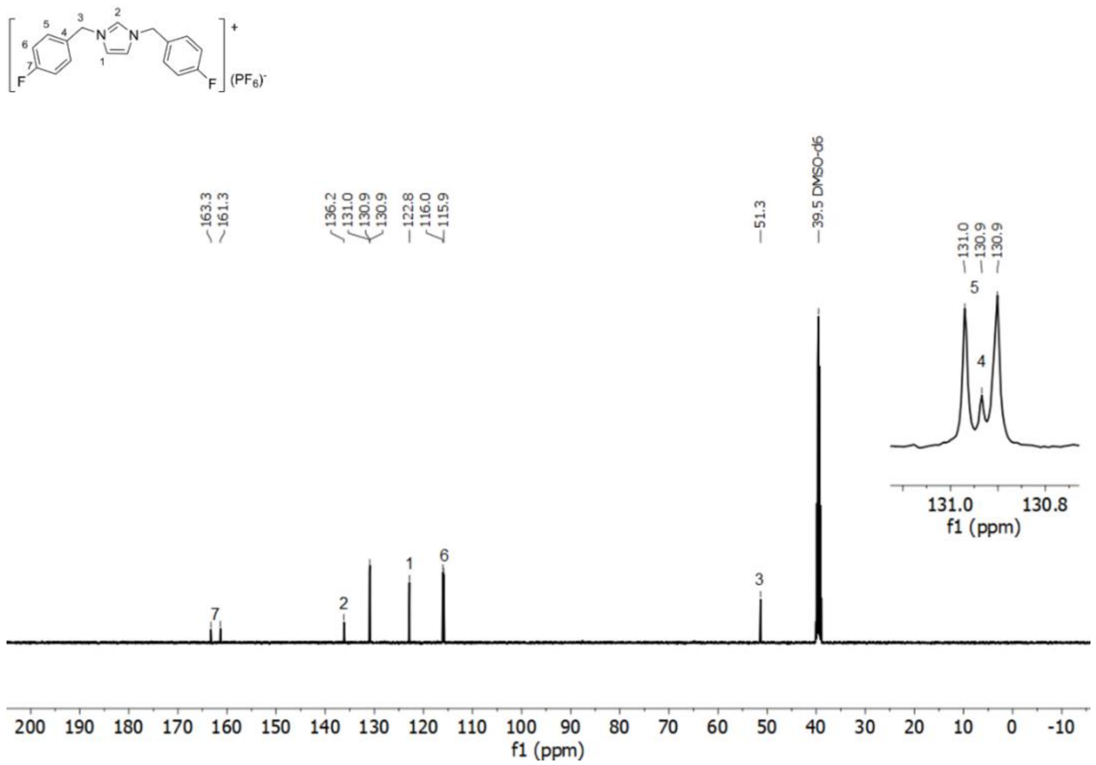

Figure S 3: $^{13}C\{^{1}H\}$ NMR spectrum of **HL1(PF$_6$)** (126 MHz, $(CD_3)_2SO$, 298 K)

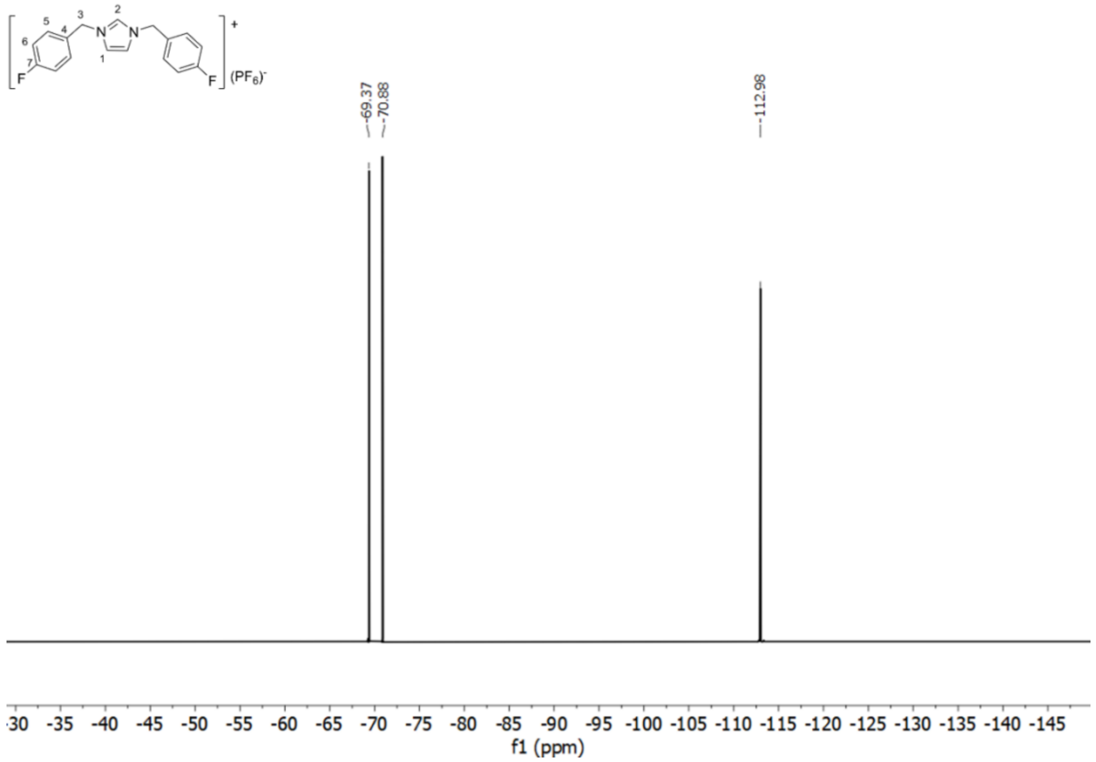

Figure S 4: $^{19}F\{^{1}H\}$ NMR spectrum of **HL1(PF$_6$)** (471 MHz, $(CD_3)_2SO$, 298 K)

[Ag(L1)$_2$](PF$_6$)

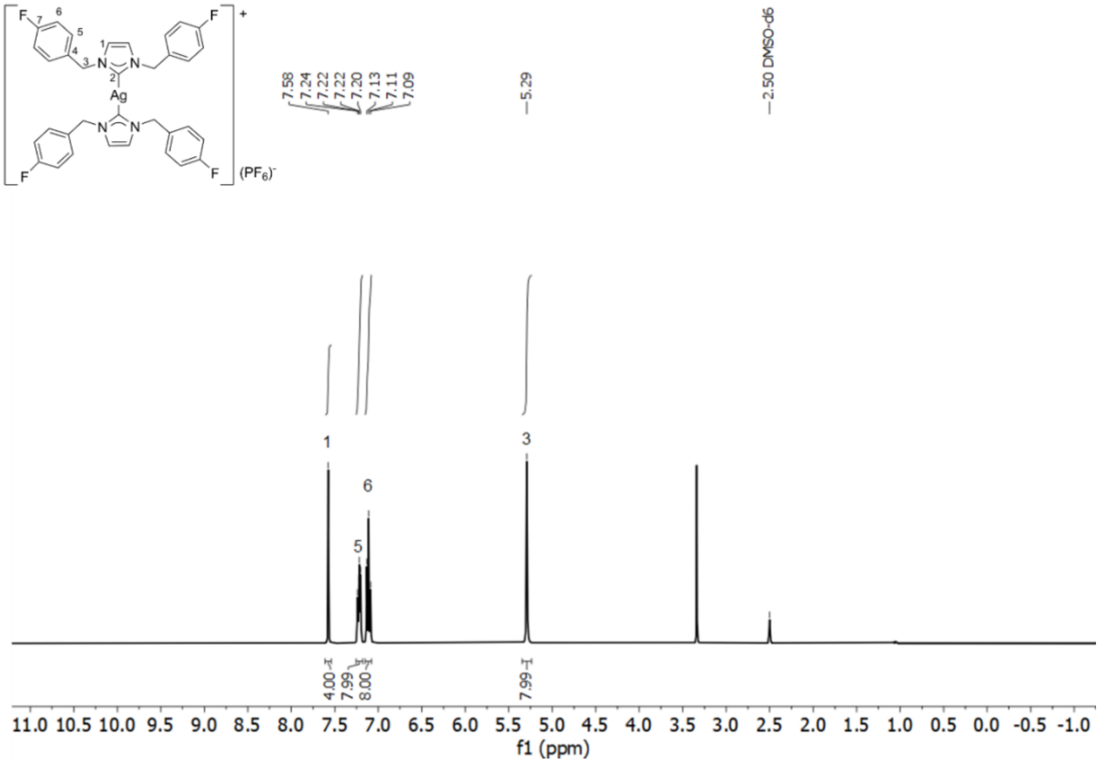

Figure S 5: ^{1}H NMR spectrum of **[Ag(L1)$_2$](PF$_6$)** (400 MHz, $(CD_3)_2SO$, 298 K)

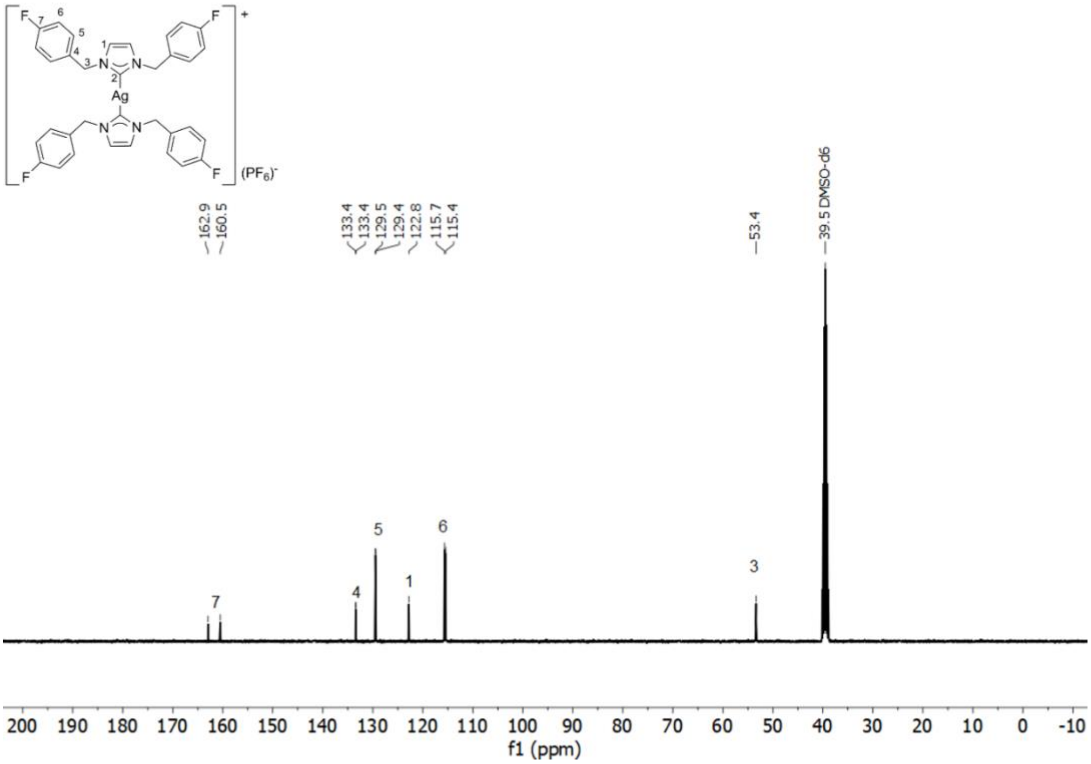

Figure S 6: ^{13}C{^{1}H} NMR spectrum of **[Ag(L1)$_2$](PF$_6$)$_2$** (101 MHz, $(CD_3)_2SO$, 298 K)

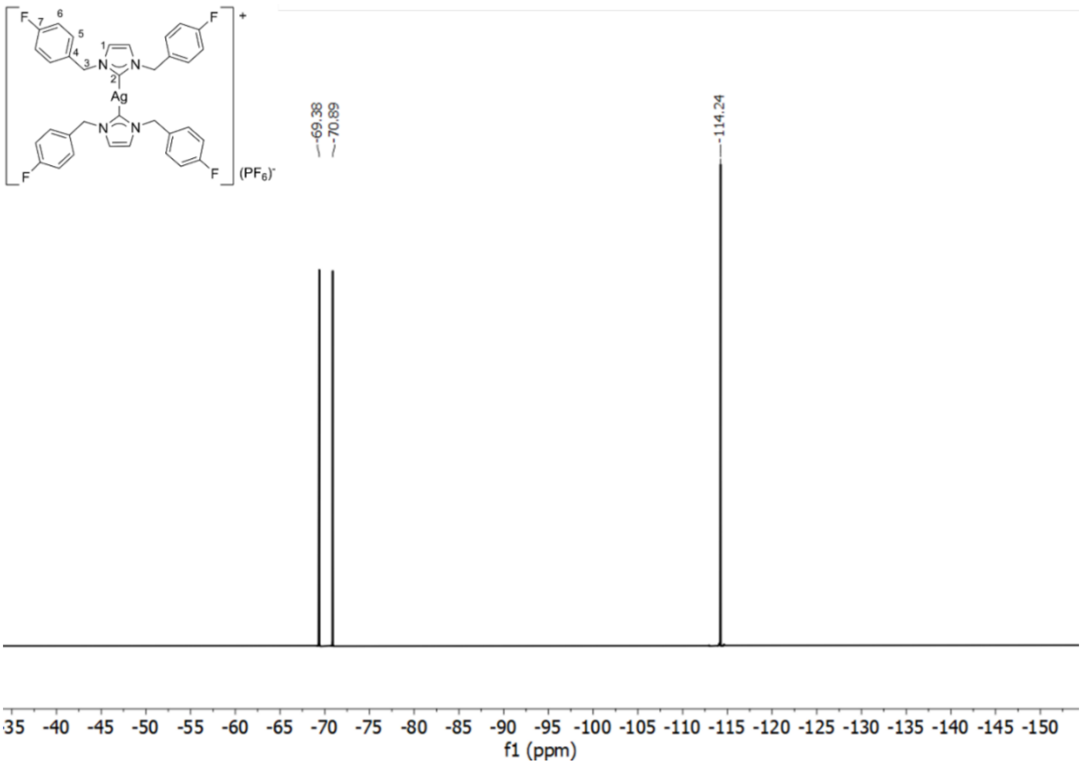

Figure S 7: $^{19}F\{^{1}H\}$ NMR spectrum of **[Ag(L1)$_2$](PF$_6$)** (471 MHz, $(CD_3)_2SO$, 298 K)

1,3-Bis-(4-fluorobenzyl)benzimidazolium bromide - HL2(Br)

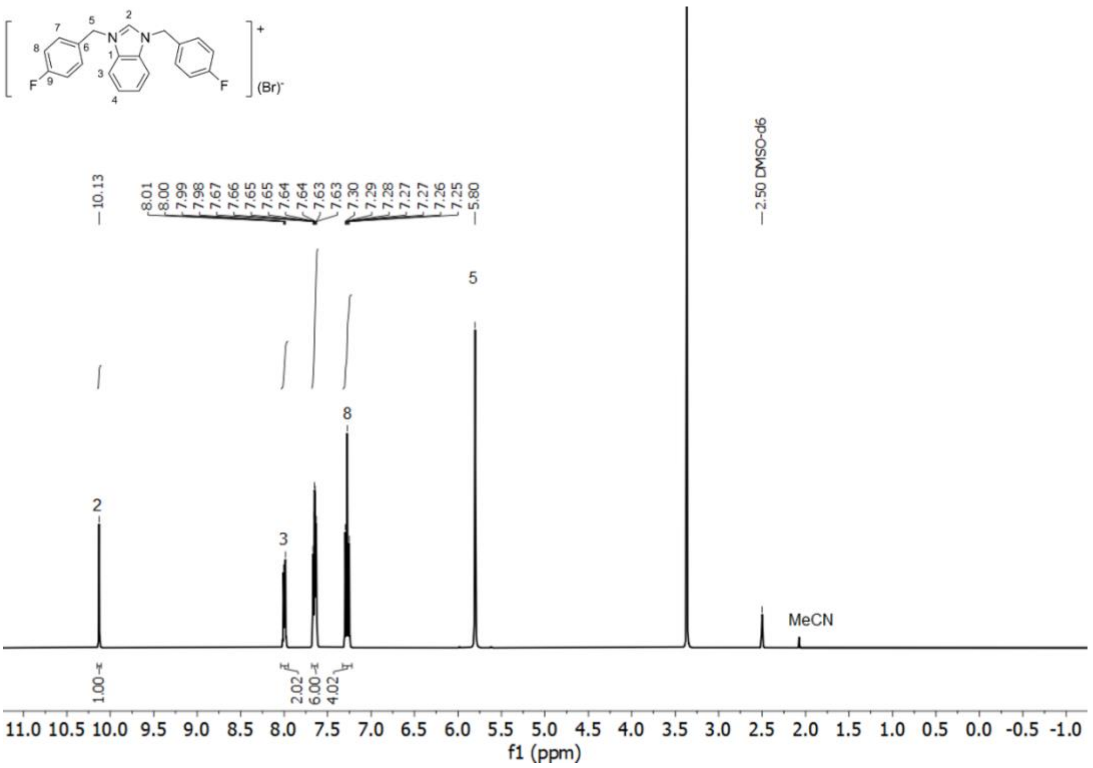

Figure S 8: ^{1}H NMR spectrum of **HL2(Br)** (500 MHz, $(CD_3)_2SO$, 298 K)

1,3-Bis-(4-fluorobenzyl)benzimidazolium hexafluorophosphate - HL2(PF_6)

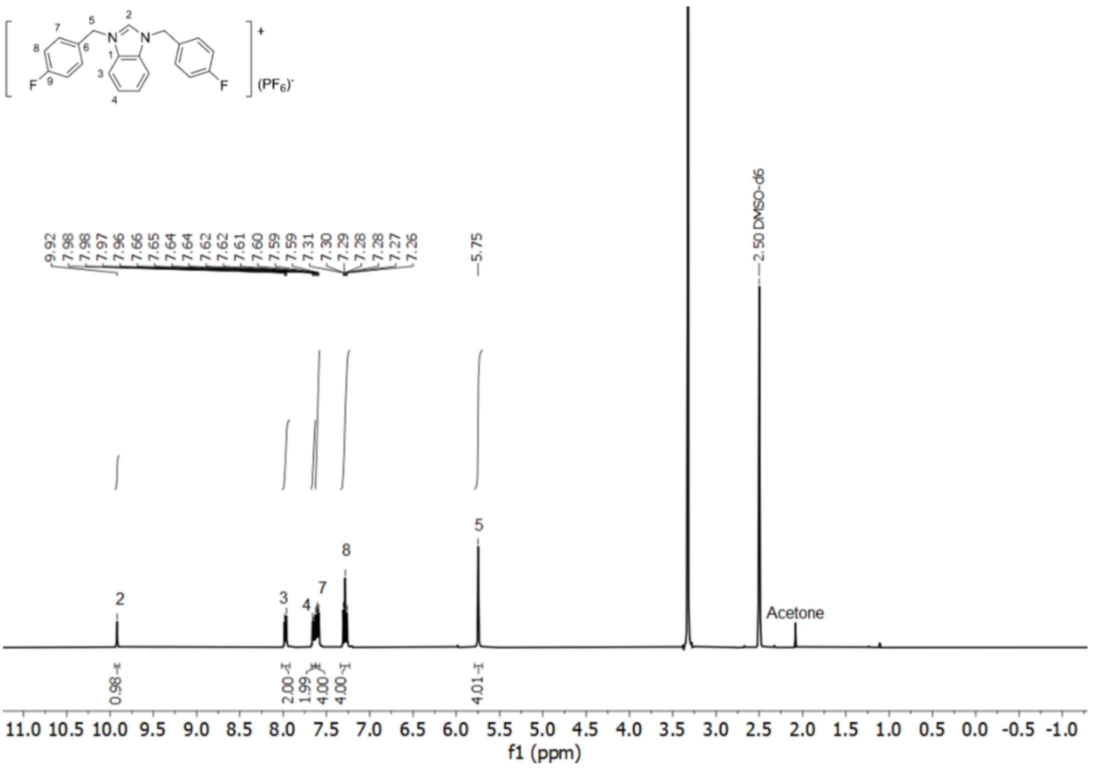

Figure S 9: ^{1}H NMR spectrum of **HL2(PF_6)** (500 MHz, $(CD_3)_2SO$, 298 K)

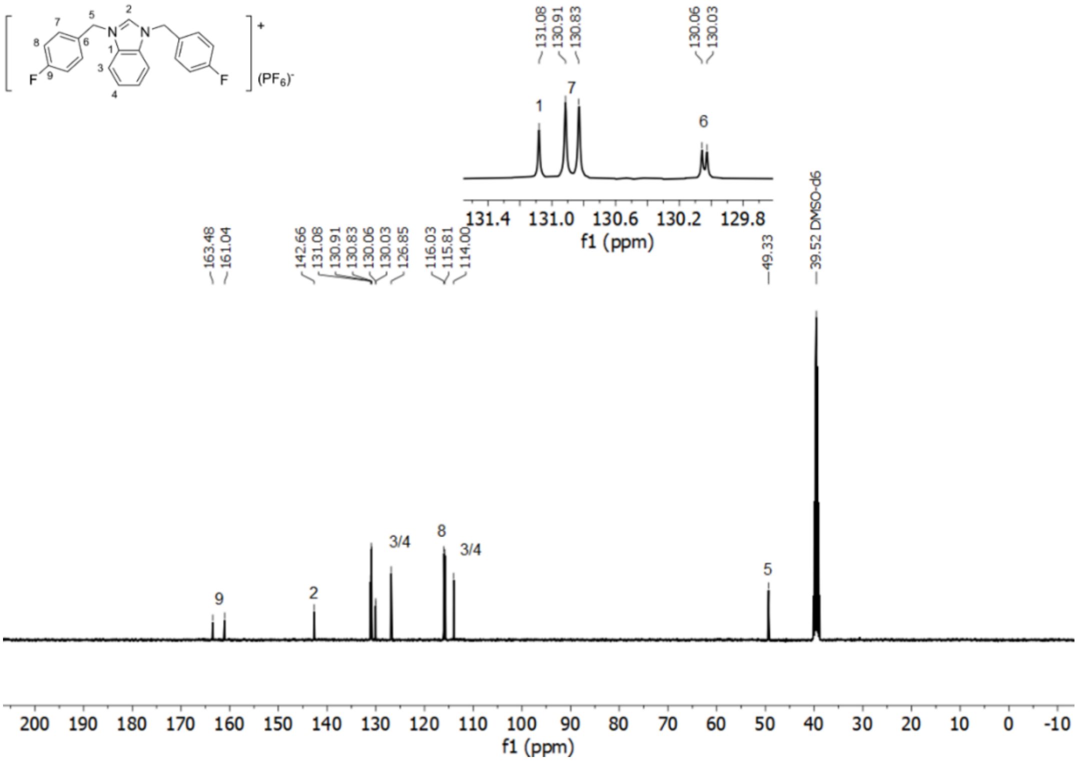

Figure S 10: $^{13}C\{^{1}H\}$ NMR spectrum of **HL2(PF_6)** (126 MHz, $(CD_3)_2SO$, 298 K)

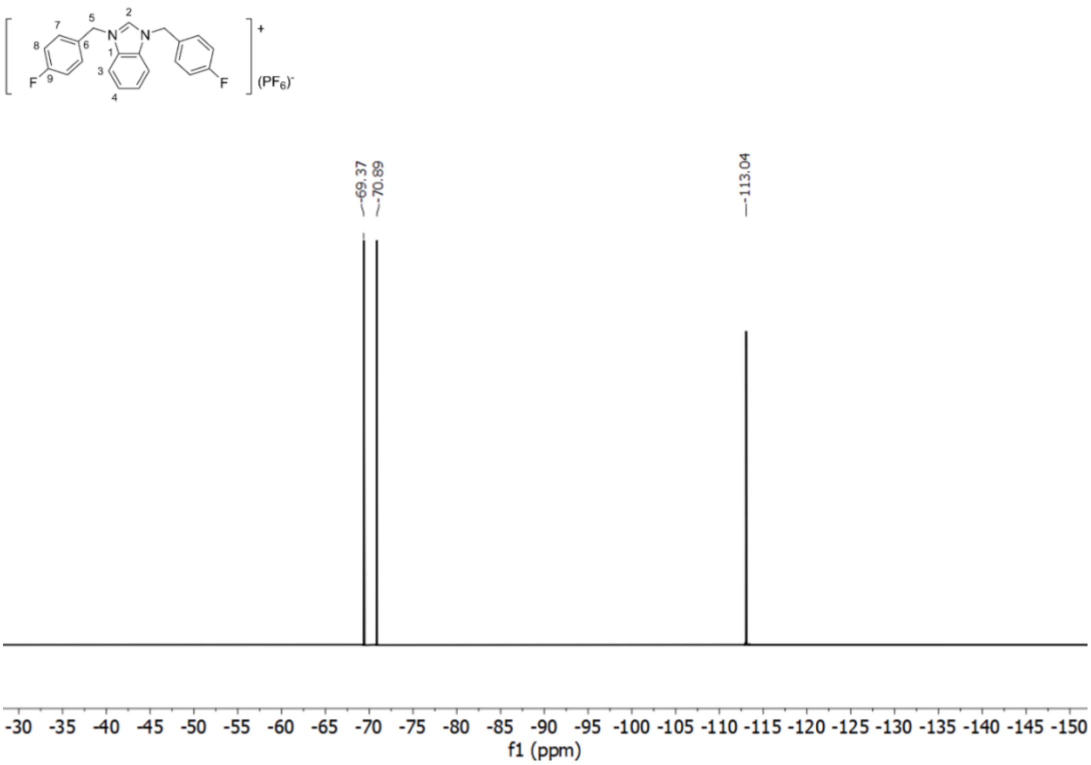

Figure S 11: $^{19}F\{^{1}H\}$ NMR spectrum of **HL2(PF$_6$)** (471 MHz, $(CD_3)_2SO$, 298 K)

[Ag(L2)$_2$](PF$_6$)

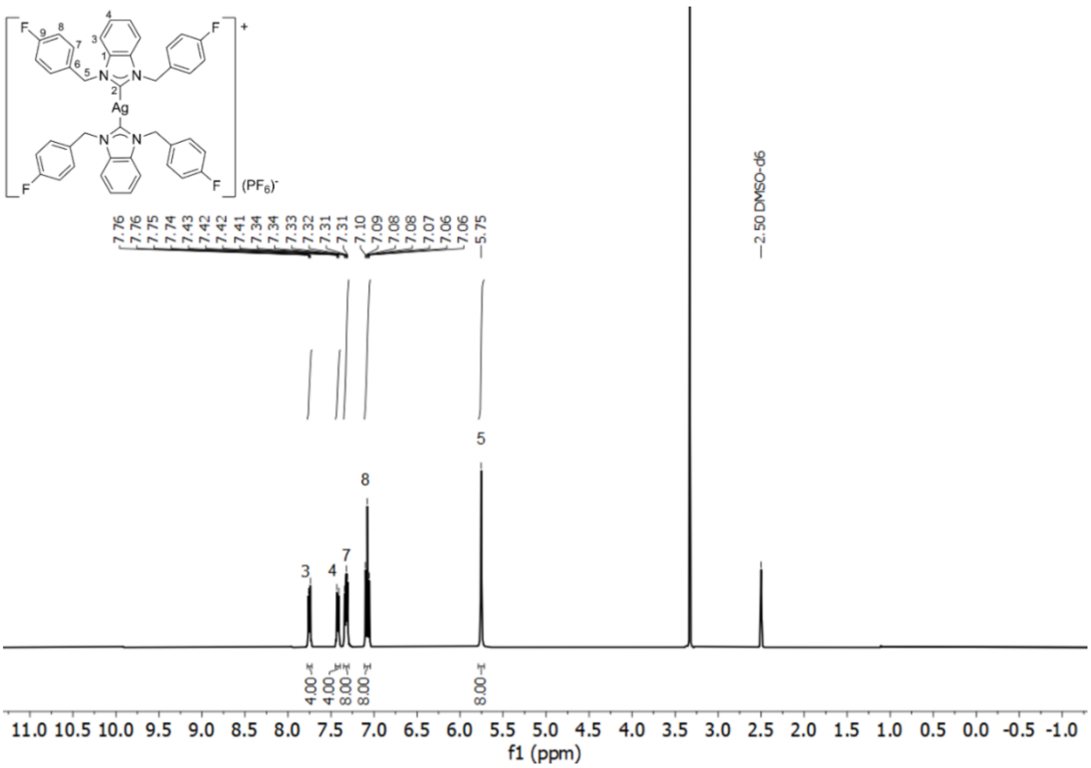

Figure S 12: ^{1}H NMR spectrum of **[Ag(L2)$_2$](PF$_6$)** (400 MHz, $(CD_3)_2SO$, 298 K)

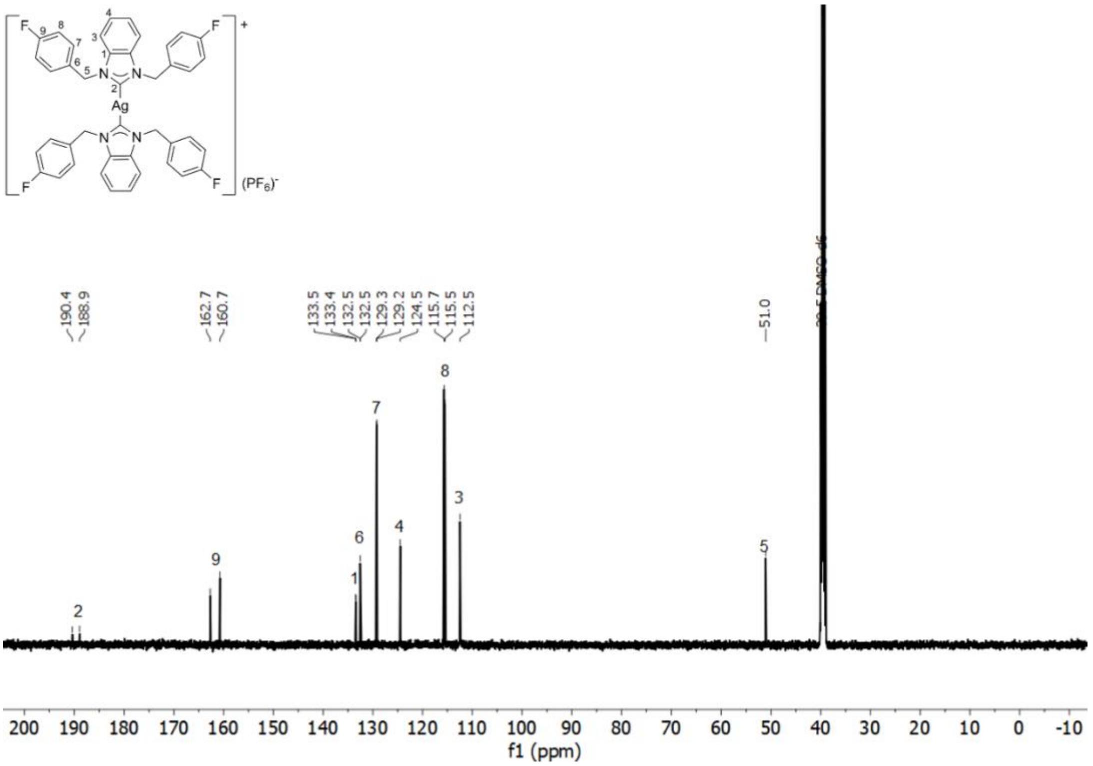

Figure S 13: $^{13}C\{^{1}H\}$ NMR spectrum of **[Ag(L2)$_2$](PF$_6$)** (126 MHz, $(CD_3)_2SO$, 298 K)

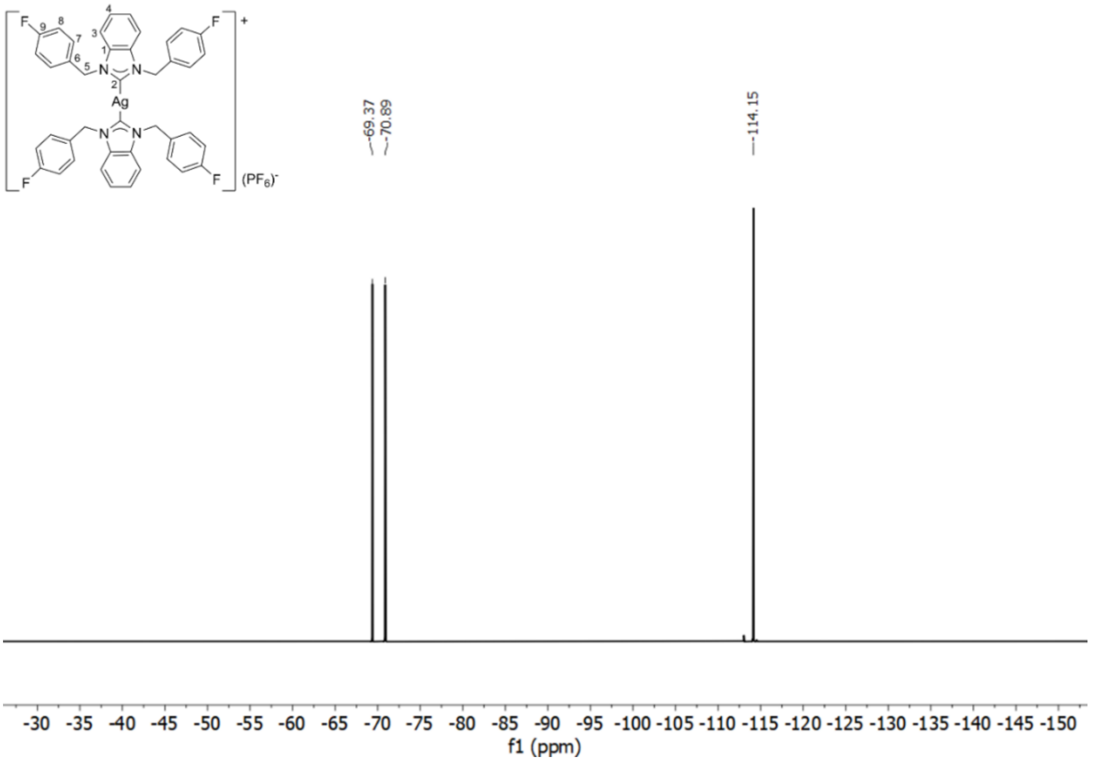

Figure S 14: $^{19}F\{^{1}H\}$ NMR spectrum of **[Ag(L2)$_2$](PF$_6$)** (471 MHz, $(CD_3)_2SO$, 298 K)

1,1′-Bis-(4-fluorobenzyl)-3,3′-methylenediimidazolium dibromide – $H_2L3(Br)_2$

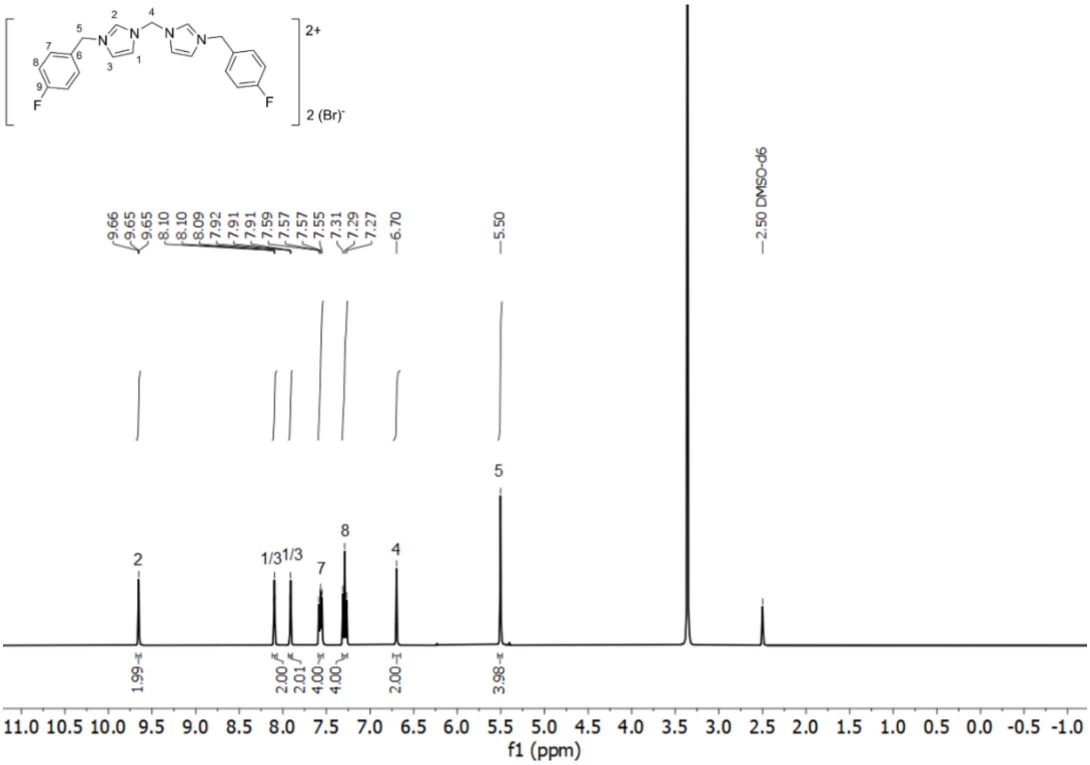

Figure S 15: ^1H NMR spectrum of **$H_2L3(Br)_2$** (400 MHz, $(CD_3)_2SO$, 298 K)

1,1′-Bis-(4-fluorobenzyl)-3,3′-methylenediimidazolium dihexafluorophosphate – $H_2L3(PF_6)_2$

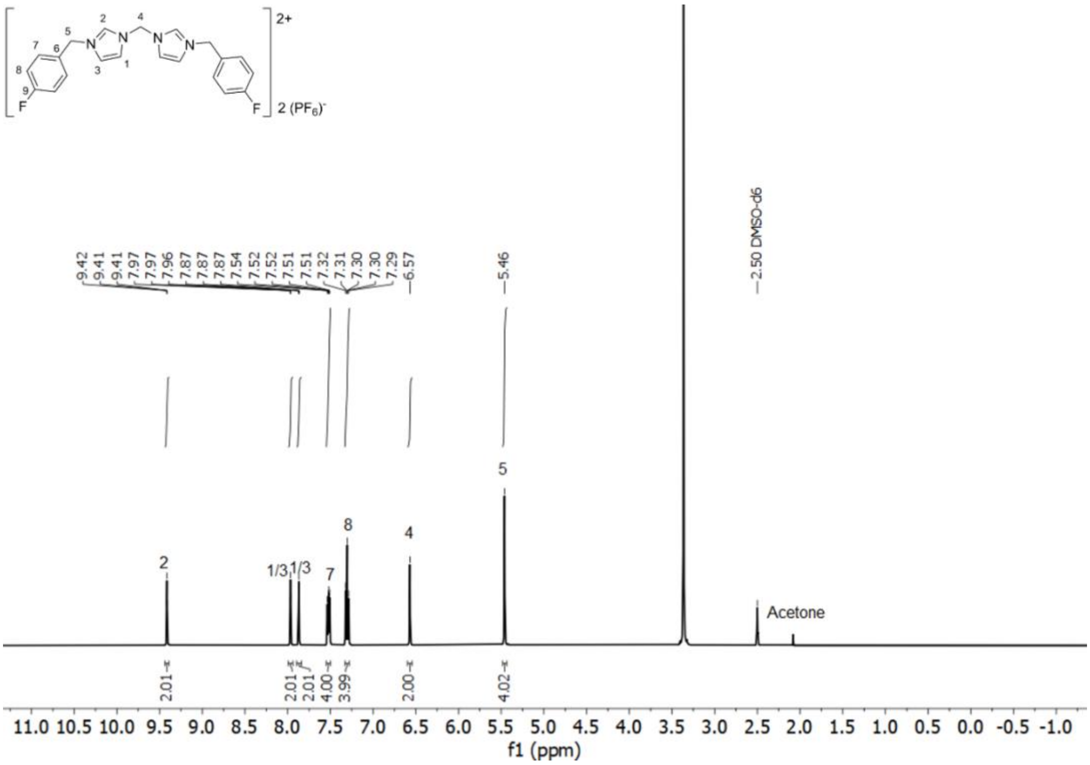

Figure S 16: ^1H NMR spectrum of **$H_2L3(PF_6)_2$** (400 MHz, $(CD_3)_2SO$, 298 K)

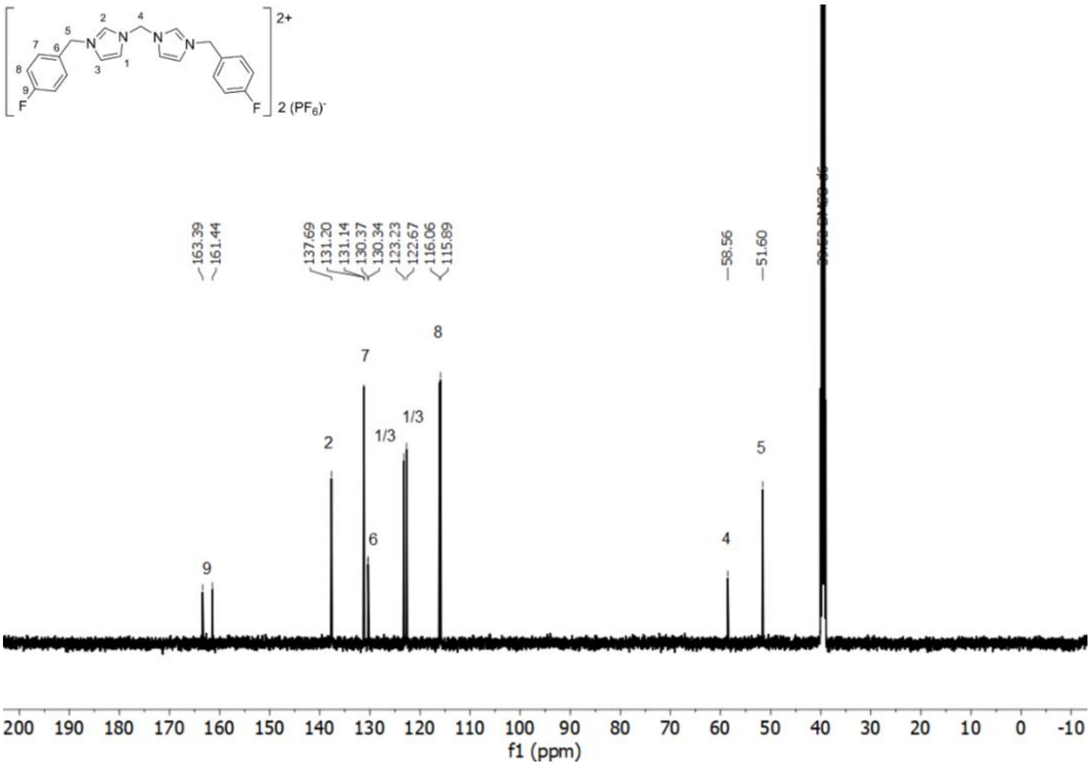

Figure S 17: $^{13}C\{^1H\}$ NMR spectrum of **$H_2L3(PF_6)_2$** (101 MHz, $(CD_3)_2SO$, 298 K)

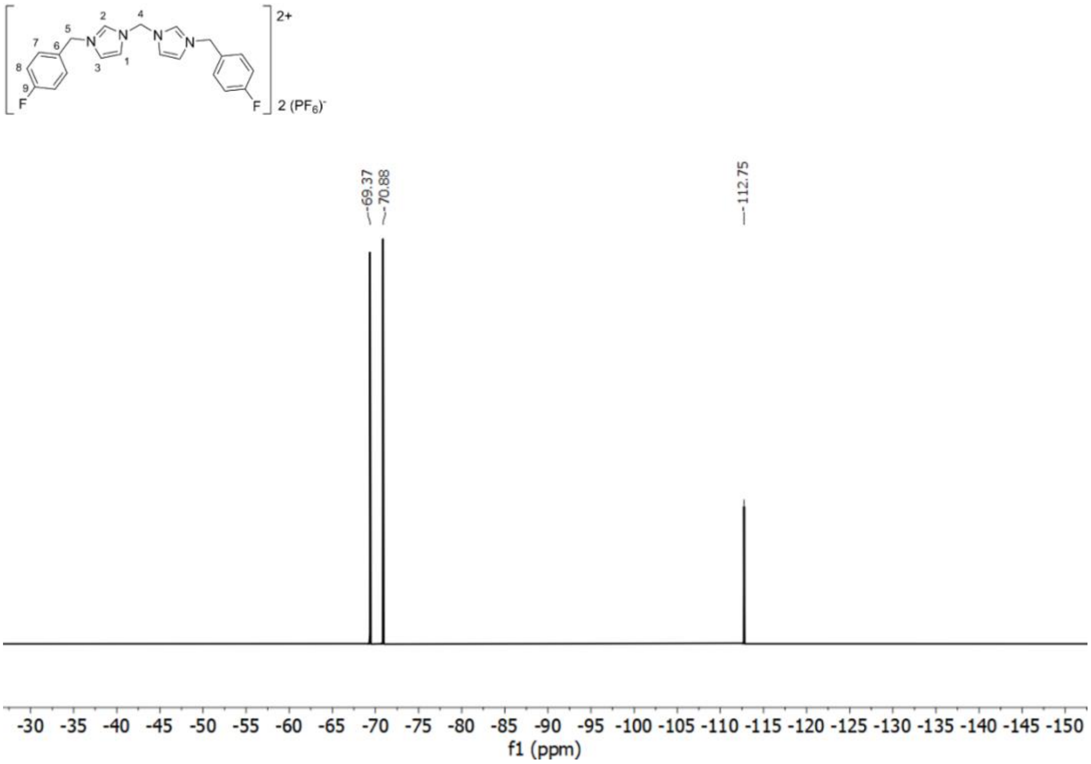

Figure S 18: $^{19}F\{^1H\}$ NMR spectrum of **$H_2L3(PF_6)_2$** (471 MHz, $(CD_3)_2SO$, 298 K)

$[Ag_2(L3)_2](PF_6)_2$

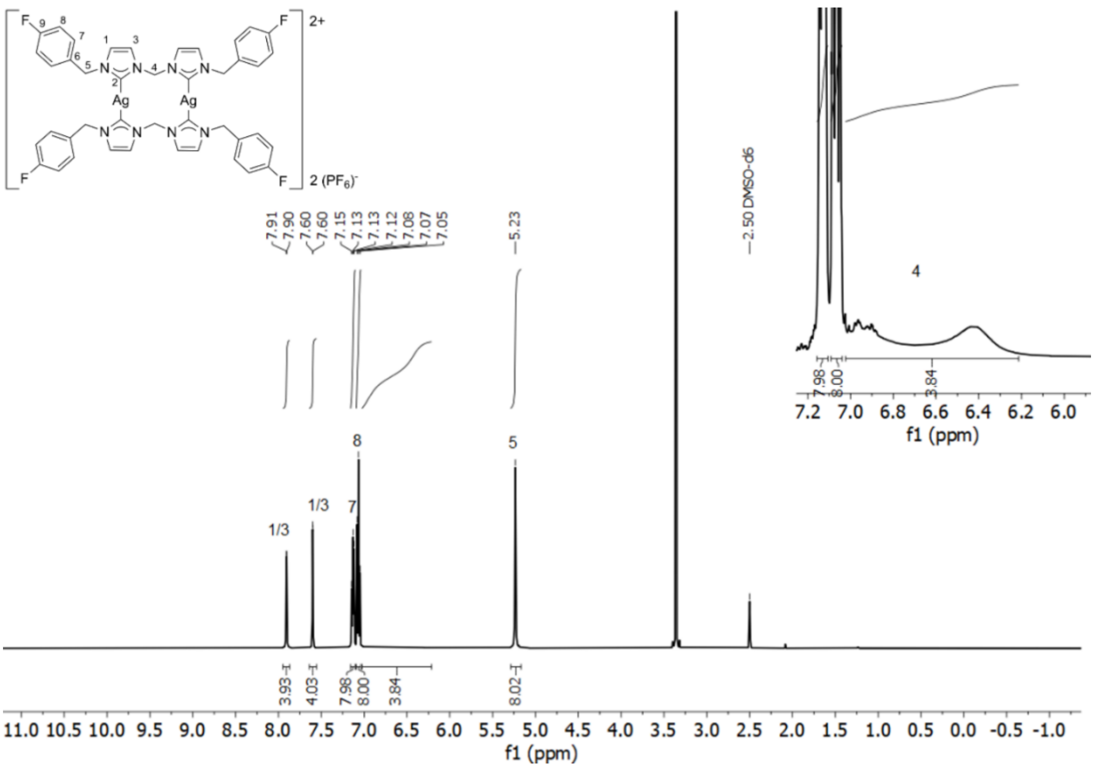

Figure S 19: ^{1}H NMR spectrum of **$[Ag_2(L3)_2](PF_6)_2$** (500 MHz, $(CD_3)_2SO$, 298 K)

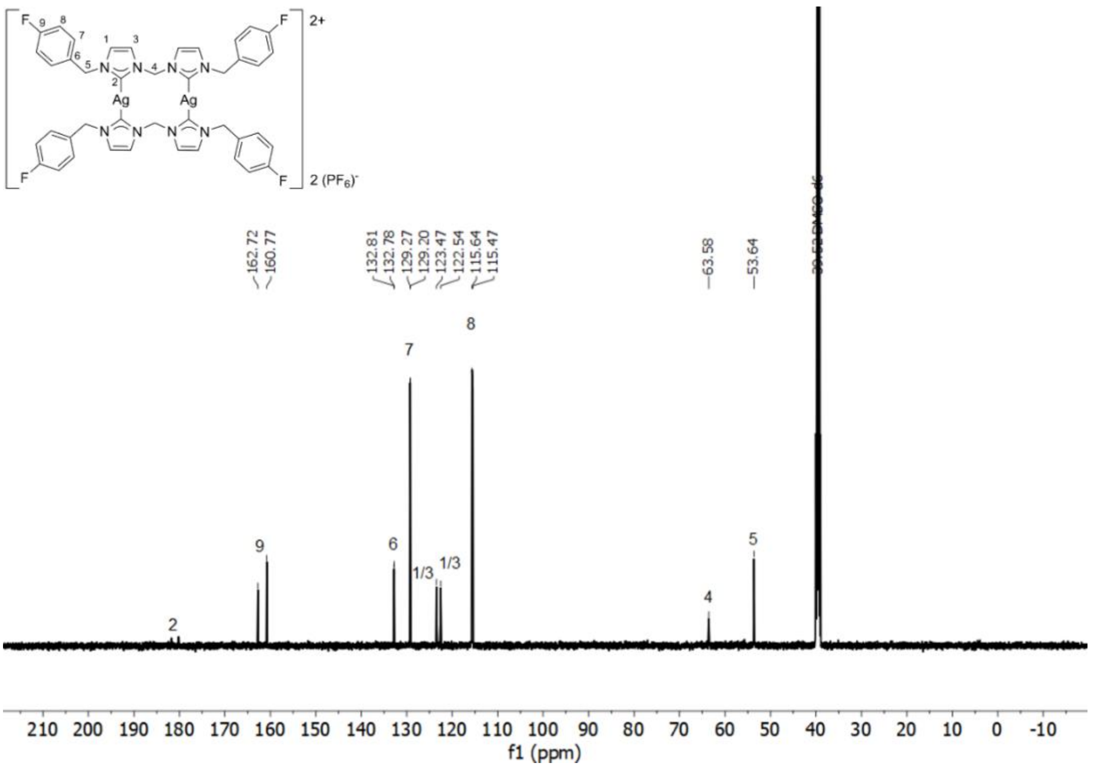

Figure S 20: $^{13}C\{^{1}H\}$ NMR spectrum of **$[Ag_2(L3)_2](PF_6)_2$** (126 MHz, $(CD_3)_2SO$, 298 K)

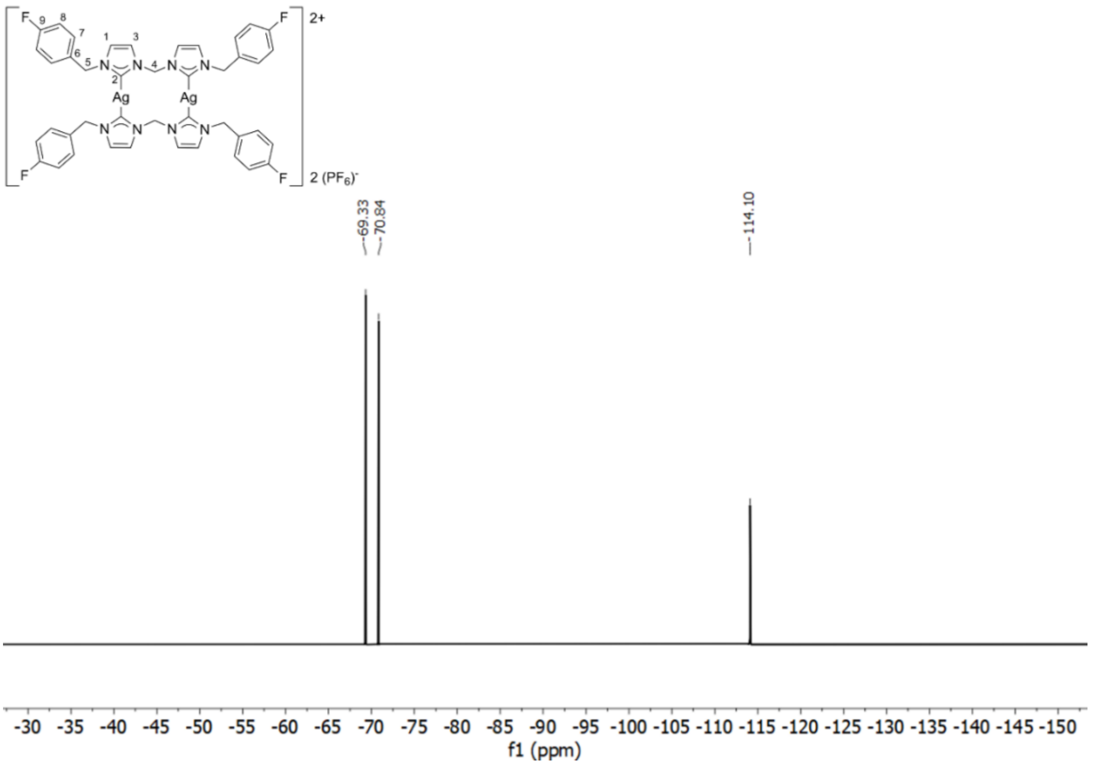

Figure S 21: $^{19}F\{^{1}H\}$ NMR spectrum of **$[Ag_2(L3)_2](PF_6)_2$** (471 MHz, $(CD_3)_2SO$, 298 K)

1,1′-Bis-(4-fluorobenzyl)-3,3′-methylenedibenzimidazolium dibromide – $H_2L4(Br)_2$

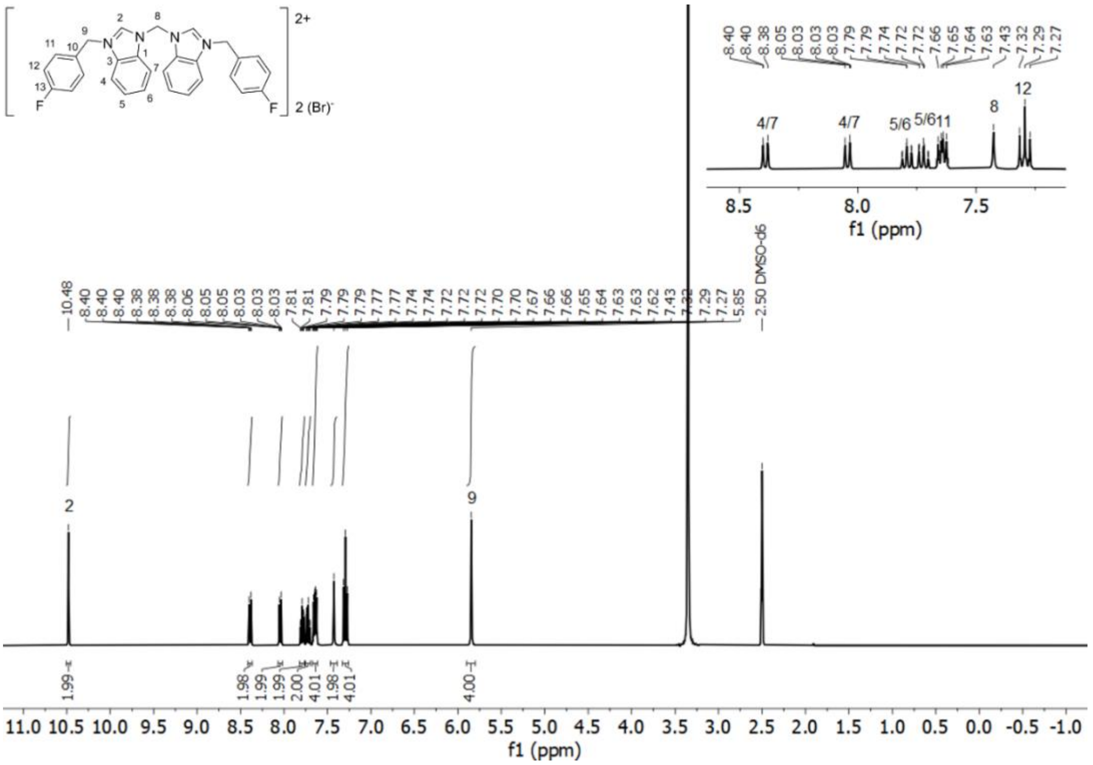

Figure S 22: ^{1}H NMR spectrum of **$H_2L4(Br)_2$** (400 MHz, $(CD_3)_2SO$, 298 K)

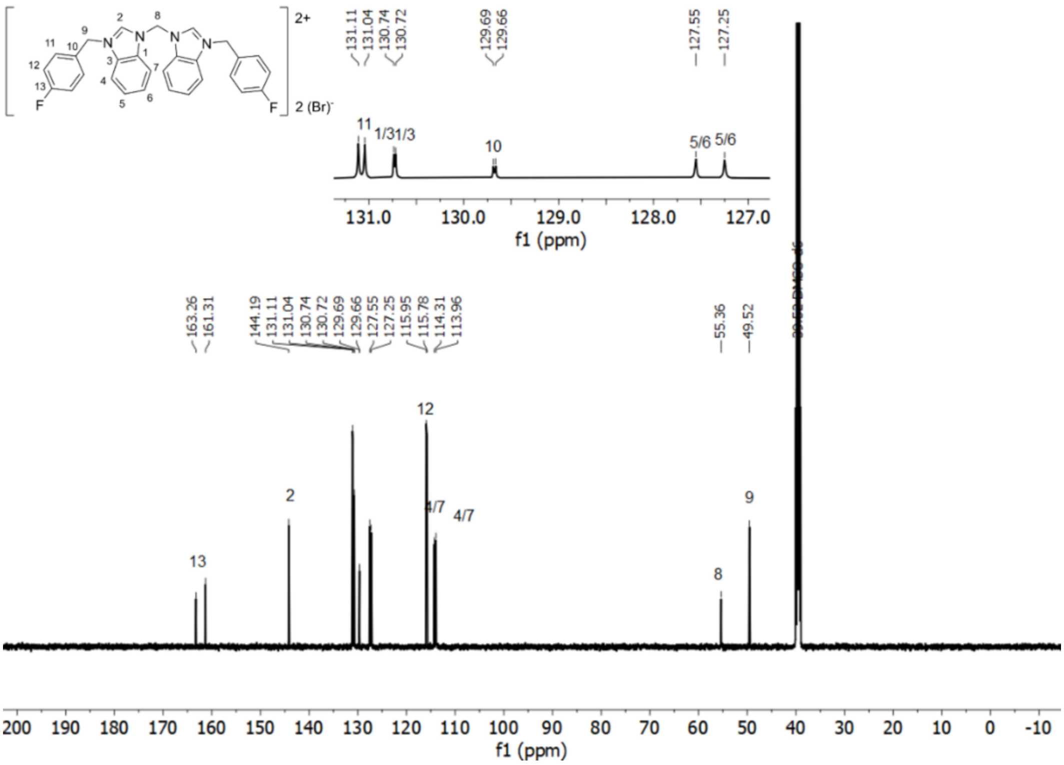

Figure S 23: $^{13}C\{^{1}H\}$ NMR spectrum of $\mathbf{H_2L4(Br)_2}$ (126 MHz, $(CD_3)_2SO$, 298 K)

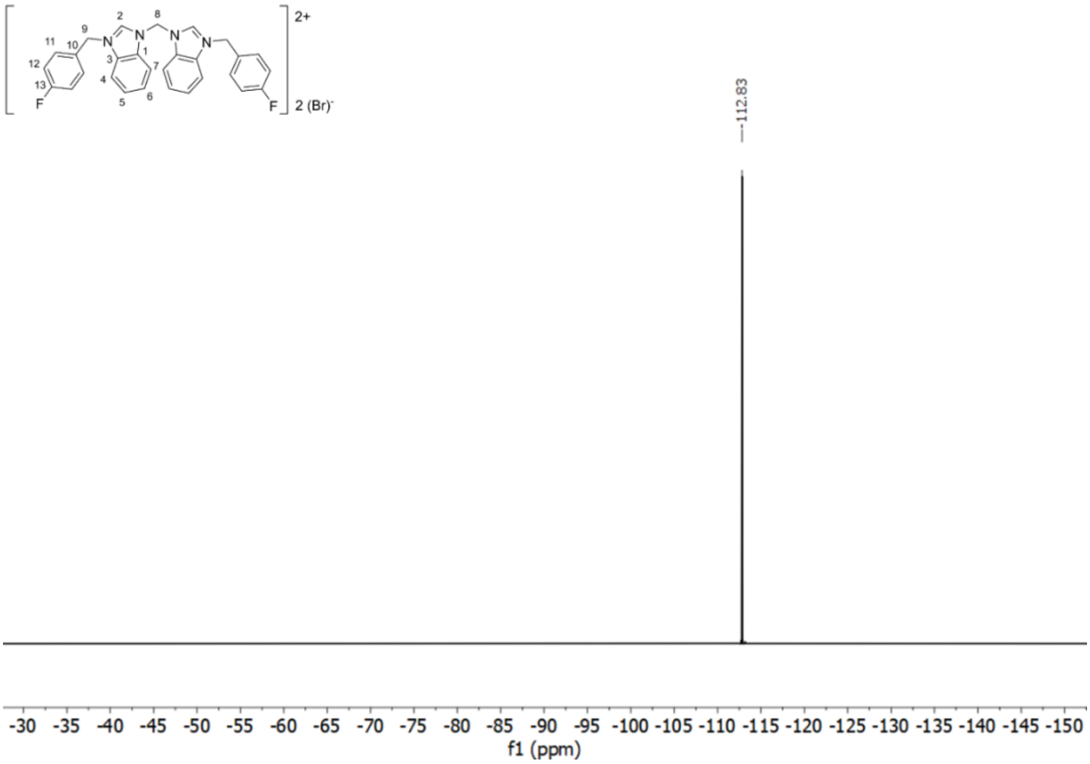

Figure S 24: $^{19}F\{^{1}H\}$ NMR spectrum of $\mathbf{H_2L4(Br)_2}$ (471 MHz, $(CD_3)_2SO$, 298 K)

1,1′-Bis-(4-fluorobenzyl)-3,3′-methylenedibenzimidazolium dihexafluorophosphate – $H_2L4(PF_6)_2$

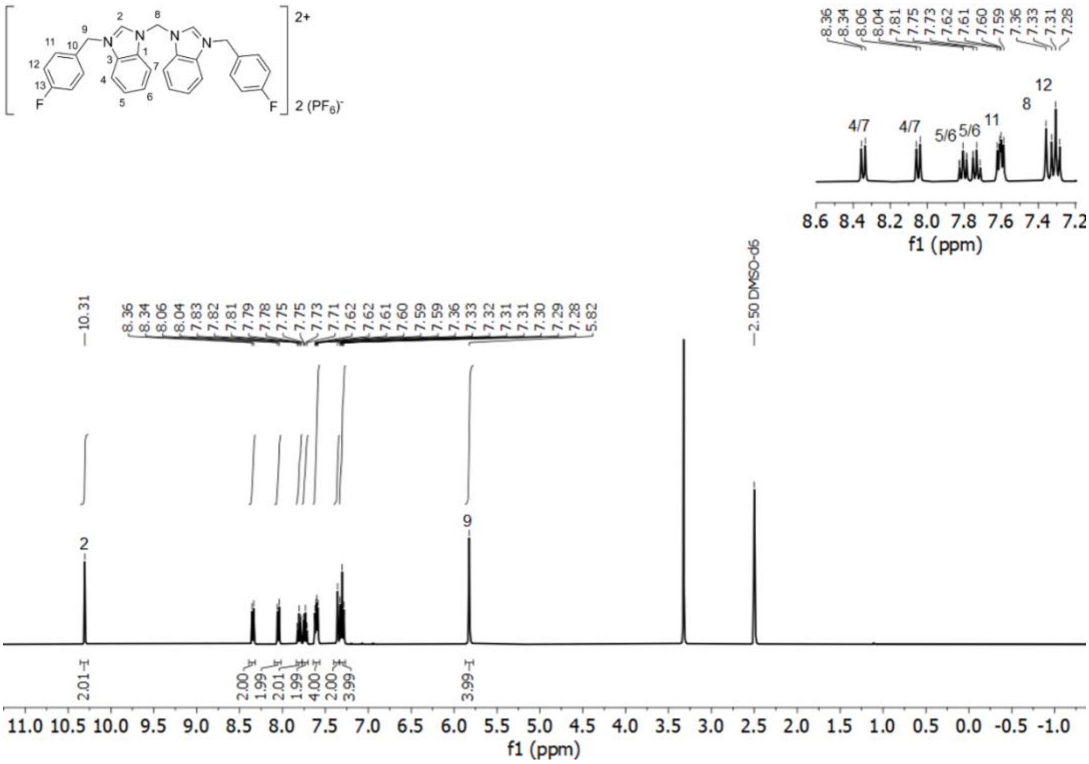

Figure S 25: ^{1}H NMR spectrum of $\mathbf{H_2L4(PF_6)_2}$ (400 MHz, $(CD_3)_2SO$, 298 K)

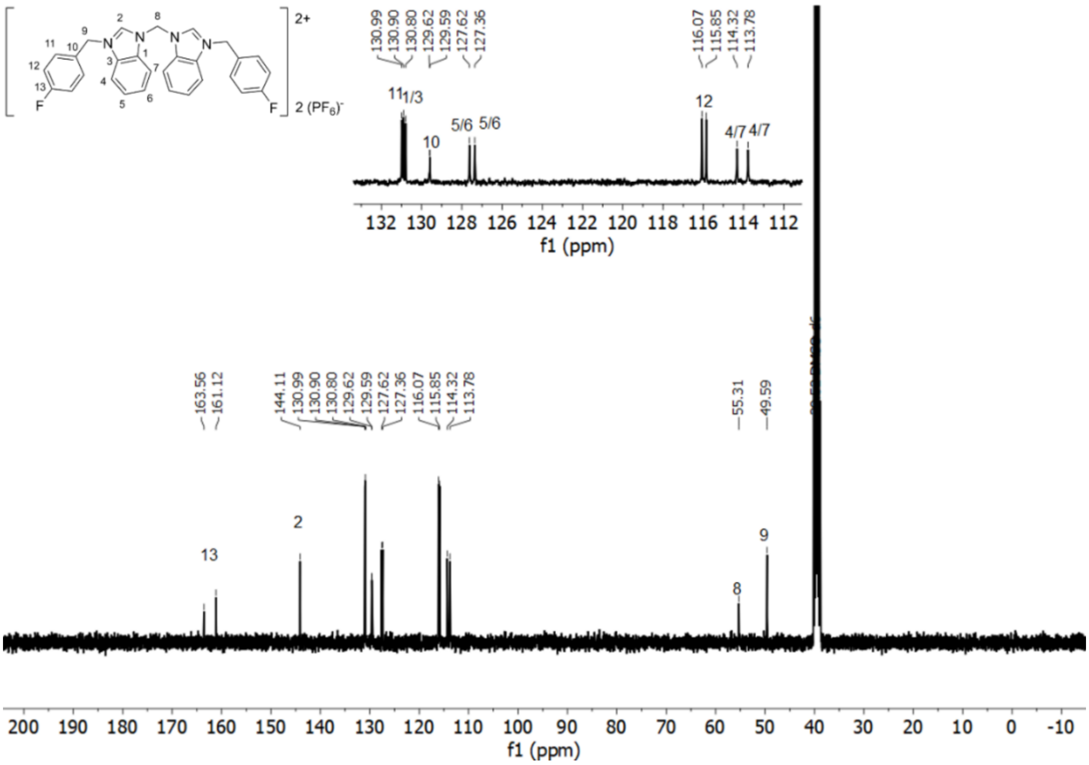

Figure S 26: $^{13}C\{^{1}H\}$ NMR spectrum of $\mathbf{H_2L4(PF_6)_2}$ (101 MHz, $(CD_3)_2SO$, 298 K)

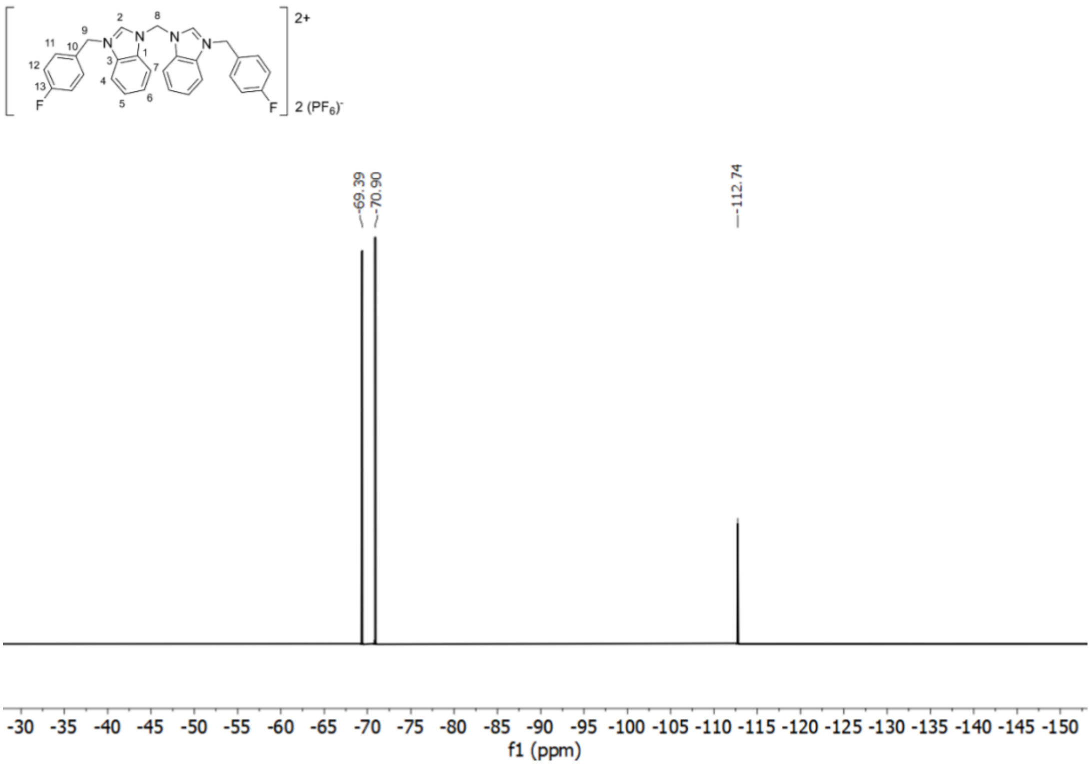

Figure S 27: $^{19}F\{^{1}H\}$ NMR spectrum of **$H_2L4(PF_6)_2$** (471 MHz, $(CD_3)_2SO$, 298 K)

$[Ag_2(L4)_2](PF_6)_2$

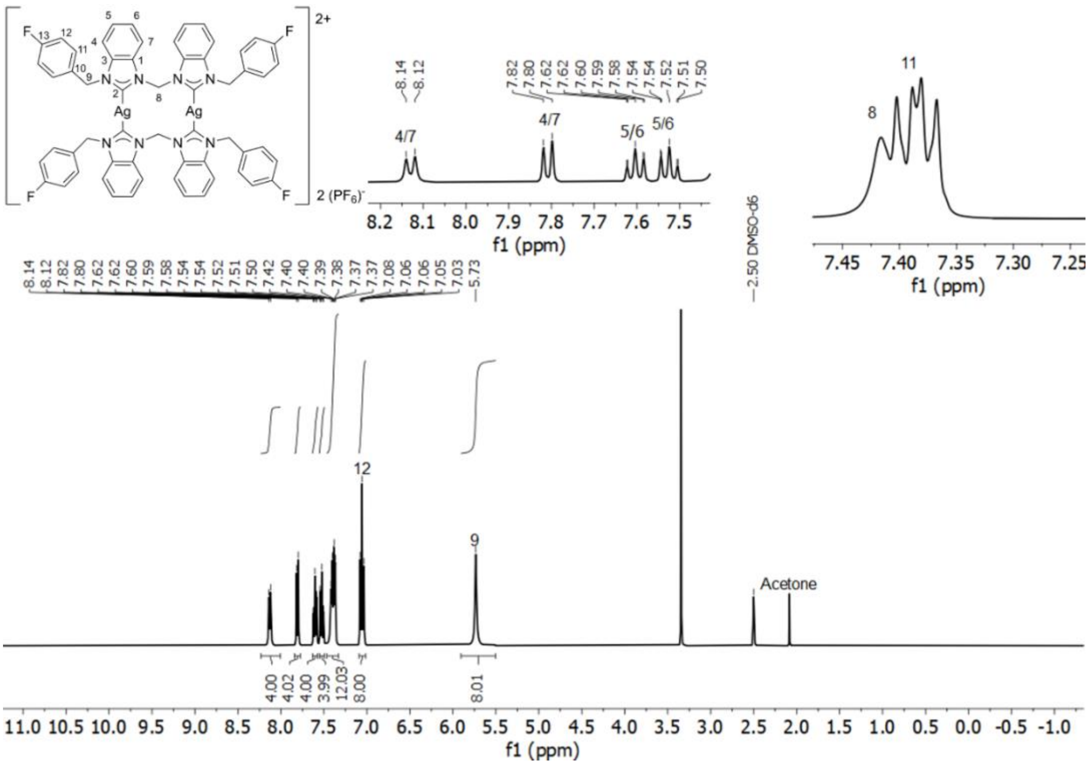

Figure S 28: ^{1}H NMR spectrum of **$[Ag_2(L4)_2](PF_6)_2$** (400 MHz, $(CD_3)_2SO$, 298 K)

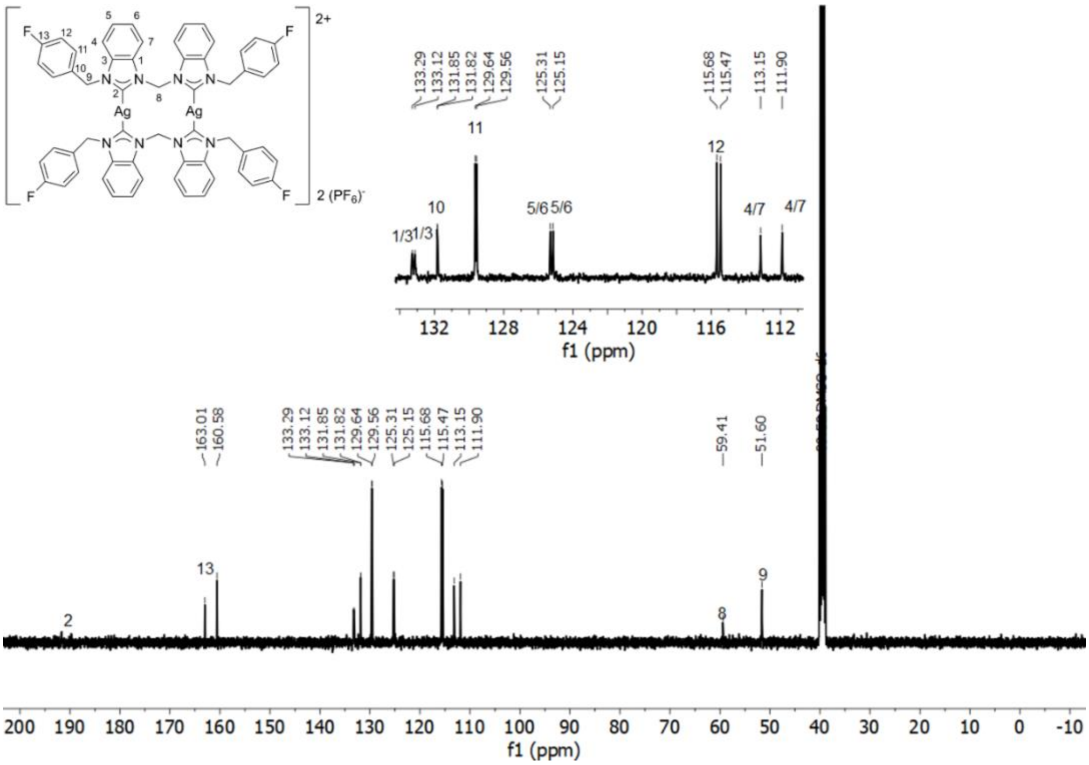

Figure S 29: $^{13}C\{^1H\}$ NMR spectrum of **$[Ag_2(L4)_2](PF_6)_2$** (101 MHz, $(CD_3)_2SO$, 298 K)

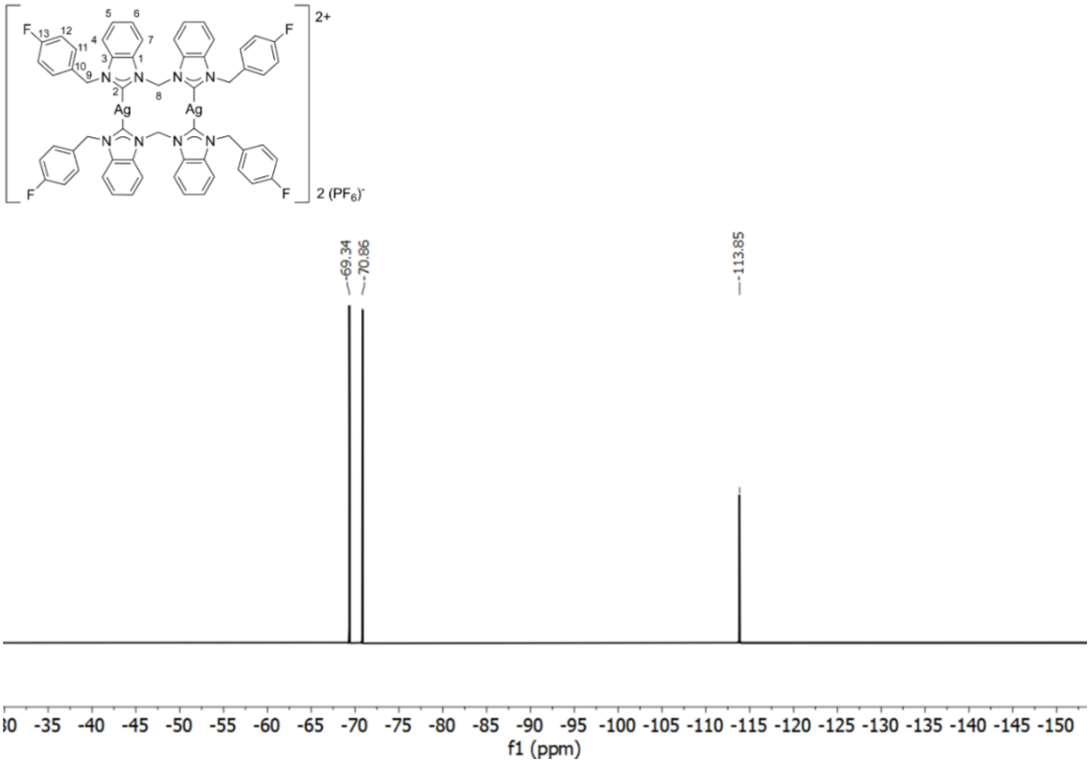

Figure S 30: $^{19}F\{^1H\}$ NMR spectrum of **$[Ag_2(L4)_2](PF_6)_2$** (471 MHz, $(CD_3)_2SO$, 298 K)

1,1′-Bis-(benzyl)-3,3′-methylenediimidazolium dibromide – $H_2L5(Br)_2$

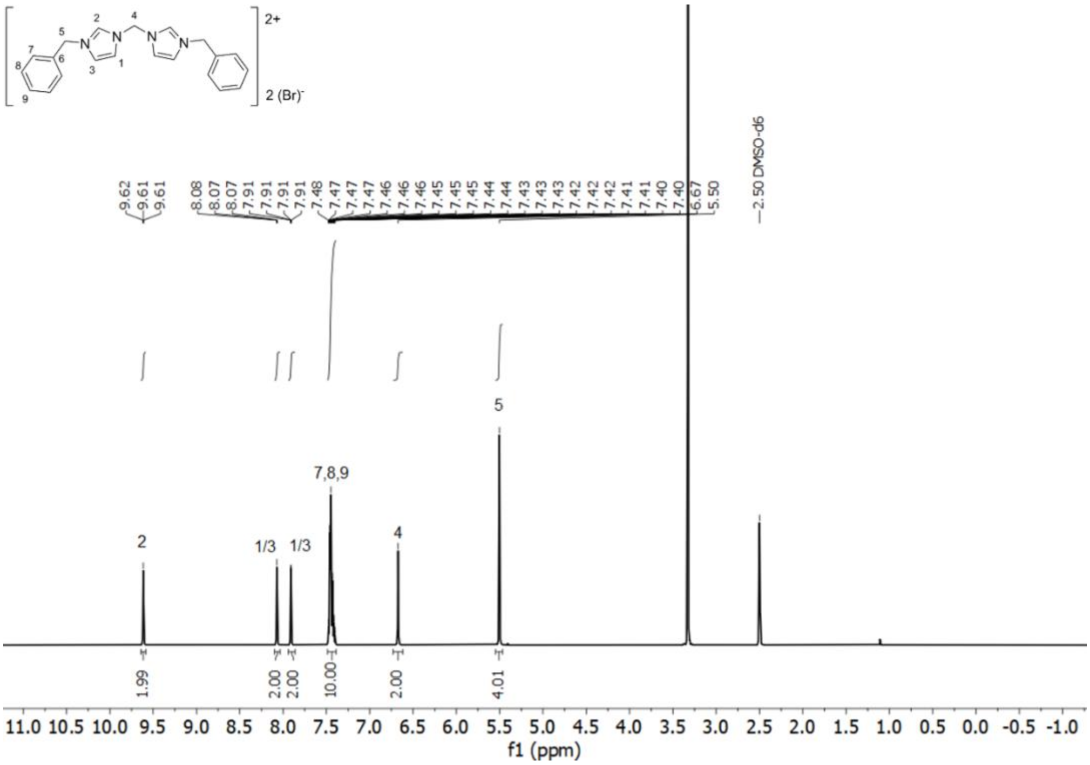

Figure S 31: ^{1}H NMR spectrum of **$H_2L5(Br)_2$** (101 MHz, $(CD_3)_2SO$, 298 K)

1,1′-Bis-(benzyl)-3,3′-methylenediimidazolium dihexafluorophosphate – $H_2L5(PF_6)_2$

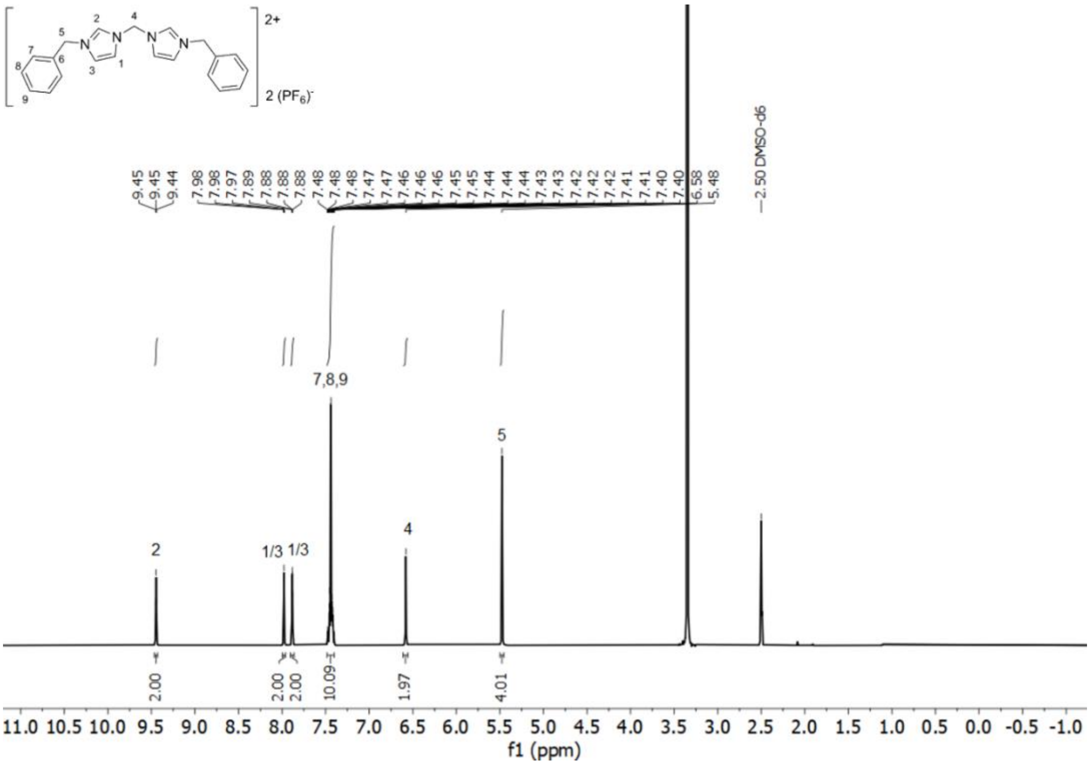

Figure S 32: ^{1}H NMR spectrum of **$H_2L5(PF_6)_2$** (101 MHz, $(CD_3)_2SO$, 298 K)

$[Ag_2(L5)_2](PF_6)_2$

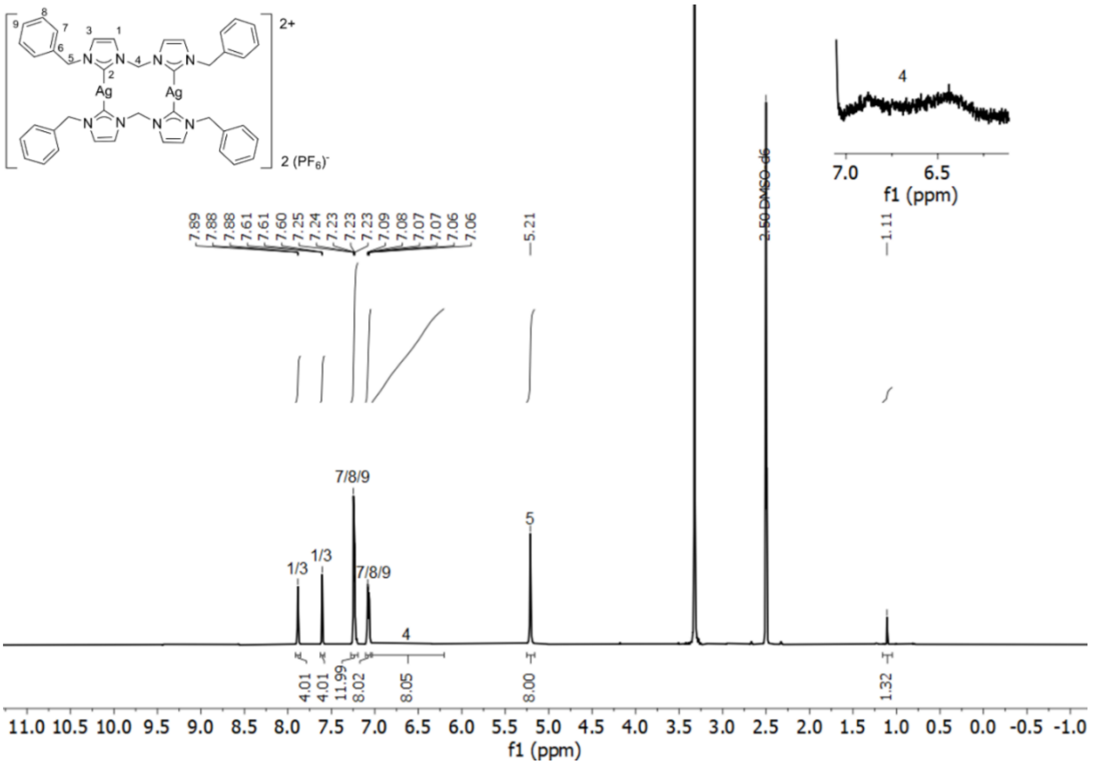

Figure S 33: ^{1}H NMR spectrum of **$[Ag_2(L5)_2](Br)_2$** (101 MHz, $(CD_3)_2SO$, 298 K)

ATR-FTIR Spectroscopy

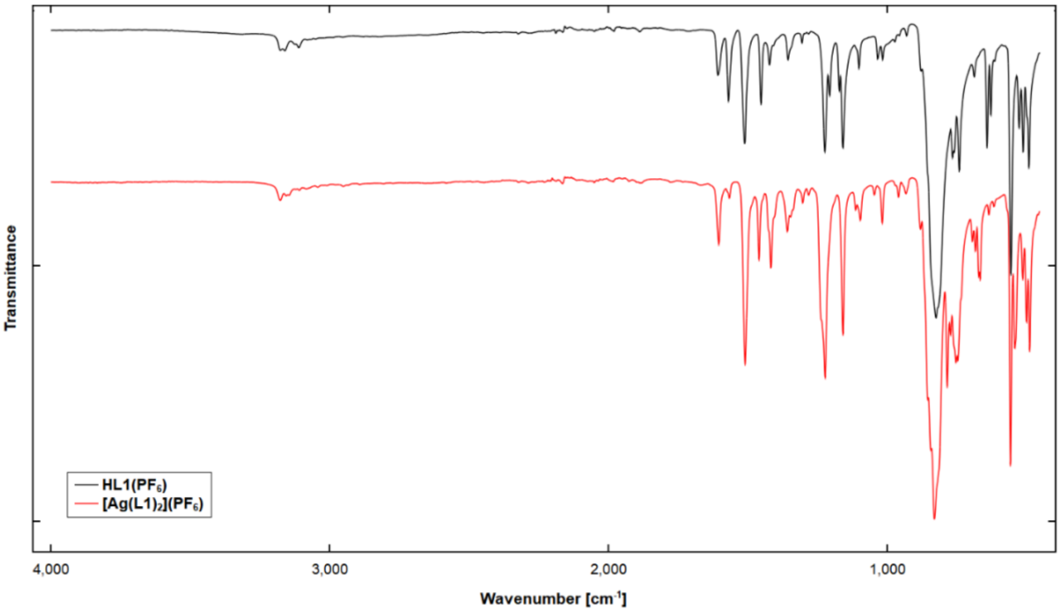

Figure S 34: ATR-FTIR spectrum of **$HL1(PF_6)$** (black) and **$[Ag(L1)_2](PF_6)$** (red) between 450 – 4000 cm^{-1}

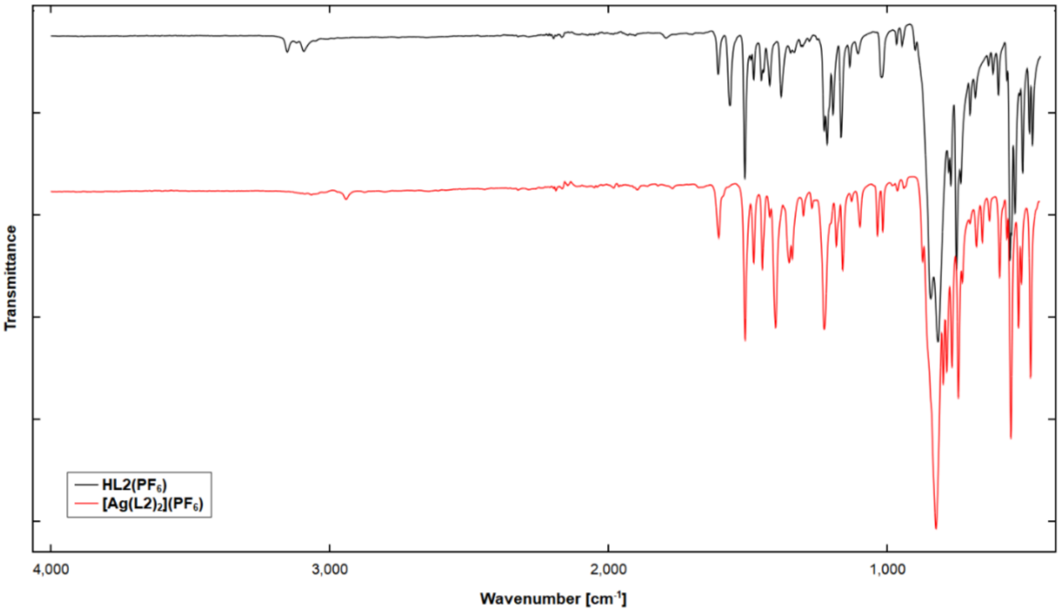

Figure S 35: ATR-FTIR spectrum of **HL2(PF$_6$)** (black) and **[Ag(L2)$_2$](PF$_6$)$_2$** (red) between 450 – 4000 cm^{-1}

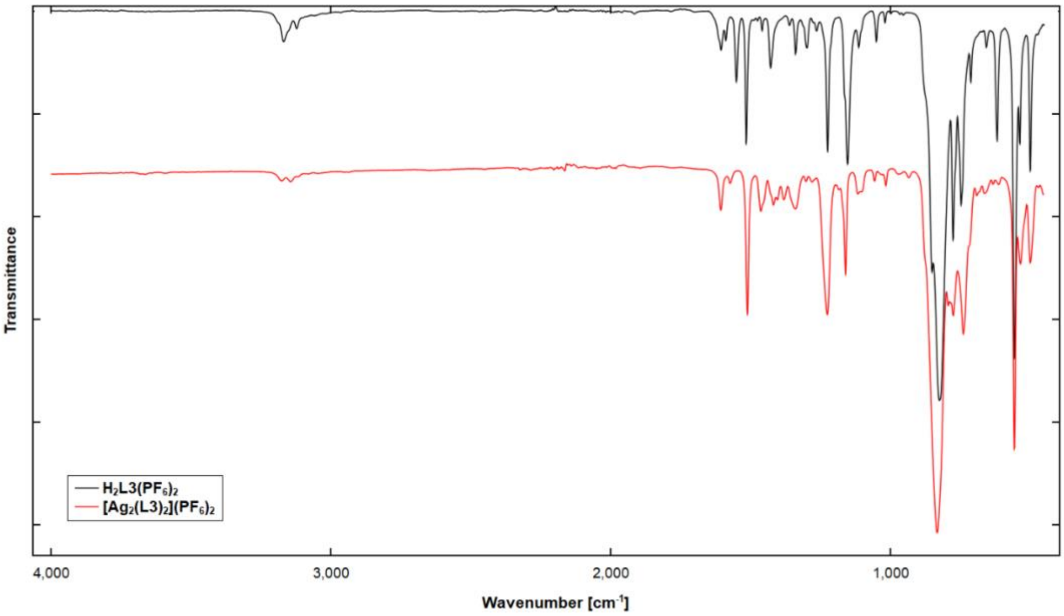

Figure S 36: ATR-FTIR spectrum of **H$_2$L3(PF$_6$)$_2$** (black) and **[Ag$_2$(L3)$_2$](PF$_6$)$_2$** (red) between 450 – 4000 cm^{-1}

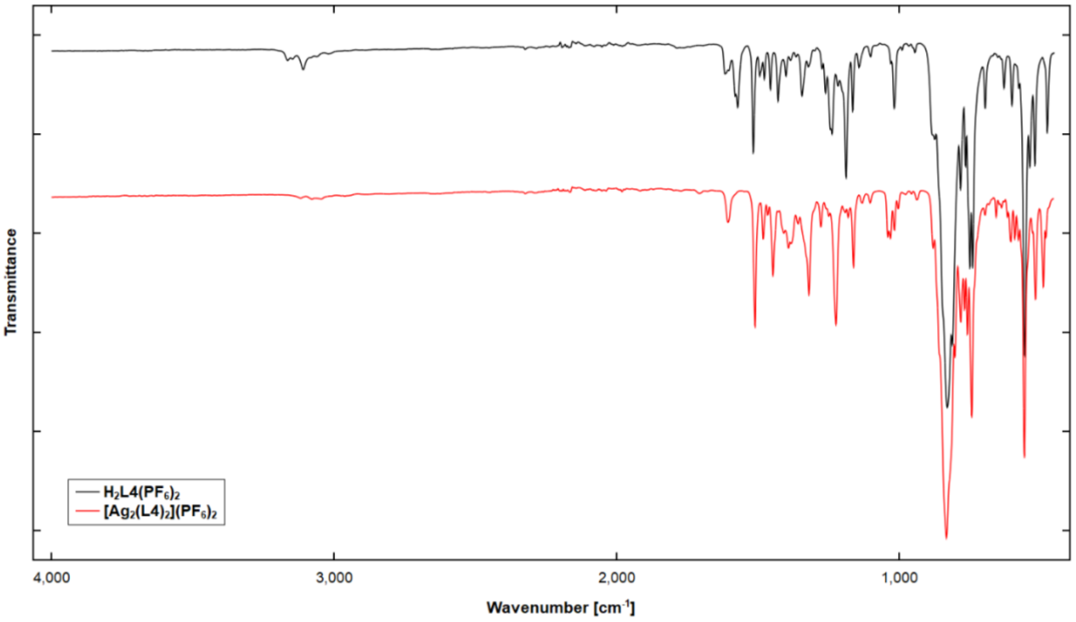

Figure S 37: ATR-FTIR spectrum of **$H_2L4(PF_6)_2$** (black) and **$[Ag_2(L4)_2](PF_6)_2$** (red) between 450 – 4000 cm^{-1}

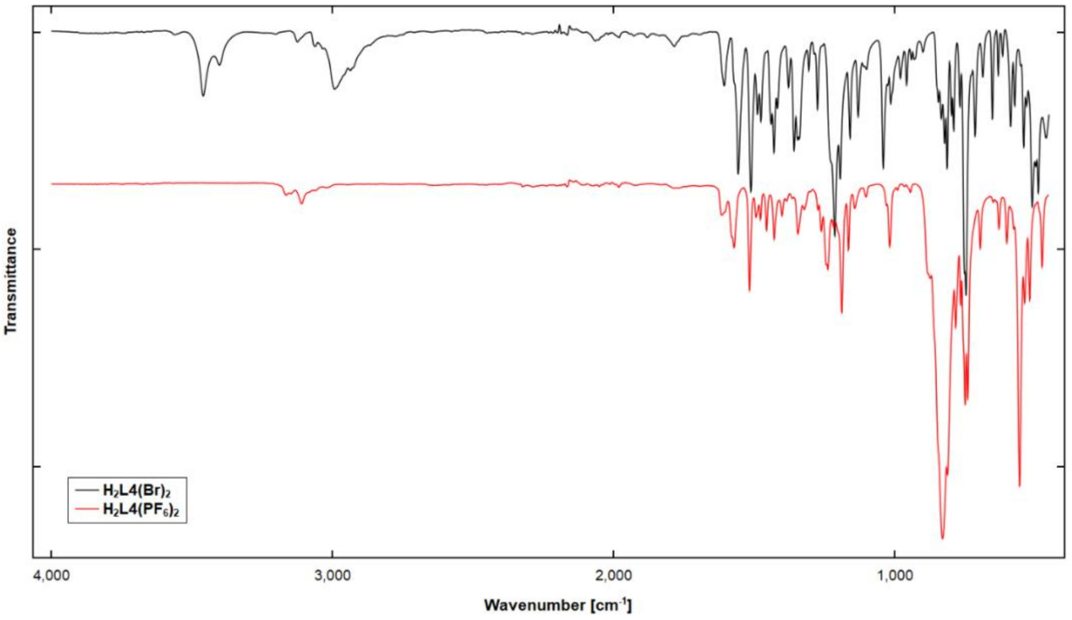

Figure S 38: ATR-FTIR spectra comparing **$H_2L4(Br)_2$** (red) with **$[Ag_2(L4)_2](PF_6)_2$** (black) between 450 – 4000 cm^{-1}

Single Crystal X-ray Diffraction

Table S 1: scXRD data for complexes **[Ag(L2)$_2$](PF$_6$)**, **[Ag$_2$(L3)$_2$](PF$_6$)$_2$**, **[Ag$_2$(L4)$_2$](PF$_6$)$_2$** and **[Ag$_2$(L4)$_2$](PF$_6$)$_2$ (2)**, s.u.s shown in parenthesis

Identification code	[Ag(L2)$_2$](PF$_6$)	[Ag$_2$(L3)$_2$](PF$_6$)$_2$	[Ag$_2$(L4)$_2$](PF$_6$)$_2$	[Ag$_2$(L4)$_2$](PF$_6$)$_2$ (2)
**CSD Numbers**	2361333	2361334	2361335	2361336
**Empirical formula**	$C_{42}H_{32}AgF_{10}N_4P$	$C_{50}H_{56}Ag_2F_{16}N_8O_2P_2$	$C_{66}H_{56}Ag_2F_{16}N_{12}P_2$	$C_{58}H_{43}Ag_2F_{16}N_8P_2$
**Formula weight**	921.55	1382.70	1598.90	1433.68
**Temperature/K**	100.00(10)	100.00(18)	100.00(10)	100.00(10)
**Crystal system**	triclinic	triclinic	monoclinic	monoclinic
**Space group**	*P*-1	*P*-1	$P2_1/n$	$P2_1/c$
**a/Å**	8.1718(2)	13.4465(3)	14.9660(4)	12.79457(9)
**b/Å**	10.6325(3)	14.1618(3)	26.2548(7)	21.39106(14)
**c/Å**	11.9432(2)	17.0902(4)	16.7971(4)	20.99689(15)
**α/°**	110.163(2)	83.345(2)	90	90
**β/°**	94.565(2)	82.594(2)	93.534(2)	104.3716(7)
**γ/°**	98.557(2)	61.905(2)	90	90
**Volume/Å^3**	953.64(4)	2841.33(12)	6587.5(3)	5566.79(7)
**Z**	1	2	4	4
**ρ_{calc}g/cm^3**	1.605	1.616	1.612	1.711
**μ/mm^{-1}**	0.657	0.844	0.740	7.096
**F(000)**	464.0	1392.0	3216.0	2860.0
**Crystal size/mm^3**	0.517 × 0.286 × 0.126	0.279 × 0.144 × 0.054	0.337 × 0.159 × 0.131	0.326 × 0.118 × 0.085
**Radiation**	Mo Kα (λ = 0.71073)	Mo Kα (λ = 0.71073)	Mo Kα (λ = 0.71073)	Cu Kα (λ = 1.54184)
**2Θ range for data collection/°**	3.67 to 61.692	3.934 to 61.906	4.07 to 61.856	5.996 to 154.146
**Index ranges**	-10 ≤ h ≤ 11, -13 ≤ k ≤ 13, -15 ≤ l ≤ 16	-17 ≤ h ≤ 18, -20 ≤ k ≤ 19, -24 ≤ l ≤ 24	-20 ≤ h ≤ 18, -36 ≤ k ≤ 31, -20 ≤ l ≤ 22	-14 ≤ h ≤ 16, -26 ≤ k ≤ 23, -26 ≤ l ≤ 23
**Reflections collected**	14570	44077	39379	44896
**Independent reflections**	4777 [R_{int} = 0.0334, R_{sigma} = 0.0297]	14456 [R_{int} = 0.0599, R_{sigma} = 0.0663]	161322 [R_{int} = 0.0435, R_{sigma} = 0.0595]	10846 [R_{int} = 0.0323, R_{sigma} = 0.0263]
**Data/restraints/parameters**	4777/0/265	14456/0/725	16122/0/887	10846/0/775
**Goodness-of-fit on F^2**	1.031	1.027	1.027	1.048
**Final R indexes [I>=2σ (I)]**	R_1 = 0.0244, wR_2 = 0.0561	R_1 = 0.0500, wR_2 = 0.1046	R_1 = 0.0428, wR_2 = 0.0971	R_1 = 0.0293, wR_2 = 0.0741
**Final R indexes [all data]**	R_1 = 0.0267, wR_2 = 0.0569	R_1 = 0.0794, wR_2 = 0.1138	R_1 = 0.0680, wR_2 = 0.1048	R_1 = 0.0311, wR_2 = 0.0751
**Largest diff. peak/hole / e Å^{-3}**	0.52/-0.46	1.84/-0.70	0.86/-0.58	0.96/-0.86

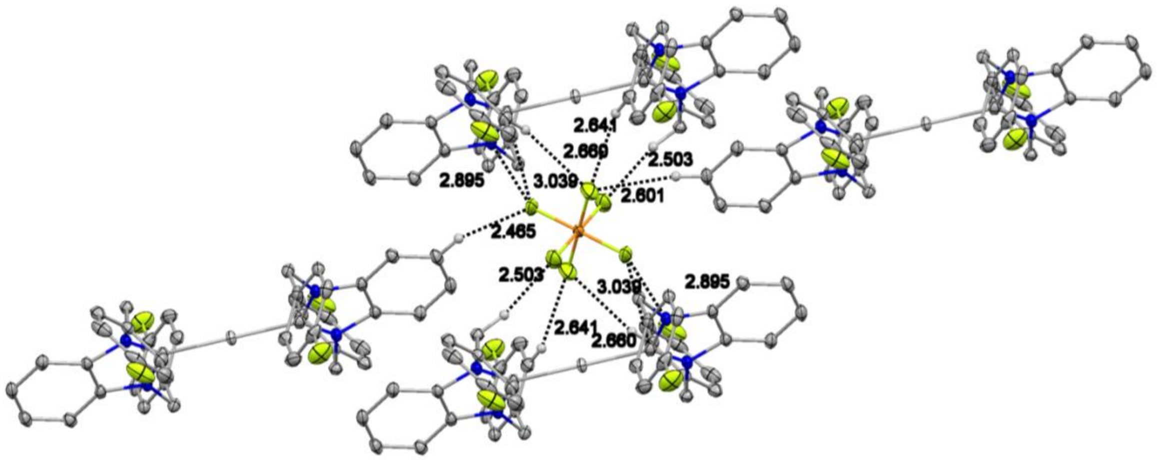

Figure S 39: Packing diagram of **$[Ag_2(L2)_2](PF_6)$** showing the interactions between the Ag(I)-NHC complex and the PF_6 counterion.

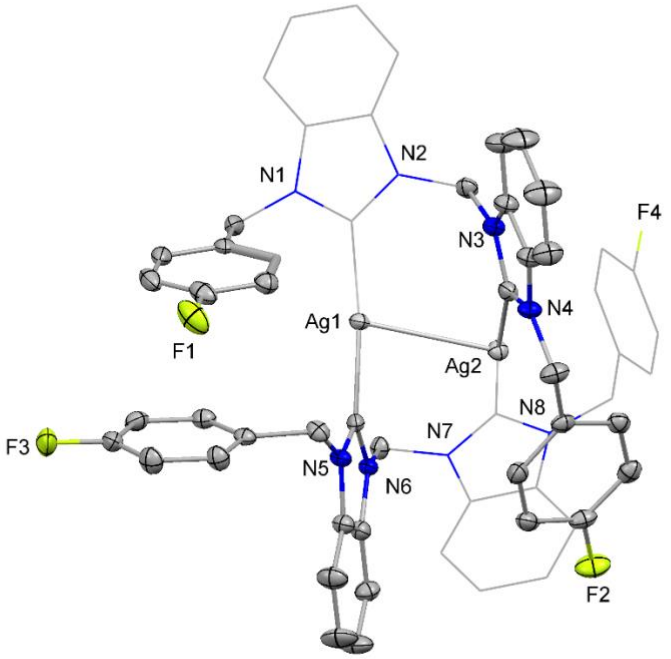

Figure S 40: Molecular structures of **$[Ag_2(L4)_2(PF_6)_2$ (2)**. All counterions and solvent molecules are eliminated for clarity, and ellipsoids are placed at 50% probability level.

Table S 2: Selected bond lengths (Å) and bond angles (°) for **[$Ag_2(L4)_2$](PF_6)$_2$ (2)** (s.u.s shown in parenthesis)

Compound	**[Ag(L4)$_2$](PF_6)$_2$ (2)**
	**Bond Lengths (Å)**
Ag(1)-$C_{carbene}$	2.087(2)/ 2.087(2)
Ag(2)-$C_{carbene}$	2.106(2)/ 2.103(2)
N-$C_{carbene}$ [Ag(1)]	1.353(3)/ 1.354(3)
	1.343(3)/ 1.360(3)
N-$C_{carbene}$ [Ag(2)]	1.362(3)/ 1.349(3)
	1.361(3)/ 1.349(3)
Ag(1)-Ag(2)	2.9874(2)
	**Bond Angles (°)**
$C_{carbene}$-Ag-$C_{carbene}$	170.09(9)/ 174.64(9)
N-$C_{carbene}$-N [Ag(1)]	106.0(2)/ 105.89(19)
N-$C_{carbene}$-N [Ag(2)]	105.7(2)/ 105.8(2)
	**Torsion Angles (°)**
$C_{carbene}$-Ag(1)-Ag(2)-$C_{carbene}$	-131.46/ -122.69

Cytotoxicity Results

Table S 3: IC_{50} values ± SD (n = 9) for CDDP, Tam, ligands **HL1(PF_6)**, **HL2(PF_6), H_2L3(PF_6)$_2$, H_2L4(PF_6)$_2$**, **H_2L5(PF_6)$_2$** and silver complexes **[Ag(L1)$_2$](PF_6), [Ag(L2)$_2$](PF_6), [Ag_2(L3)$_2$](PF_6)$_2$, [Ag_2(L4)$_2$](PF_6)$_2$**, **[Ag_2(L5)$_2$](PF_6)$_2$** when tested agsinst MDA-MB-231, MCF-7, and ARPE-19. Selectivity ratio (SR) values of MCF-7 versus MDA-MB-231 are also stated (ND = not determined).

Compounds	MDA-MB-231	MCF-7	ARPE-19	MCF-7/MDA-MB-231 SR
**CDDP**	> 100	54 ± 3	> 100	0.54
**Tam**	39.59 ± 0.06	36 ± 1	75 ± 1	0.91
**HL1(PF_6)**	> 100	> 100	> 100	ND
**[Ag(L1)$_2$](PF_6)**	10.8 ± 0.2	20.7 ± 0.4	24.7 ± 0.1	1.92
**HL2(PF_6)**	42.6 ± 0.5	> 100	> 100	2.35
**[Ag(L2)$_2$](PF_6)**	3.7 ± 0.3	5.0 ± 0.2	15.8 ± 0.5	1.34
**H_2L3(PF_6)**	> 100	> 100	> 100	ND
**[Ag_2(L3)$_2$](PF_6)$_2$**	5.5 ± 0.1	18.3 ± 0.2	37.7 ± 0.8	3.30
**H_2L4(PF_6)**	> 100	> 100	> 100	ND
**[Ag_2(L4)$_2$](PF_6)$_2$**	4.5 ± 0.3	8.5 ± 0.1	32 ± 1	1.91
**H_2L5(PF_6)**	> 100	> 100	> 100	ND
**[Ag_2(L5)$_2$](PF_6)$_2$**	13 ± 1	18.6 ± 0.4	35 ± 2	1.38

Table S 4: Selectivity ratios (SR) when comparing silver complexes **$[Ag(L1)_2](PF_6)$, $[Ag(L2)_2](PF_6)$, $[Ag_2(L3)_2](PF_6)_2$, $[Ag_2(L4)_2](PF_6)_2$, $[Ag_2(L5)_2](PF_6)_2$** with CDDP and Tam. SR values > 1 indicate higher activity of the silver complexes (* indicates a minimum value, as one IC_{50} value is > 100 µM).

Compounds	MDA-MB-231		MCF-7	
	Ag cf. CDDP	Ag cf. Tam	Ag cf. CDDP	Ag cf. Tam
**$[Ag(L1)_2](PF_6)$**	9.24*	3.66	2.63	1.73
**$[Ag(L2)_2](PF_6)$**	26.94*	10.67	10.98	7.25
**$[Ag_2(L3)_2](PF_6)_2$**	18.09*	7.16	2.98	1.97
**$[Ag_2(L3)_2](PF_6)_2$**	22.46*	8.90	6.41	4.23
**$[Ag_2(L3)_2](PF_6)_2$**	7.44	2.95	2.93	1.93

Table S 5: Selectivity index (SI) values of CDDP, Tam, and silver **$[Ag(L1)_2](PF_6)$, $[Ag(L2)_2](PF_6)$, $[Ag_2(L3)_2](PF_6)_2$, $[Ag_2(L4)_2](PF_6)_2$, $[Ag_2(L5)_2](PF_6)_2$** when comparing IC_{50} values against ARPE-19 and each breast cell line. SI values > 1 indicate a selectivity for the breast cancer (* indicates a minimum value, as one IC_{50} value is > 100 µM).

Compounds	MDA-MB-231 vs ARPE-19	MCF-7 vs ARPE-19
**CDDP**	ND	1.84*
**Tam**	1.89	2.08
**$[Ag(L1)_2](PF_6)$**	2.28	1.19
**$[Ag(L2)_2](PF_6)$**	4.25	3.18
**$[Ag_2(L3)_2](PF_6)_2$**	6.83	2.07
**$[Ag_2(L4)_2](PF_6)_2$**	7.11	3.73
**$[Ag_2(L5)_2](PF_6)_2$**	2.61	1.89

Optical Microscope Images

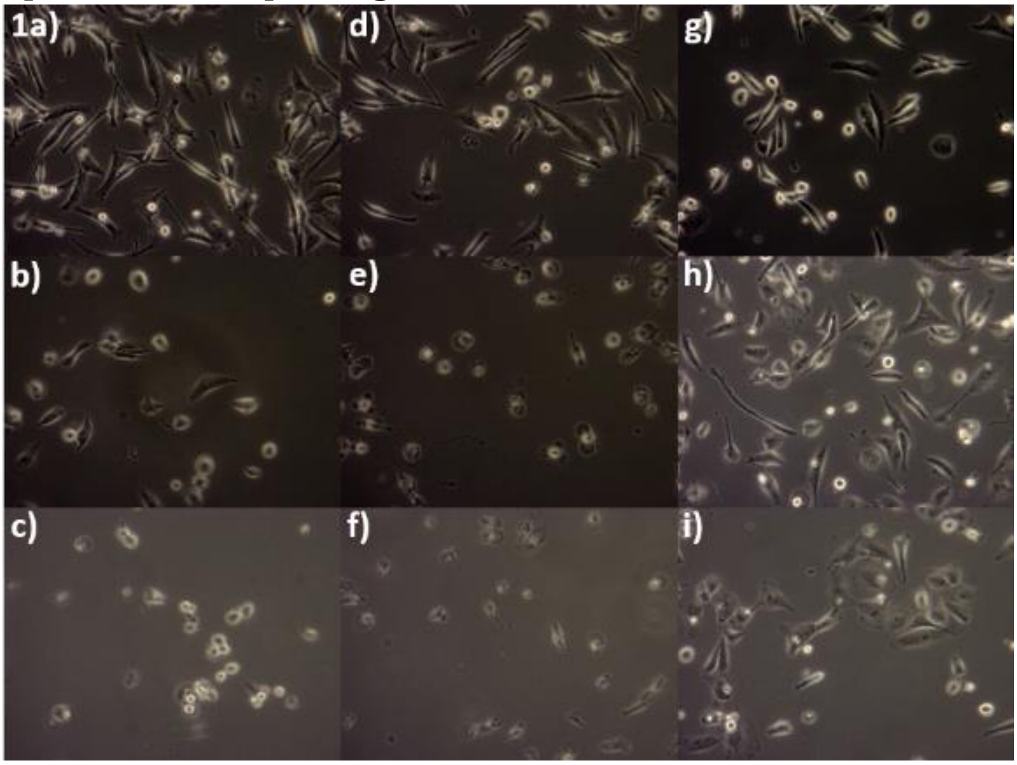

Figure S 41: MDA-MB-231 cells after being treated for 0-4 h with **$[Ag(L1)_2](PF_6)$**. **a)** 10 µM 0 h, **b)** 10 µM 1h, **c)** 10 µM 4h, **d)** 50 µM 0h, **e)** 50 µM 1 h, **f)** 50 µM 4 h, **g)** control (DMSO 0.05%) 0 h, **h)** control (DMSO 0.05%), 1 h, **i)** control (DMSO 0.05%), 4 h.

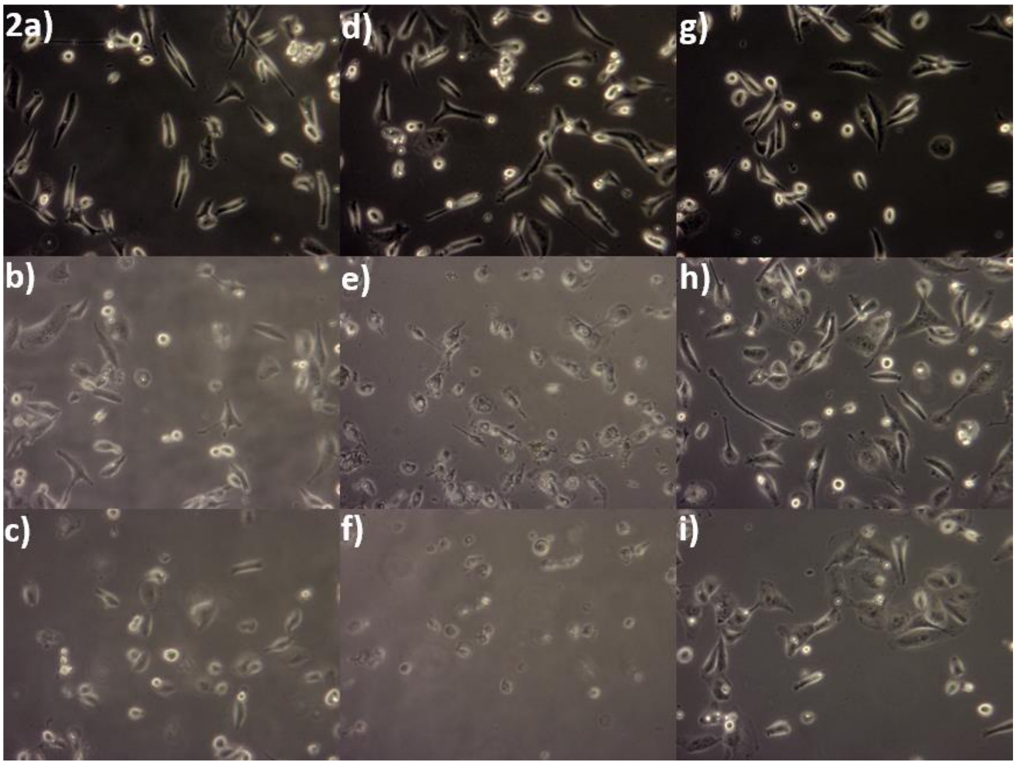

Figure S 42: MDA-MB-231 cells after being treated for 0-4 h with **$[Ag(L2)_2](PF_6)$**. **a)** 10 µM 0 h, **b)** 10 µM 1h, **c)** 10 µM 4h, **d)** 50 µM 0h, **e)** 50 µM 1 h, **f)** 50 µM 4 h, **g)** control (DMSO 0.05%) 0 h, **h)** control (DMSO 0.05%), 1 h, **i)** control (DMSO 0.05%), 4 h.

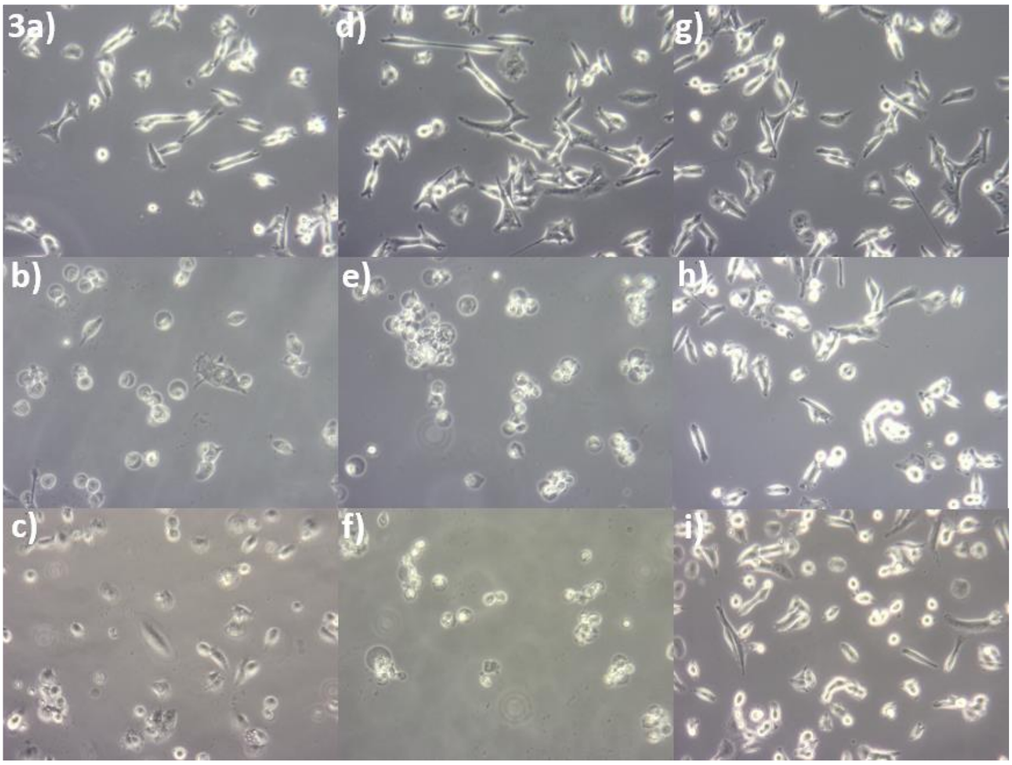

Figure S 43: MDA-MB-231 cells after being treated for 0-4 h with **[$Ag_2(L3)_2$](PF_6). a)** 10 µM 0 h, **b)** 10 µM 1h, **c)** 10 µM 4h, **d)** 50 µM 0h, **e)** 50 µM 1 h, **f)** 50 µM 4 h, **g)** control (DMSO 0.05%) 0 h, **h)** control (DMSO 0.05%), 1 h, **i)** control (DMSO 0.05%), 4 h.

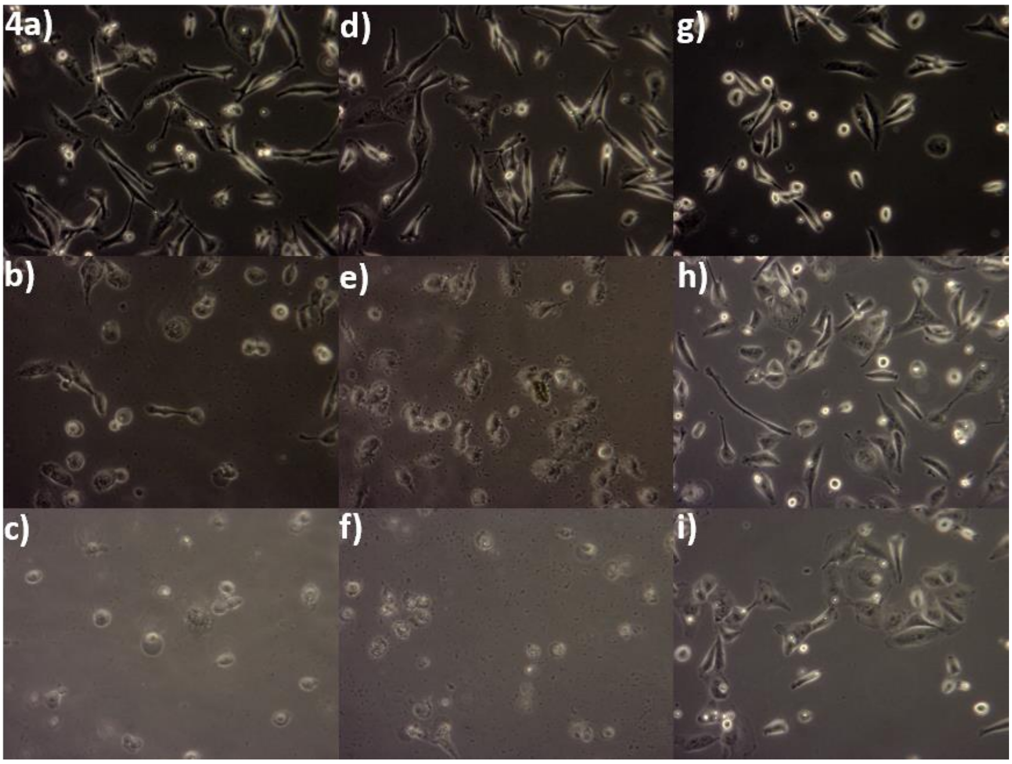

Figure S 44: MDA-MB-231 cells after being treated for 0-4 h with **[$Ag_2(L4)_2$](PF_6). a)** 10 µM 0 h, **b)** 10 µM 1h, **c)** 10 µM 4h, **d)** 50 µM 0h, **e)** 50 µM 1 h, **f)** 50 µM 4 h, **g)** control (DMSO 0.05%) 0 h, **h)** control (DMSO 0.05%), 1 h, **i)** control (DMSO 0.05%), 4 h.

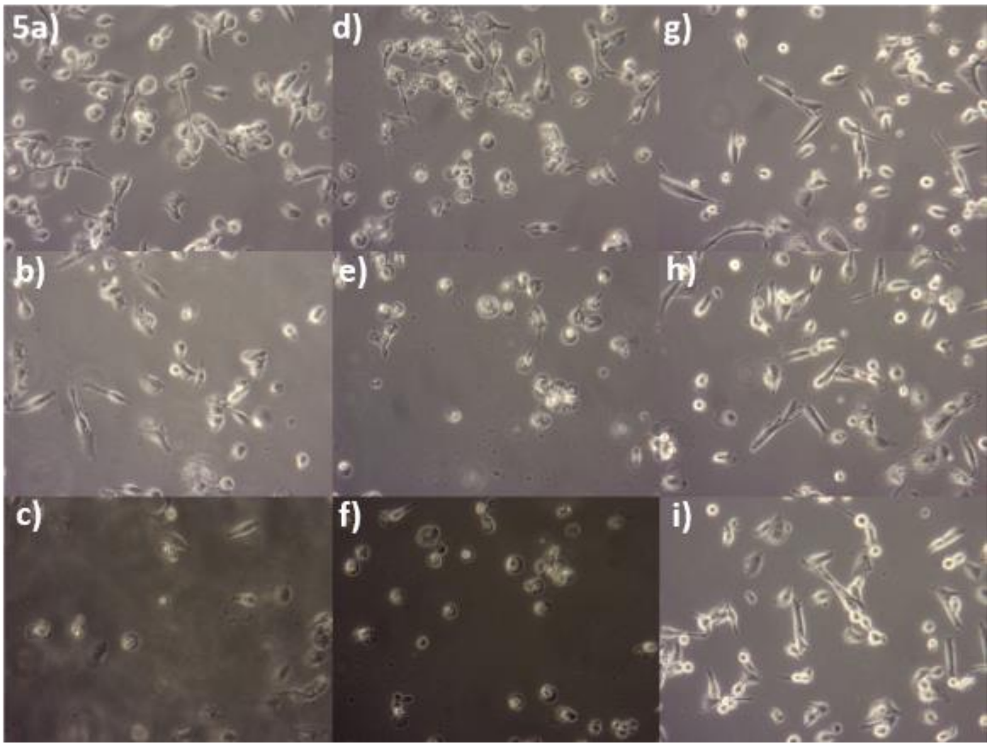

Figure S 45: MDA-MB-231 cells after being treated for 0-4 h with **[$Ag_2(L5)_2$](PF_6).** **a)** 10 µM 30 mins, **b)** 10 µM 1h, **c)** 10 µM 4h, **d)** 50 µM 30 mins, **e)** 50 µM 1 h, **f)** 50 µM 4 h, **g)** control (DMSO 0.05%) 30 mins, **h)** control (DMSO 0.05%), 1 h, **i)** control (DMSO 0.05%), 4 h.

NMR Spectroscopy – Stability Studies

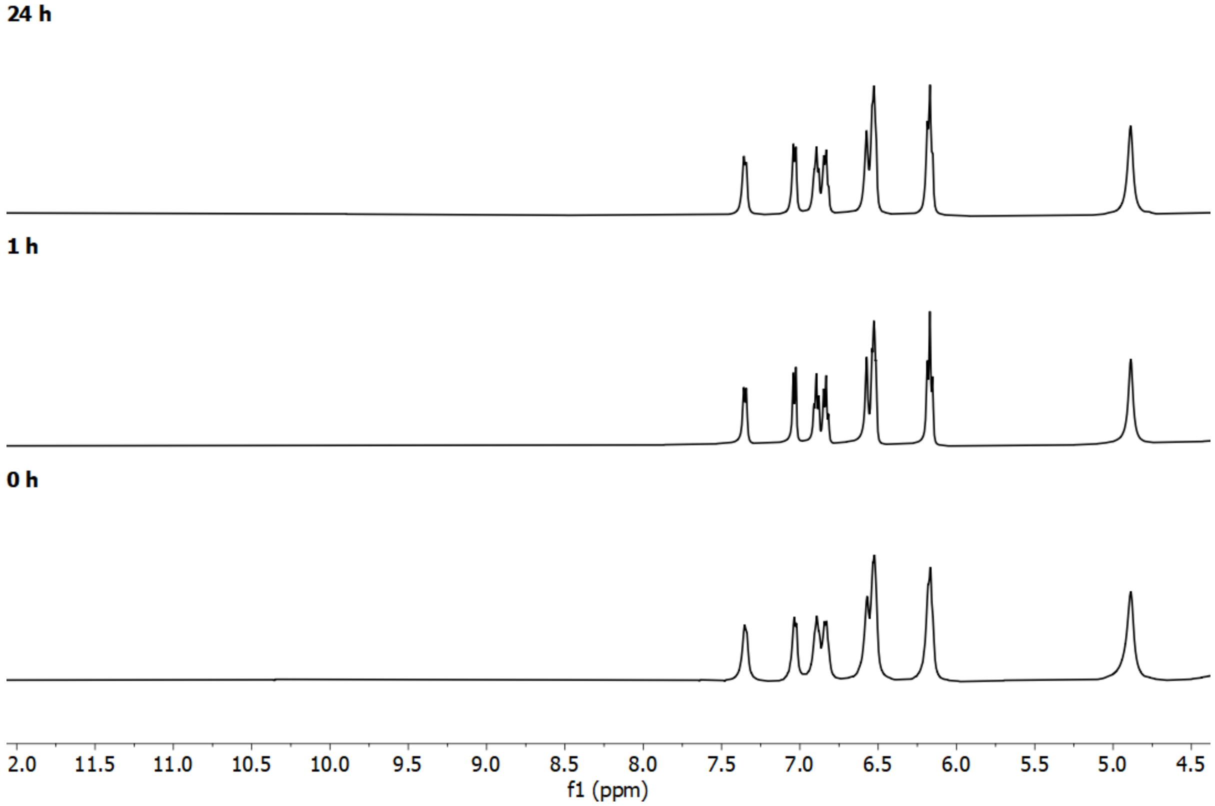

Figure S 46: ^{1}H NMR spectra of **$[Ag_2(L4)_2](PF_6)_2$** (7.0 mM) in D_2O:DMSO (30:70) when measured at 0, 1 and 24 h (500 MHz, 298 K)

UV/Vis Spectroscopy – Stability Studies

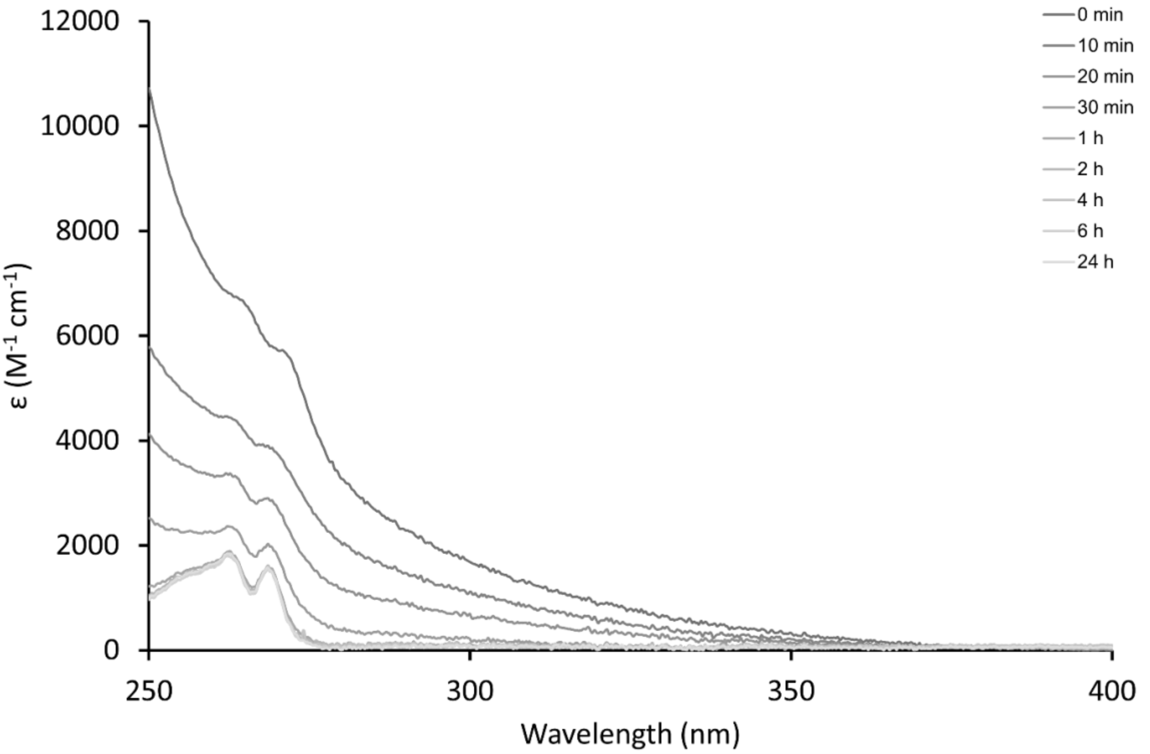

Figure S 47: UV/Vis spectra of **[Ag(L1)$_2$](PF$_6$)** (25 µM) in H_2O/DMSO (95:5) taken between 0 and 24 h

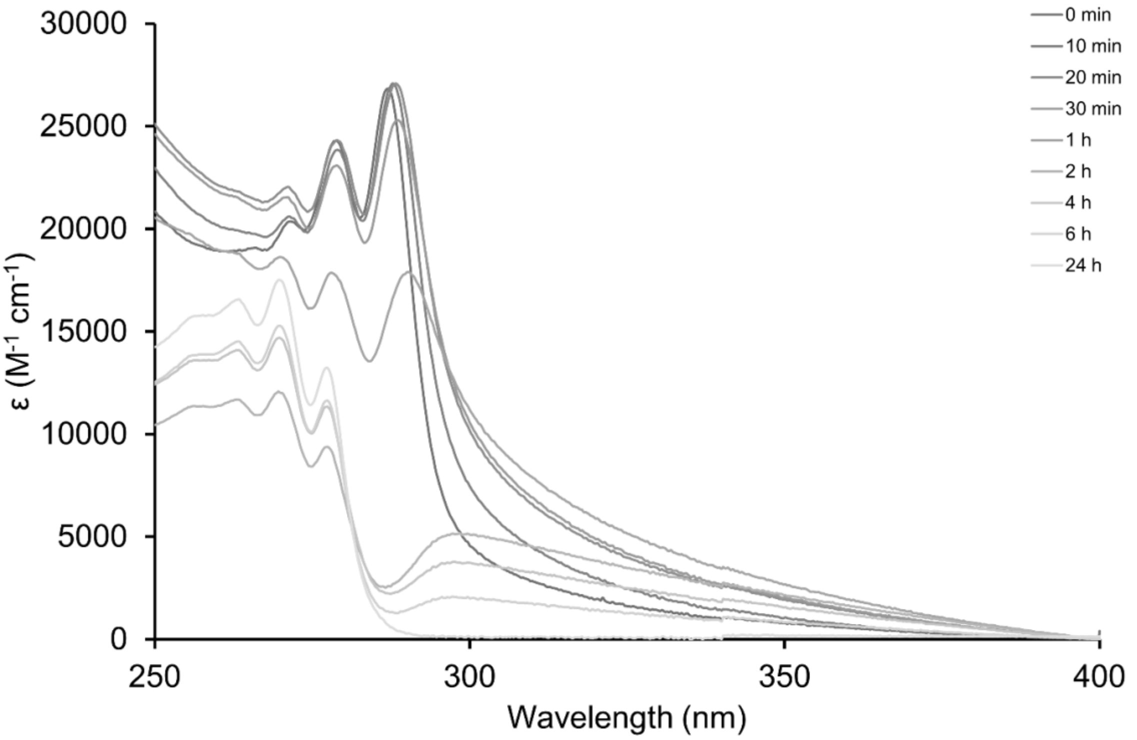

Figure S 48: UV/Vis spectra of **[Ag(L2)$_2$](PF$_6$)** (25 µM) in H_2O/DMSO (95:5) taken between 0 and 24 h

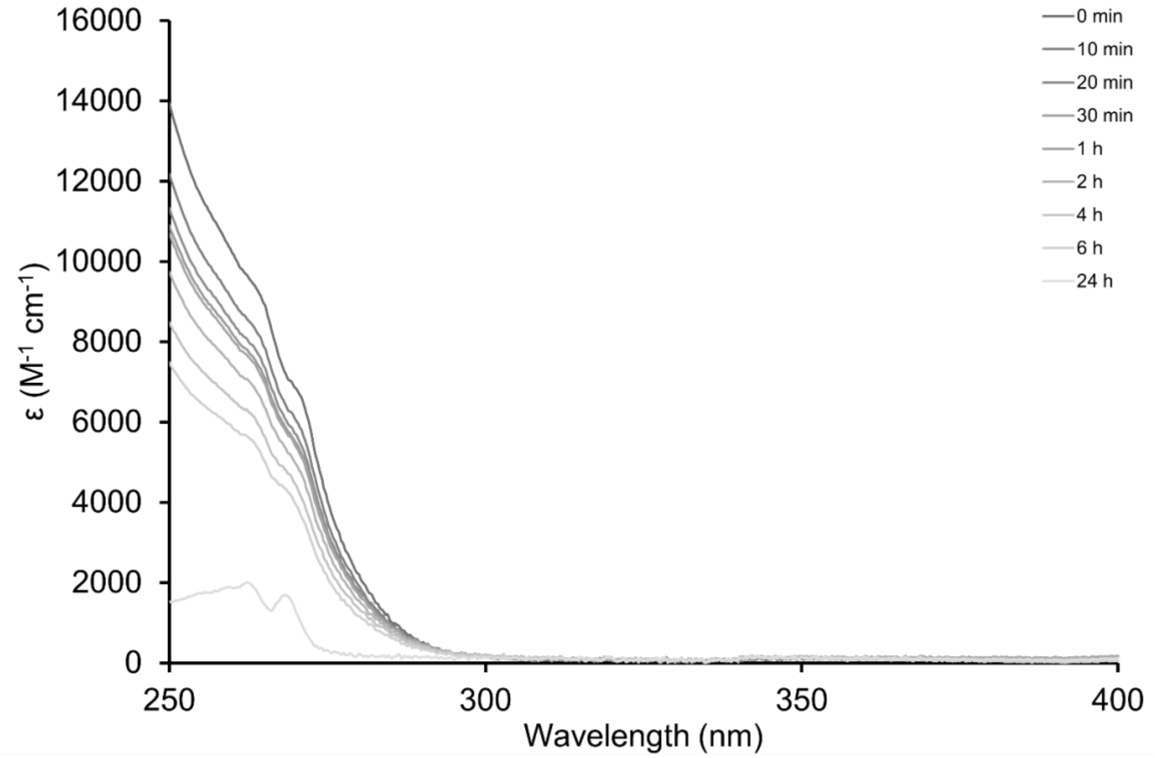

Figure S 49: UV/Vis spectra of **$[Ag_2(L3)_2](PF_6)_2$** (25 µM) in H_2O/DMSO (95:5) taken between 0 and 24 h

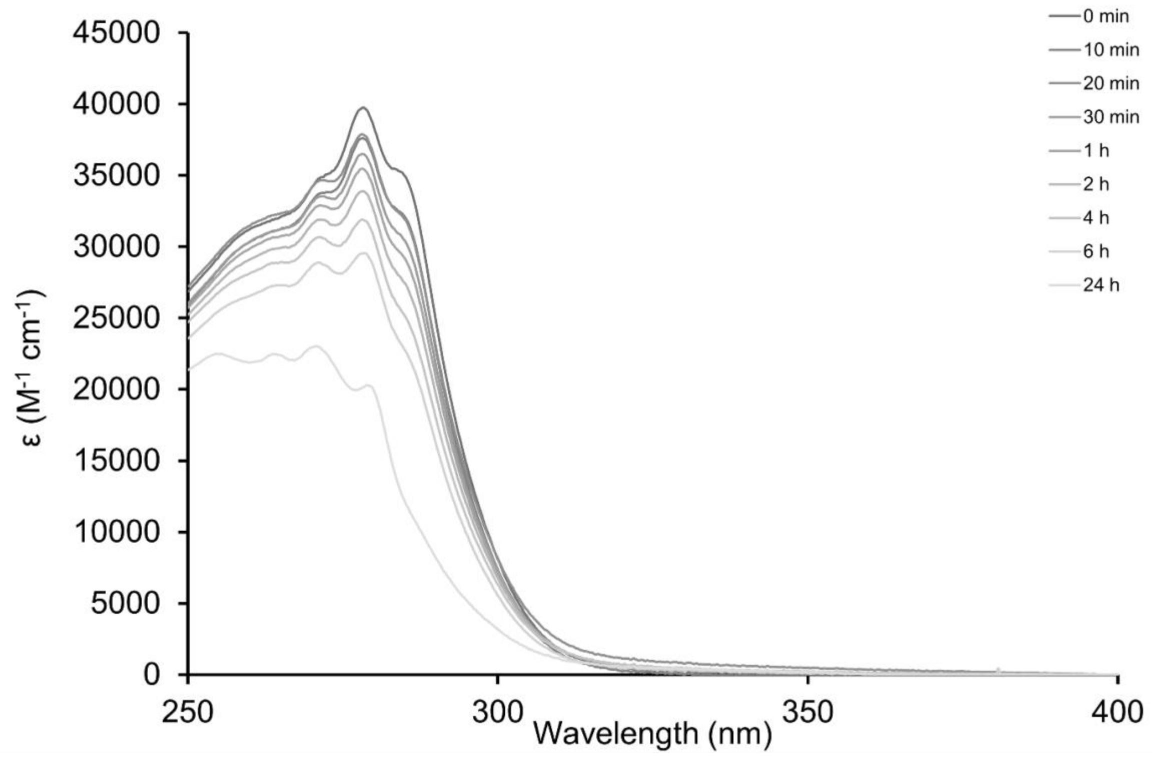

Figure S 50: UV/Vis spectra of **$[Ag_2(L4)_2](PF_6)_2$** (25 µM) in H_2O/DMSO (95:5) taken between 0 and 24 h

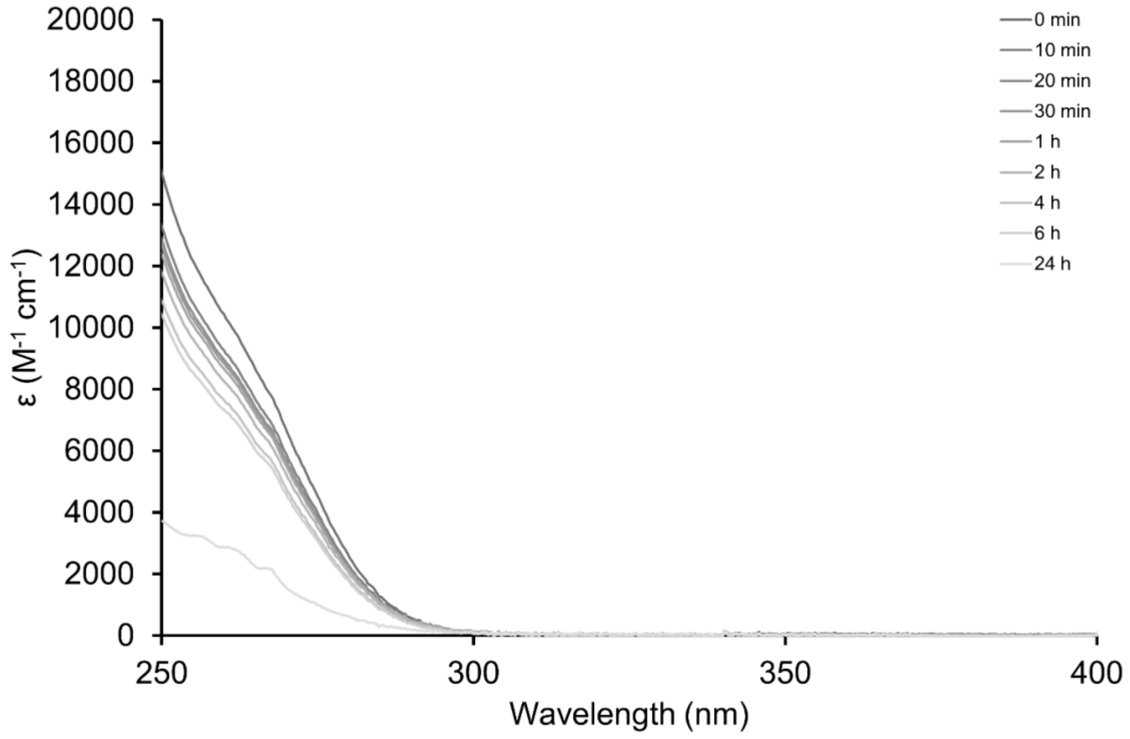

Figure S 51: UV/Vis spectra of **$[Ag_2(L5)_2](PF_6)_2$** (25 μM) in H_2O/DMSO (95:5) taken between 0 and 24 h

Reactive Oxygen Species

a)
b)
c)
d)

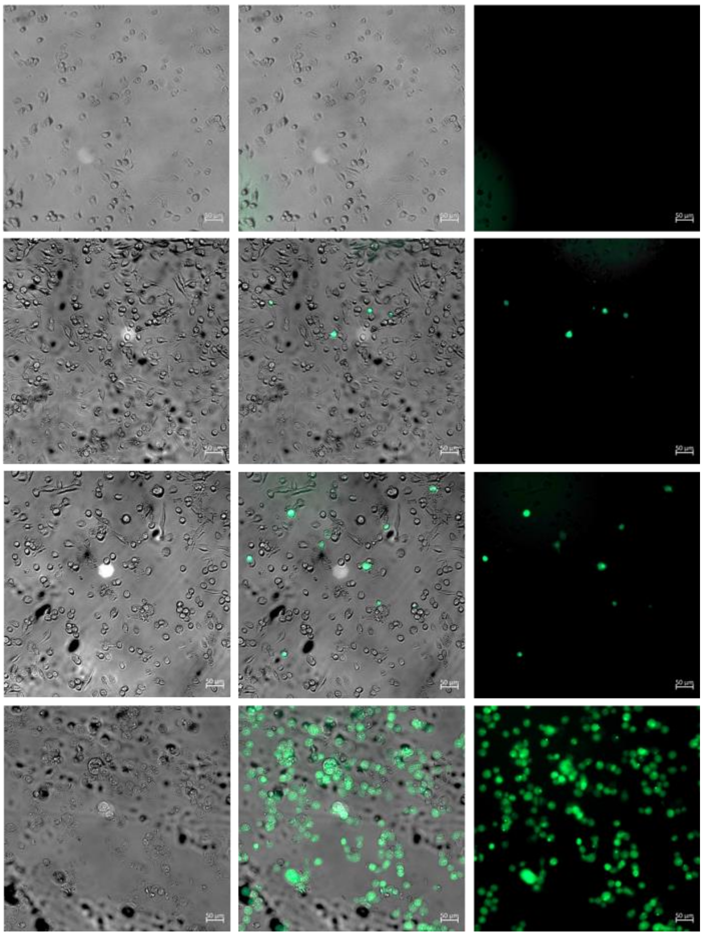

Figure S 52 Reaction oxygen species (ROS) observed after MDA-MB-231 cells were incubated with **[Ag_2(L4)$_2$](PF_6)$_2$** for 3.5 h at **a)** control, **b)** 1 x IC_{50} value, **c)** 2 x IC_{50} value and **d)** 5 x IC_{50} value followed by H_2DCFDA (20 μM) for 30 min. All images were taken using an Observer-7 microscope and recorded using brightfield (column 1), em/ex 494/512 nm (column 2) and the overlay (column 3).

EtBr titrations

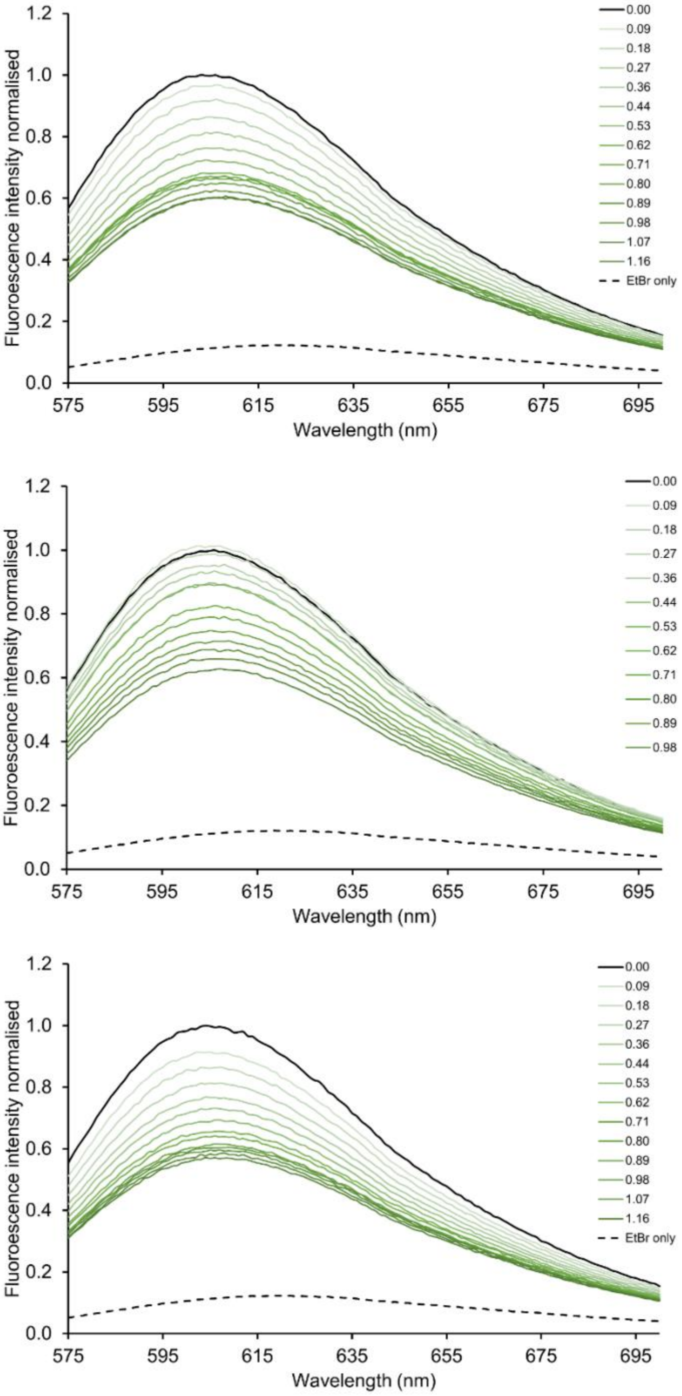

Figure S 53: Triplicate repeats of the fluorescence emission spectra for the sequential additions of **$[Ag_2(L4)_2](PF_6)_2$** to ct-DNA/EtBr (2.5/1): black line (no complex), light – dark green (0.09 – 1.16 molar equivalents of complex to EtBr) and dotted line (EtBr without ct-DNA)

Docking Studies

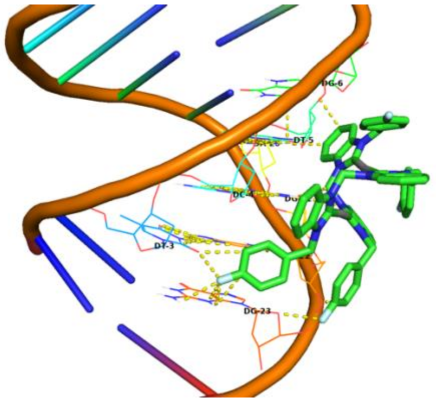

a)

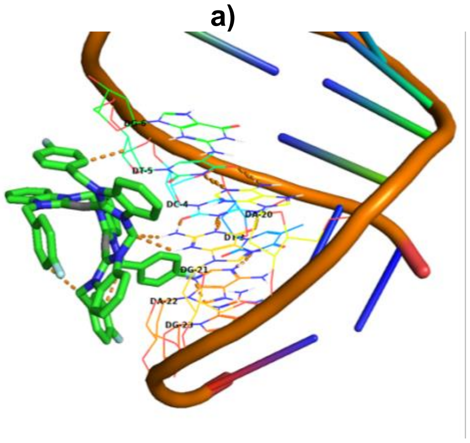

b)

Figure S 54: a) Close contacts (< 3 Å) between **$[Ag_2(L4)_2](PF_6)_2$** and DNA; **b)** clashes V.D.W.-distance ratio < 0.89
